# Targeted metabolite profiling of *Salvia rosmarinus* Italian local ecotypes and cultivars and inhibitory activity against *Pectobacterium carotovorum* subsp. *carotovorum*

**DOI:** 10.3389/fpls.2024.1164859

**Published:** 2024-02-02

**Authors:** Valeria Iobbi, Giuliana Donadio, Anna Paola Lanteri, Norbert Maggi, Johannes Kirchmair, Valentina Parisi, Giovanni Minuto, Andrea Copetta, Mauro Giacomini, Angela Bisio, Nunziatina De Tommasi, Giuliana Drava

**Affiliations:** ^1^ Department of Pharmacy, University of Genova, Genova, Italy; ^2^ Department of Pharmacy, University of Salerno, Fisciano, Italy; ^3^ Plant Pathology Laboratory, Section Microbiology and Molecular Biology, Centro di Sperimentazione e Assistenza Agricola (CeRSAA), Albenga, Italy; ^4^ Department of Informatics, Bioengineering, Robotics and System Science, University of Genova, Genova, Italy; ^5^ Department of Pharmaceutical Sciences, Division of Pharmaceutical Chemistry, University of Vienna, Vienna, Austria; ^6^ Research Centre For Vegetable and Ornamental Crops (CREA), Sanremo, Italy

**Keywords:** *Salvia rosmarinus*, NMR spectroscopy, multivariate data analysis, rosemary ecotypes, *Pectobacterium carotovorum* subsp. *carotovorum*

## Abstract

**Introduction:**

The development of agriculture in terms of sustainability and low environmental impact is, at present, a great challenge, mainly in underdeveloped and marginal geographical areas. The *Salvia rosmarinus* “Eretto Liguria” ecotype is widespread in Liguria (Northwest Italy), and farmers commonly use it by for cuttings and for marketing. In the present study, this ecotype was characterized in comparison with other cultivars from the same geographical region and Campania (Southern Italy), with a view to application and registration processes for the designation of protected geographical indications. Moreover, the possibility of using the resulting biomass after removing cuttings or fronds as a source of extracts and pure compounds to be used as phytosanitary products in organic farming was evaluated. Specifically, the potential of rosemary extracts and pure compounds to prevent soft rot damage was then tested.

**Methods:**

A targeted NMR metabolomic approach was employed, followed by multivariate analysis, to characterize the rosemary accessions. Bacterial soft rot assay and disk diffusion test were carried out to evaluate the activity of extracts and isolated compounds against *Pectobacterium carotovorum* subsp. *carotovorum.* Enzymatic assay was performed to measure the in vitro inhibition of the pectinase activity produced by the selected pathogen. Molecular docking simulations were used to explore the possible interaction of the selected compounds with the pectinase enzymes.

**Results and Discussion:**

The targeted metabolomic analysis highlighted those different geographical locations can influence the composition and abundance of bioactive metabolites in rosemary extracts. At the same time, genetic factors are important when a single geographical area is considered. Self-organizing maps (SOMs) showed that the accessions of “Eretto Liguria” appeared well characterized when compared to the others and had a good content in specialized metabolites, particularly carnosic acid. Soft rotting Enterobacteriaceae belonging to the *Pectobacterium* genus represent a serious problem in potato culture. Even though rosemary methanolic extracts showed a low antibacterial activity against a strain of *Pectobacterium carotovorum* subsp. *carotovorum* in the disk diffusion test, they showed ability in reducing the soft rot damage induced by the bacterium on potato tissue. 7-O-methylrosmanol, carnosol and isorosmanol appeared to be the most active components. In silico studies indicated that these abietane diterpenoids may interact with *P. carotovorum* subsp. *carotovorum* pectate lyase 1 and endo-polygalacturonase, thus highlighting these rosemary components as starting points for the development of agents able to prevent soft rot progression.

## 1 Introduction

*Salvia rosmarinus* Spenn. (Roma-Marzio and Galasso, 2019; POWO, 2023) is a Mediterranean aromatic, evergreen shrub widely cultivated for culinary, medicinal, and ornamental purposes. This species grows in open areas in a large diversity of dry, sunny, and calcareous habitats, from sea level up to 1,500 m (Rosselló et al., 2006). It is mainly distributed in the western half of the Mediterranean area, in Europe, and North Africa. It is rare in the eastern part of the Mediterranean and is regarded as introduced in the eastern most part of Europe. Based on this distribution, it is assumed that the center of diversification of the species is located in the South-Eastern Iberian Peninsula (Mateu-Andrés et al., 2013). *S. rosmarinus* is very polymorphic with high morphological variation in growth habit, leaf width, and flower color (Rosúa, 1986), and hybridization and introgression among subgeneric taxa (Drew et al., 2017) have been reported (Rosúa, 1986; Rosselló et al., 2006). Infra-specific taxa are maintained in the range of variability of the species, as the diversification reflects the existence of local ecotypes differing in adaptative traits (Rosselló et al., 2006). The systematics of varieties, subspecies, forms, races, biotypes, and ecotypes appear often uncertain and confused. Many of these variation patterns are available, with differences also in growth habits (upright, twisting, and creeping), and several of them grow wild in the Mediterranean countries (De Mastro et al., 2004). Currently, the distribution of biotypes, ecotypes, and landraces have been studied only in a few Italian areas (Mulas et al., 1998; Flamini et al., 2002; De Mastro et al., 2004), and the selection of cultivars and varieties adapted to local environmental conditions from spontaneous populations (Mulas and Mulas, 2005). The variability of the phytochemical profile of rosemary has been described as related to its phenological stage, geographic location, seasonal variation, environmental factors, abiotic stresses, and to genetic characteristics (Hidalgo et al., 1998; Tounekti and Munné-Bosch, 2012; Li et al., 2016). The chemical diversity of *S. rosmarinus* has been extensively studied, both as relates to the essential oil’s composition (Flamini et al., 2002; Gurbuz et al., 2016; Satyal et al., 2017) and to the antioxidant secondary metabolites (Wellwood and Cole, 2004). The abietane diterpenoids carnosic acid and carnosol, along with other diterpenoids, polyphenolic acids, and flavonoids, are considered rosemary extracts’ most relevant bioactive compounds (Cuvelier et al., 1996; Xiao et al., 2008; Birtić et al., 2015). Specifically, carnosic acid displays significant antioxidant properties, and it is of great interest in food, pharmaceutics, and cosmetic industries for antimicrobial, anti-inflammatory, anti-carcinogenic, anti-adipogenic (Birtić et al., 2015), and phytotoxic activities (Appiah et al., 2018). Carnosic acid has commercial interest as an approved food additive (Birtić et al., 2015), and the antioxidant activity of rosemary extracts is primarily associated with the amount of this compound. On this basis, the growers are rewarded based on the abundance of carnosic acid in the harvested biomass (Sahoo et al., 2022), and this compound is used to characterize rosemary accessions (Wellwood and Cole, 2004; Sahoo et al., 2022).

In Northwest Italy, wild populations of *S. rosmarinus* are characterized by upright habitus, elliptical light green leaves, and pale purplish-blue corolla. This ecotype, named ‘Eretto Liguria’, is distributed in the regional area, and farmers use it to collect germplasm as cuttings for the cultivation of marketed potted plants.

The present study’s first aim was to discriminate the ‘Eretto Liguria’ rosemary ecotype from other cultivars and varieties cultivated in the same area and another Italian geographical location, in different climatic zones, highlighting its phytochemical traits. NMR spectroscopy coupled with multivariate data analysis was applied to characterize the rosemary accessions, focusing on the main metabolites selected according to literature data (Xiao et al., 2008).

The second aim of this study was to point out new possibilities for the use of waste of rosemary chain. No official data on global rosemary production waste are available. At local level (Liguria region, Italy), considering an annual production of 37 million potted plants of rosemary (CeRSAA estimation, 2023) that are pruned two times during the cropping period, the produced pruning waste (vegetative tips and young branches 5–7 cm long) is estimated to be 370 t (CeRSAA GEP Testing Centre, 2022). To produce potted rosemary plants, growers apply integrated pest management regulations (DIR EU 128/2009) using low-toxicity chemicals characterized by a good residual profile and low environmental impact. At present, residual biomass from non-utilized parts of medicinal and aromatic plants is not regarded as waste, since it can be recycled and converted into value-added products (Saha and Basak, 2020) according to the principles of circular economy (Kirchherr et al., 2017). Specifically, the European Circular Economy Action Plan (European Union, 2020) focuses on the valorization of agricultural residues as a potential source of bioactive compounds (Skendi et al., 2022). Moreover, the Farm to Fork Strategy, within the European Green Deal, envisages a significant reduction of chemicals, whose target, by 2030, provides for an average decrease of 50% of chemicals compared to the current quantities. This target will be achieved through the revision of DIR EU 128/2009, which will impose an increased use of low-toxicity products, the rise in soil cultivation according to organic methods, and the increase in integrated pest management. Moreover, the use of natural-based products of natural origin and of botanical extracts, both used as plant protection products (EU REG 1107/2009) and as substances capable of increasing the defenses of plants against abiotic and biotic agents (Biostimulants—EU REG 1009/2019) will be strengthened. At present, the available products include natural-based products (i.e., Strobilurins and Azadiractins), natural compounds or plant extracts (i.e., eugenol, geraniol, orange oil, and spearmint oil) formulated as plant protection products and biostimulants extracts from plant biomass (i.e., sweet orange essential oil, blends of tannins, olive oil, cotton oil, propolis, Marseille soft soap, and wine/fruit vinegar). The market, in the latter case, is rapidly expanding in Europe (1.5 Bln € turnover; more than 2,000 employees; 10% of annual growth) and in Italy (50 Million € turnover; 10% of annual growth) (National Association of Fertilizer Manufacturers, 2023), and therefore, this sector appears to be extremely promising. *S. rosmarinus* extracts are used by some chemical companies (Benuzzi M. CBC (Europe) srl - division BIOGARD, 2023, personal communication) as a pathogen and pest control (REG. EU 1107/2009) or as biostimulants (REG. EU 1009/2019).

Considering the relevant production of waste biomass of *S. rosmarinus* in Liguria, finding a possibility of using extracts obtained from it in local agriculture could improve local, sustainable exploitation of the ‘Eretto Liguria’ ecotype, sold at fruit and vegetable markets as an aromatic plant for culinary use or for making cuttings. To this purpose, the activity against soft rot disease caused by *Pectobacterium carotovorum* subsp. *carotovorum* (syn. *Erwinia carotovora*) of the methanolic extracts was evaluated. *P. carotovorum* subsp. *carotovorum* is an enterobacterium infective to more than a hundred species of Brassicaceae, Solanaceae, Cucurbitaceae, and others, many of which are cultivated in the same Ligurian area. The bacterial soft rot induced by this microorganism during cultivation and storage is the cause of product loss in many vegetables chain. To our knowledge, there is no existing practical or effective chemical control for bacterial soft rot caused by *P. carotovorum* subsp. *carotovorum* (Mantsebo et al., 2014).

Many features of disease management caused by *Pectobacterium* spp. as cultivation measures, physical, chemical, and biological treatments for controlling soft rot were evaluated (van der Wolf et al., 2021). Analytical techniques for monitoring *P. carotovorum* were applied (Yang et al., 2021). Studies on various aspects of *P. carotovorum* virulence, ranging from quorum-sensing signal molecules, biofilm formation, regulation of expression of virulence genes by plant extracts and compounds, sensitivity to phage cocktails, and plant cell-wall-degrading enzymes (PCWDEs) production have been carried out (Põllumaa et al., 2012; van der Wolf et al., 2017; Gutierrez-Pacheco et al., 2018; Agyemang et al., 2020; Van Gijsegem et al., 2021a; Van Gijsegem et al., 2021b; Kim et al., 2022; Su et al., 2022). *Pectobacterium* spp. use pectin as a carbon source for growth and secrete multiple pectic enzymes, including pectate lyases (Pel), polygalacturonases (Peh), proteases (Prt), and cellulases (Cel), involved in the maceration of plant tissues (Hugouvieux-Cotte-Pattat et al., 2014; Agyemang et al., 2020; Mallick et al., 2022; Han et al., 2023). These enzymes were classified into various classes and subclasses depending on the substrate specificity and mode of action. According to the cleavage site, pectinases are divided into three groups: (i) hydrolases consisting of polygalacturonase, PG (EC 3.2.1.15); (ii) lyase/trans-eliminases comprising pectin lyase, PNL (EC 4.2.2.10), and pectate lyase, PL (EC 4.2.2.2); and (iii) pectin esterase, PE (EC 3.1.1.11) (Yadav et al., 2009). Plant signals and various transcriptional and post-transcriptional regulators coregulate the production of PCWDE (De Boer, 2003; Andresen et al., 2007; Agyemang et al., 2020). The PCWDE secretion is considered to be the main virulence factor employed by the pathogens to promote tissue maceration and rotting by disrupting the integrity of their host cells (De Boer, 2003; Põllumaa et al., 2012; Pun et al., 2021).

Plant extracts and compounds with the potential to prevent soft rot damage were reported (Yangui et al., 2013; Ashmawy et al., 2014; Sánchez-Hernández et al., 2023; Suaza-Gaviria et al., 2023). Among them, essential oils are well-known for their antimicrobial activity, and their possible application as coatings in storage and as preventive treatments against soft rot was evaluated (Zheng et al., 2013; Purnavab et al., 2015; Hajian-Maleki et al., 2019). Several pure plant constituents, including phenolic acids, quinones, flavonoids, terpenoids, and alkaloids, were examined for their efficacy in inhibiting bacterial growth, and their effect on several virulence factors, such as motility, biofilm formation, and exoenzyme activity (Joshi et al., 2015; Joshi et al., 2016; Gutierrez-Pacheco et al., 2018; Shaheen and Issa, 2020; Pun et al., 2021; Li et al., 2022).

Since the most abundant abietane diterpenoids of rosemary extracts (i.e., carnosic acid, carnosol, 7-*O*-methylrosmanol, 12-*O*-methylcarnosic acid, and isorosmanol) (Nieto et al., 2018) are characterized by a well-known antimicrobial activity (González, 2015), the present study also aimed to test these compounds against *P. carotovorum*. In this study, we focused on the depolymerizing enzymes of *P. carotovorum* subsp. *carotovorum* responsible for the random cleavage of the α-(1→4)-glycosidic bonds in the D-galacturonic acid moieties of the pectic substances: polygalacturonate lyase (pectate lyase 1) (PelA) and endo-polygalacturonase (PehA). The potential interactions of the selected abietanes with *P. carotovorum* subsp. *carotovorum* pectinase enzymes were explored using molecular docking simulations.

## 2 Materials and methods

### 2.1 Chemicals

Solvents, deuterium oxide (D_2_O, 99.9% D), CD_3_OD (99.8% D), and 3-(trimethylsilyl)propionic-2,2,3,3-*d_4_* acid sodium salt (TSP) were purchased from Sigma-Aldrich Chemical Company (Sigma-Aldrich, Milano, Italy).

### 2.2 Plant material

A total of 37 accessions of *S. rosmarinus* ecotypes and cultivars grown in field conditions in two Italian regions, Liguria (Northern Italy) and Campania (Southern Italy), all collected in the same period (June 2021), were used for the study (**Supplementary Tables S1–S4**). The identification of all the rosemary accessions was performed by Dr. Claudio Cervelli, according to the literature (Rosúa, 1986; Cervelli and Masselli, 2013; Drew et al., 2017). The vouchers of all the accessions were deposited at the Herbarium of Giardini Botanici Hanbury (La Mortola, Ventimiglia, Italy) (**Supplementary Table S2**).

### 2.3 Bacterial strain

*P. carotovorum* subsp. *carotovorum* was obtained from the collection of the Plant Pathology Laboratory of CeRSAA (accession number: CeRSAA Rdp 665/14). The strain satisfied Koch’s postulates, and its identity was confirmed by a specific molecular method (Khoshkdaman et al., 2008) and sequencing.

### 2.4 Sample collection and preparation

Fresh biomass (5–10 cm at the top of plant shoots), including leaves and stems (100 g each sample) were collected in May 2021, frozen and lyophilized in a freeze-dryer (Super Modulyo, Edwards, UK) for 48 h. Three biological replicates for each accession were used, with a total of 111 samples. All samples were sealed in plastic bags and stored dry in the dark until analysis. The dried material (approximately 35 g per sample) was then grounded. The powder samples for NMR analysis were prepared following the method reported by Tajidin et al. (2019). For each sample, 50 mg of dried leaves were extracted with a mixture of deuterated solvents made up of 250 µL of 90 mM H_2_KPO_4_ buffer in D_2_O (pH 6) and 650 µL of CD_3_OD, containing 0.01% 3-(trimethylsilyl) propionic-2,2,3,3-*d_4_* acid sodium salt (TSP), using a vortex mixer. The ultrasound-assisted extraction was performed for 30 min at room temperature using an ultrasound bath (Branson 2510E-MTH, Bransonic^®^, Milano, Italy), and centrifuged for 10 min at 13,000 rpm. The supernatant was then transferred into an NMR tube.

Methanolic extracts for antimicrobial and bacterial soft rot assays were prepared by stirring 5 g of dried biomass with 100 mL CH_3_OH ≥ 99.9% at 25°C at 150 rpm for 24 h and filtered through Whatman No. 4 paper (Sigma-Aldrich, Milano, Italy). The extraction was repeated three times. The combined methanolic extracts were evaporated to dryness under reduced pressure and stored at 4°C for further use.

### 2.5 NMR spectroscopy and processing

NMR data were acquired on a Bruker DRX-500 NMR spectrometer (Bruker BioSpin GmBH, Rheinstetten, Germany). The temperature was maintained at 30°C, and CD_3_OD was used as internal lock. Each ^1^H NMR spectrum consisted of 64 scans, 6.14 s acquisition time, relaxation delay (RD) of 4 s, mixing time of 0.01 s, and spectral width of 15.99 ppm. A presaturation sequence (NOESY-presat sequence, Bruker: noesygppr1d) was used to suppress the residual signal of water (Vignoli et al., 2019; Monzón Daza et al., 2021). A Chenomx 500 MHz custom library (CCL) (Chenomx NMR Suite 8.6, Chenomx Inc., Edmonton, 252 Canada) was set up using pure compounds both previously isolated in our laboratories from *S. rosmarinus* and from other plant sources and obtained from commercial sources (Sigma-Aldrich, Milano, Italy) (Xiao et al., 2008) (MSI level of identification according to Sumner et al. (2007) (**Supplementary Table S5**). A Chenomx Compound Builder tool was used. The custom library contained 27 metabolites. The CCL metabolites included abietane diterpenoids [carnosic acid (Pukalskas et al., 2005), carnosol (Pukalskas et al., 2005), isorosmanol (Topçu et al., 2013), epiisorosmanol (Pukalskas et al., 2005), 7-*O*-methylrosmanol (Richheimer et al., 1996), and 12-*O*-methylcarnosic acid (Richheimer et al., 1996; Pukalskas et al., 2005)], flavonoids [luteolin (Lin et al., 2015), scutellarein (Li et al., 2006), acacetin (King-Díaz et al., 2015), rutin (Napolitano et al., 2012), isorhamnetin-3-*O*-β-D-rutinoside (narcissin) (Rastrelli et al., 1995), quercetin (Napolitano et al., 2012), apigenin (Shen et al., 1993), apigenin-7-*O*-β-D-glycoside (Liu et al., 2012; Peng et al., 2016), catechin hydrate (Kiehlmann and Tracey, 1986), isorhamnetin-7-*O*-rutinoside (Mohamed et al., 2020), genkwanin (Gomes et al., 2011), diosmetin (Park et al., 2007), epicatechin (Abd El-Razek, 2007), and kaempferol (Napolitano et al., 2012)], and phenolic acids [rosmarinic acid (Lecomte et al., 2010), rosmarinate (Kuo et al., 2000; Correia et al., 2020), gallic acid (López-Martínez et al., 2015), ferulic acid (López-Martínez et al., 2015), caffeic acid (López-Martínez et al., 2015), *p*-coumaric acid (López-Martínez et al., 2015), and chlorogenic acid (Pauli et al., 1999)]. Additionally, 11 metabolites of the Chenomx 500 MHz version 11 library (CL), selected based on literature data (Xiao et al., 2008), were used (**Supplementary Table S6**). The CL metabolites included amino acids (alanine, valine, proline, and asparagine), carboxylic acids (acetate, malate, malonate, and fumarate), carbohydrates (fructose, sucrose), and choline (Xiao et al., 2008; Deborde et al., 2021). Each ^1^H NMR spectrum was acquired using the 1D. All spectra were acquired in triplicate. The metabolites were identified by comparing their ^1^H NMR spectra to those of the reference compounds in both the CCL and the CL libraries.

### 2.6 NMR data analysis

Quantitative analysis of NMR spectra was performed using NMRProcFlow 1.4.14 (INRA UMR 1332 BFP, Bordeaux Metabolomics Facility, Villenave d’Ornon, France) (Jacob et al., 2017) following the method reported by Grimaldi et al. (Grimaldi et al., 2021). Briefly, corrections of phasing and baseline were performed manually for all spectra using TOPSPIN version 3.2. All spectra were calibrated by using the internal standard at 0 ppm. Spectral area integration was made by variably sized bucketing using the online server NMRProcFlow. Buckets with a signal-to-noise ratio above 3 were selected for further analysis. The residual solvent regions of water (δ_H_ 4.65–4.75) were removed. Ppm ranges for each characterizing metabolite peak (**Supplementary Tables S5, S6**) were selected for quantification, and all the data needed were exported into a spreadsheet workbook using the “qHNMR” template. The data matrices generated by NMRProcFlow, one of 27 buckets (CCL compounds) and one of 11 buckets (CL compounds), were then subjected to multivariate analysis.

### 2.7 Multivariate data analysis

Classical statistical analysis was performed using Systat software for Windows Version 13 (Systat Software Inc., Chicago, IL, USA). The correlations among variables were calculated with the Pearson correlation coefficient. Hierarchical clustering (HC) analysis (single linkage as agglomeration method) was applied to detect groupings in variables (based on Pearson correlation coefficient as distance metric) and in samples (based on Euclidean distance) (Anderberg, 1973). Different data pre-treatment methods were tested, i.e., autoscaling, log transformation, and vast scaling (van den Berg et al., 2006). For explorative analysis, principal component analysis (PCA) with Varimax rotation was applied to the matrix of autoscaled data (Jackson, 2004). Statistical significance was set at *p* < 0.05. Self-organizing maps (SOMs) were then applied using Matlab R2022a and SOM toolbox 2.1 (The MathWorks, Inc., Natick, MA, USA) (Vatanen et al., 2015). SOMs belong to the category of unsupervised models and are a type of network organization of information processes. The log-transformed (van den Berg et al., 2006) data were then pre-processed before sending them to the algorithm, to prevent variables with a higher range from dominating the map due to their greater impact on the distances involved. A variance-based normalization of the data was performed. Subsequently, linear initialization and batch training were performed. The training took place in two steps: first, the rough phase with a larger radius and learning rate that also considers the most distant nodes is performed, and then, a refinement phase with a smaller radius and learning rate was done. After these steps, the U-matrix was generated, visualizing the distances between neighboring map units. Uniform areas identify clusters, while higher values indicate a cluster edge. The other maps represent the component plan (single compounds), and highly correlated variables show similar maps. Depending on the component values, hits are associated with a single unit of the map. Hits are the number of times a single map unit responds to inputs and indicate how input information is collected in each neuron.

### 2.8 Bacterial soft rot assay

Potato tubers, var. ‘Colomba’ were obtained from the production field of CeRSAA, in Albenga, SV, Italy. *P. carotovorum* subsp. *carotovorum* was obtained from the collection of the Plant Pathology Laboratory of CeRSAA (Accession number: CeRSAA Rdp 665/14). *P. carotovorum* subsp. *carotovorum* was cultured at 28°C on a nutrient agar medium [NYDA, made up of 8.0 g nutrient broth, 1.5 g glucose, 20.0 g agar, and 4.0 g yeast extract (Sigma-Aldrich, Milano, Italy), and 1 L deionized water] in Petri dishes for 2 days at 28°C. The bacterial inoculum was prepared from the 2-day-old cultured bacteria in a vial containing Buffered Pepton Water (Generon. Italy). The bacterial inoculum (1 × 10^8^ CFU/mL) was quantified by serial dilution plating method and stored at 4°C.

The assay on potato tuber slices was carried out following the methods reported in the literature (Mikiciński et al., 2010; Osei et al., 2022). Briefly, the tubers were first washed under tap water, dried with cleaning paper cloth, dipped for 5 s into 96% ethanol, air dried, and then flamed and cut into 10-mm-thick slices. The slices were placed on wet filter paper in Petri dishes. The bacterial inoculum was prepared from 2-day-old cultured bacteria in a vial containing Buffered Pepton Water (Generon, Modena, Italy) at a 10^8^ CFU/mL concentration. DMSO working solution was obtained by dissolving the extract in the solvent DMSO with the later addition of deionized water. Extracts and pure compounds were dissolved separately in a solution of sterile distilled water and DMSO working solution (1:1 v/v) (Sigma-Aldrich, Milano, Italy) to obtain, after inoculation, a concentration of 1,000 ppm (the preliminary test carried out to define the working concentration is reported in **Supplementary Table S9**). This solution was then inoculated with bacterial suspension to obtain a final concentration of 10^7^ CFU/mL of *P. carotovorum* subsp. *carotovorum*. Five minutes after the inoculation, filter paper disks 10 mm in diameter were soaked with 0.05 mL of this suspension. The filter paper disks were then placed at the center of the upper surface of each potato slice. After incubation at 25°C for 5 days, tissue rotting was determined, and the development of rot radius was measured. Each sample was tested on three potato slices. Control tubers were treated in the same way, but paper disks were soaked with a solution of inoculated sterile distilled water and DMSO working solution 1:1 v/v (positive control, PC) or with a solution of sterile distilled water and DMSO working solution 1:1 v/v (negative control, NC).

### 2.9 Disk diffusion test

A disk diffusion test was carried out to evaluate the extracts antimicrobial activity. Pure colonies of *P. carotovorum* subsp. *carotovorum* were suspended in a sterile saline solution with 0.9% sodium chloride (Generon, Modena, Italy) until a turbidity matched McFarland tube number of 0.5 (10^8^ CFU/mL). A loopful from the adjusted suspension was swabbed onto Muller Hinton agar (MH 2.0 g meat extract, 17.5 g hydrolyzed casein, 1.5 g starch, 17.0 g agar, and 1 L deionized water). Sterile paper disks (Whatman No. 40; 6 mm in diameter) were soaked in 10 μL of each diluted extract and pure compound (**Supplementary Table S10**), then dried at ambient temperature for 15 min, and positioned onto the surface of the inoculated agar plate. The plates were then incubated at 35°C for 24 h (Hemeg et al., 2020). Each extract and pure compound were tested in triplicates at 2,000 ppm, 1,000 ppm, 500 ppm, 250 ppm, 50 ppm, 16 ppm, and 0 ppm, respectively. The working solution (2,000 ppm) of each extract and pure compound was diluted in 10% DMSO; then, a parallel test with 10%, 5%, 2.5%, 1.25%, 0.25%, and 0.08% was conducted to verify the absence of sensitivity of the strain to DMSO. Ampicillin (Sigma Aldrich) at 1 ppm, 8 ppm, and 16 ppm were tested as positive control. The sensitivity of the tested microorganism to the different concentrations of extracts and pure compounds was assessed and classified by the diameter of the inhibition halos (growth inhibition zone including the 6 mm disk), as not sensitive for the diameters ≤ 8 mm, sensitive for diameters 9–15 mm, and very sensitive for diameters > 15 mm (Ponce et al., 2003). The extract and pure compounds were then classified into inactive, active, and very active substances (Steglińska et al., 2022). The results were expressed as the average of three independent repetitions with a standard deviation value.

### 2.10 Enzymatic activity assay

*In vitro* inhibition of the pectinase activity was measured using a commercially available enzymatic activity assay (QuantiChromTM Pectinase Assay Kit (DPEC-100), Bioassay Systems, Hayward, CA, USA). The turbidity, measured at OD 600 nm, is proportional to the amount of unhydrolyzed pectin, thus inversely proportional to the pectinase activity. The experiment was performed according to the manufacturer’s instructions, and all samples were assayed in triplicate. The bacterial inoculum of *P. carotovorum* subsp. *carotovorum* was prepared from 2-day-old cultured bacteria in a vial containing Buffered Pepton Water (Generon, Modena, Italy) at a 108 CFU/mL concentration. Pure compounds (carnosic acid, 12-*O*-methylcarnosic, 7-*O*-methylrosmanol, carnosol, and isorosmanol) were dissolved separately in a solution of sterile distilled water and DMSO working solution (1:1 v/v) to obtain, after the addition of the inoculum of *P. carotovorum* subsp. *carotovorum*, a concentration of 1,000 ppm and a final concentration of *P. carotovorum* subsp. *carotovorum* of 10^7^ CFU/mL. Five minutes after the inoculation, 0.02 mL of these suspensions and 0.02 mL of the pure inoculum at 10^7^ CFU/mL were transferred separately in a single well of a 96-well plate for the pectinase enzyme activity assay. The assay was then performed as recommended by the manufacturer’s instructions. Optical density (OD) was measured at 600 nm using the microplate reader ChroMate 4300 (Awareness Technology Inc., Palm City, FL, USA). The pectinase activity was then calculated according to the standard protocol of the manufacturer’s instructions, considering specific background readings of each solution.

### 2.11 Molecular docking

For *P. carotovorum* endo-polygalacturonase (PehA), Protein Data Bank (Berman et al., 2000) entry 1BHE was used to represent the structure of the enzyme (Pickersgill et al., 1998). For *P. carotovorum* pectate lyase 1 (UniProt ID: P0C1C0) (The UniProt Consortium, 2020), a homology model was derived from PDB entry 2EWE (Scavetta et al., 1999) with Prime, which is part of the Schrödinger Suite 2020-4 (Schrödinger, 2021). More specifically, the template protein and the target protein sequences were aligned with Prime (amino acid sequence identity of 68%). Next, a homology model was generated with Prime using default settings.

To evaluate and validate the modeled 3D structure, a Ramachandran plot was constructed using PROCHECK, on the SAVE server (http://services.mbi.ucla.edu/SAVES). The ProSA web server (https://prosa.services.came.sbg.ac.at/prosa.pHp) (Wiederstein and Sippl, 2007) was employed to calculate the Z-score and evaluate the consistency between the crystal structure of the template and modeled protein.

The protein structures were processed according to the following protocol: the protonation states were optimized at pH 8.0 ± 0.5 and pH 7.0 ± 2.0 for *P. carotovorum* pectate lyase 1 (PelA) and *P. carotovorum* endo-polygalacturonase (PehA), respectively. Disulfide-bond formation was carried out, and all water molecules were removed. The protein structures were then subjected to energy minimization with the OPLS3e force field (Harder et al., 2016) to converge heavy atoms to an RMSD of 0.3. The receptor grid generation tool of the Glide module (Schrödinger, 2021) (also part of the Schrödinger Suite) was used to define the grid boxes. As deduced from sequence similarity and site-directed mutagenesis studies, the protruding loops on one side of the parallel β helix form the pectolytic active site. A 20-Å^3^ grid box was centered on Arg239, which is reported as the catalytic moiety of *P. carotovorum* pectate lyase 1. In the case of *P. carotovorum* endo-polygalacturonase (PehA), the 20-Å^3^ grid box was centered on centroid formed between the conserved residues Asn201-Thr202-Asp203, Gly222-Asp223-Asp224, Gly250-His251-Gly252, and Arg280-Ile281-Lys282 (Pickersgill et al., 1998). The molecular structures of carnosic acid, carnosol, 7-*O*-methylrosmanol, 12-*O*-methylcarnosic acid, and isorosmanol were energetically minimized with LigPrep (Schrödinger, 2021) (also part of the Schrödinger Suite) using the OPLS3e force field. All possible tautomers and protonation states at a pH of 7.0 ± 1.0 were enumerated with LigPrep for each ligand. Docking simulations were carried out with Glide in SP and in XP mode.

## 3 Results

### 3.1 ^1^H-NMR spectra of rosemary accessions and metabolite identification

**Figure 1:**
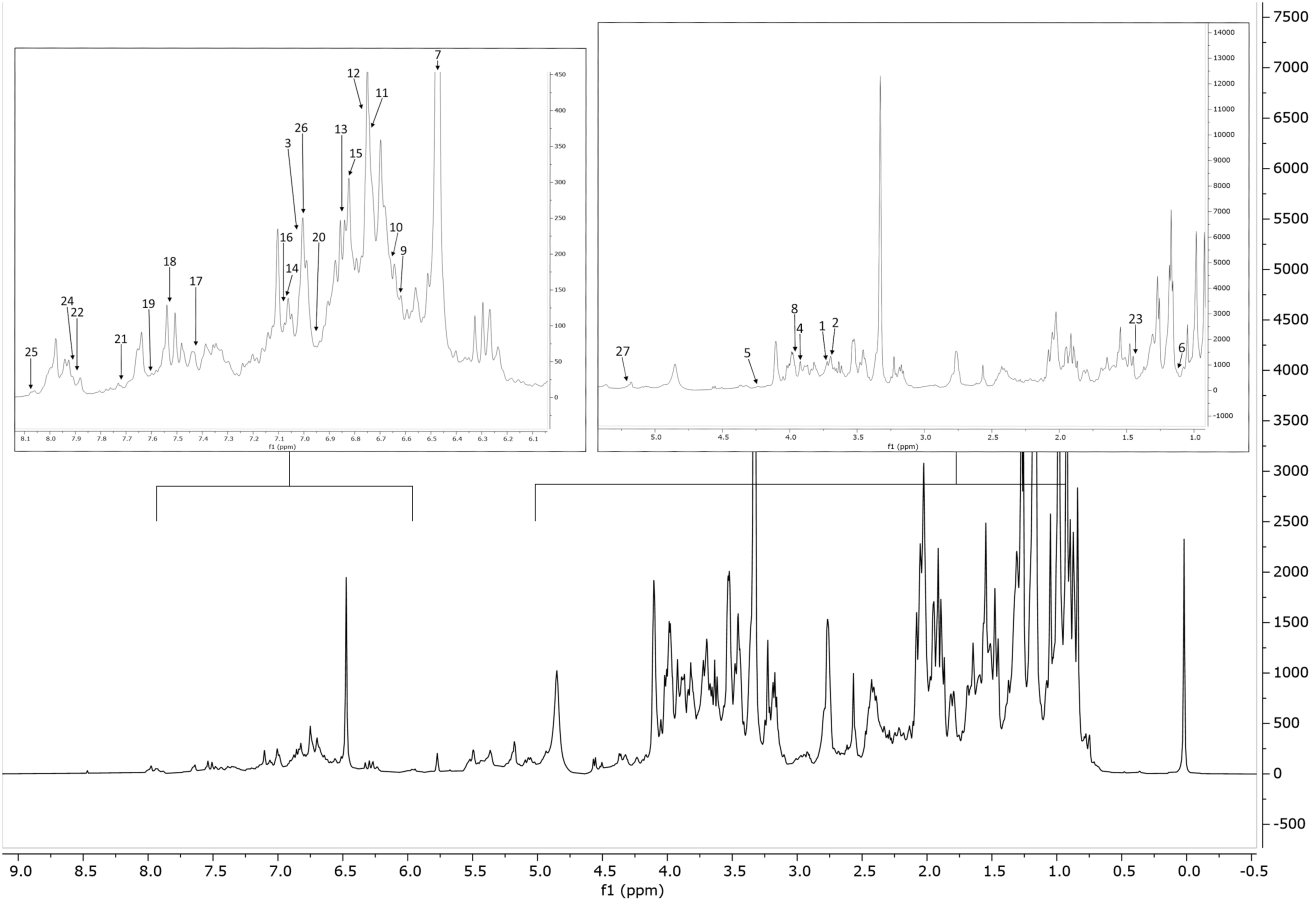
Representative ^1^H NMR spectrum of one extract of *S. rosmarinus* (accession 2, **Supplementary Table S1**), from *δ*_H_ 0.0 to 8.5 ppm, with annotation of the metabolites of the Chenomx 500 MHz custom library (CCL). Regions *δ*_H_ 0.9–2.1, *δ*_H_ 3.6–5.2, *δ*_H_ 6.0–8.1 (dotted boxes) were expanded. The peaks used for the quantification of the identified CCL metabolites are annotated with numbers. Keys: 1, 12-*O*-methylcarnosic acid; 2, 7-*O*-methylrosmanol; 3, methylrosmarinate; 4, genkwanin; 5, epicatechin; 6, isorhamnetin-3-*O*-β-D-rutinoside; 7, carnosic acid; 8, diosmetin; 9, luteolin; 10, scutellarein; 11, acacetin; 12, carnosol; 13, catechin hydrate; 14, gallic acid; 15, rosmarinic acid; 16, ferulic acid; 17, caffeic acid; 18, coumaric acid; 19, chlorogenic acid; 20, rutin; 21, quercetin; 22, apigenin; 23, isorosmanol; 24, apigenin-7-*O*-β-D-glycoside; 25, kaempferol; 26, isorhamnetin-3-*O*-rutinoside (narcissin); 27, epiisorosmanol.

**Figure 2:**
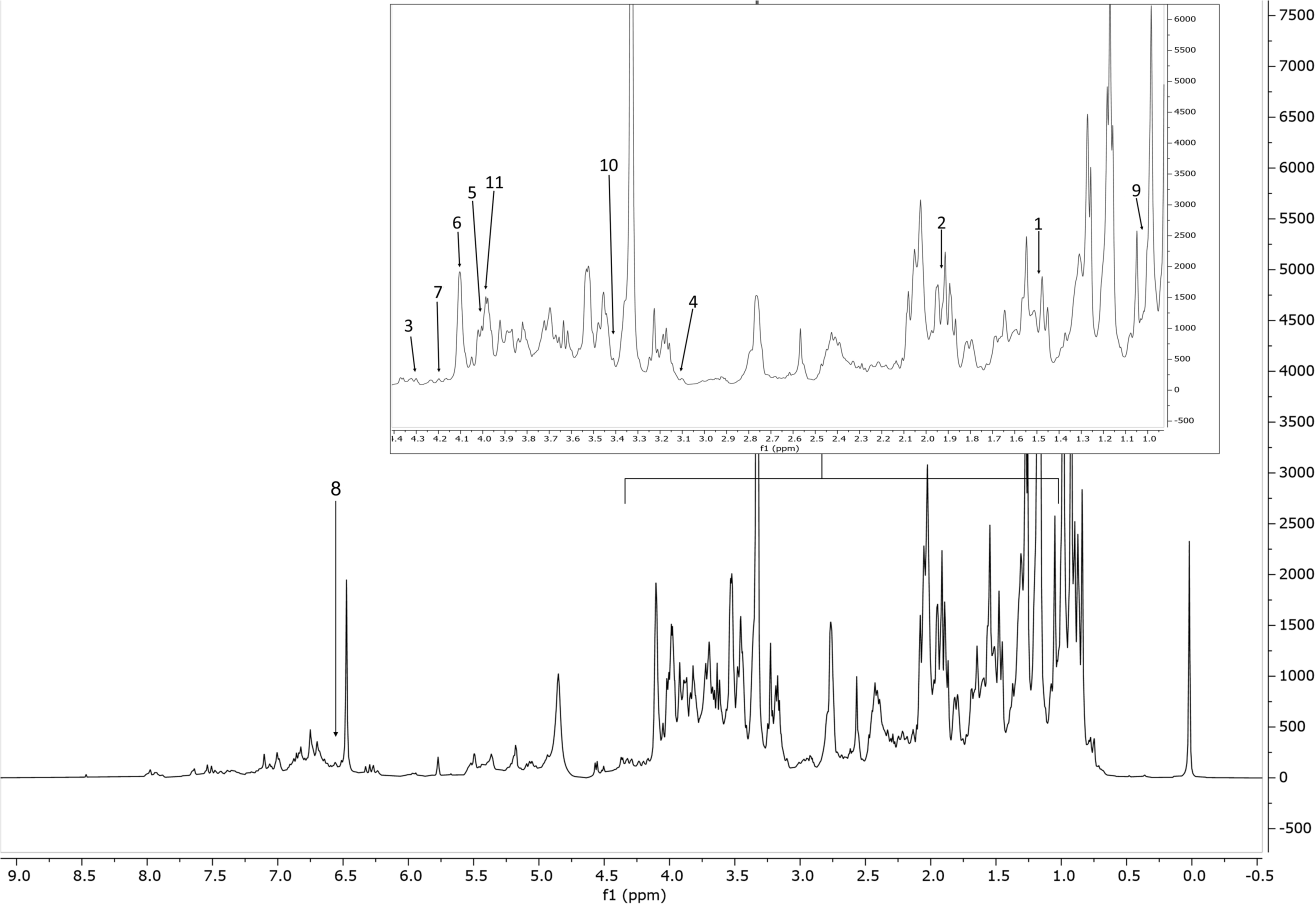
Representative ^1^H NMR spectrum of the extract of *S. rosmarinus* (accession 2, **Supplementary Table S1**), from *δ*_H_ 0.0 to 8.5 ppm with annotation of the metabolites of the Chenomx 500 MHz version 11 library (CL). Region *δ*_H_ 0.9–4.4 (dotted box) was expanded. The peaks used for quantification of the identified metabolites are annotated with numbers. Keys: 1, alanine; 2, acetate; 3, malate; 4, malonate; 5, choline; 6, fructose; 7, sucrose; 8, fumarate; 9, valine; 10, proline; 11, asparagine.

^1^H-NMR spectra were obtained for all the 111 samples. **Figures 1**, **2** show a ^1^H-NMR representative spectrum of one rosemary extract (accession 2, **Supplementary Table S2**). The complexity of the spectra can be visualized in some regions due to the overlapping of signals (**Supplementary Figure S5**). The spectral resonances were assigned based on the custom (CCL) and the 500 MHz version 11 (CL) Chenomx libraries. The identity of several metabolites was further confirmed through 2D NMR spectroscopic data (**Supplementary Figures S1–S5**). ^1^H-NMR spectra revealed the presence of the 38 major metabolites comprising primary and secondary ones mostly belonging to the chemical classes of amino acids, sugars, phenolic derivatives, and terpenoids (**Supplementary Tables S5, S6**). The NMR spectra showed signals at aliphatic (*δ*_H_ 0.7–3.0), carbohydrate (*δ*_H_ 3.0–5.5), and aromatic (*δ*_H_ 5.5–8.0) regions. The upfield (aliphatic) spectrum region from 0.4 to 3.0 ppm contained several overlapped signals mainly derived from amino acids (valine and alanine), acetate, and the methyl signals C16–C19 of diterpenoids. The mid-region from 3.0 ppm to 5.5 ppm was crowded with signals from amino acids (proline and asparagine), carbohydrates (fructose and sucrose), organic acids (malonate and malate), and choline. This spectral area showed an intense signal overlapping. The signals due to the anomeric protons of sugars were quite easily identifiable. The signals of methoxy groups of both diterpenoids [7-*O*-methylrosmanol *δ*_H_ 3.67 (s), 12-*O*-methylcarnosic acid *δ*_H_ 3.69 (s)] and polyphenolic compounds [acacetin *δ*_H_ 3.91 (s), ferulic acid δ_H_ 3.89 (s), diosmetin *δ*_H_ 3.94 (s), genkwanin *δ*_H_ 3.92 (s) isorhamnetin-7-*O*-rutinoside *δ*_H_ 3.95 (s), isorhamnetin-3-*O*-β-D-rutinoside (narcissin) *δ*_H_ 3.95 (s), methylrosmarinate *δ*_H_ 3.73 (s)), and the signals of choline methyls (*δ*_H_ 3.21(s)] were also present and mainly overlapped. The downfield region from 6.0 ppm to 8.5 ppm exhibited signals due to the aromatic resonances, mainly of phenolic acids (caffeic, chlorogenic, *p*-coumaric, gallic, and ferulic acids), rosmarinic acid and methylrosmarinate, flavonoids (acacetin, apigenin, apigenin-7-*O*-β-D-glycoside, catechin hydrate, diosmetin, epicatechin, genkwanin, isorhamnetin-3-*O*-β-D-rutinoside (narcissin), kaempferol, luteolin, quercetin, rutin, and scutellarein), the *trans*-olefinic protons of phenylpropanoids (rosmarinic acid *δ*_H_ 7.50 and *δ*_H_ 6.29, methylrosmarinate *δ*_H_ 7.56 and *δ*_H_ 7.29, ferulic acid *δ*_H_ 7.46 and *δ*_H_ 6.34, caffeic acid *δ*_H_ 7.42 and *δ*_H_ 6.27, coumaric acid *δ*_H_ 7.52 and *δ*_H_ 6.32, and chlorogenic acid *δ*_H_ 7.59 and *δ*_H_ 6.34) all doublets and with coupling constants *J* = 15.9–16.0 Hz), and the aromatic protons of diterpenoids [isorosmanol *δ*_H_ 6.98 (s), epiisorosmanol *δ*_H_ 6.81 (s), 7-*O*-methylrosmanol *δ*_H_ 6.79 (s), 12-*O*-methylcarnosic acid *δ*_H_ 6.48 (s), carnosol *δ*_H_ 6.73 (s), and carnosic acid *δ*_H_ 6.44 (s)].

### 3.2 Multivariate data analysis

To characterize the ‘Eretto Liguria’ ecotype, both accessions (ecotypes and cultivars) from the same geographical area, in North Italy, and accessions from another area, in South Italy, were considered. The relationships among the compounds detected in the rosemary accessions were investigated by correlation analysis of the selected metabolites comprised in the custom (CCL) and the 500 MHz version 11 (CL) Chenomx libraries, considered both jointly and separately. Only a few metabolites of the CL showed significant correlations (**Supplementary Table S7**). Cluster analysis allowed us to visualize them as a similarity dendrogram, pointing out a cluster formed by asparagine, proline, and choline (**Supplementary Figure S6A**). Several significant correlations were observed among the CCL metabolites (**Supplementary Table S8**), as shown in **Supplementary Figure S1B**: nine metabolites [methylrosmarinate, acacetin, carnosol, rosmarinic acid, ferulic acid, coumaric acid, chlorogenic acid, rutin, and isorhamnetin-3-*O*-rutinoside (narcissin)] joined into a cluster. Other two groups of correlated variables were formed by the apigenins and kaempferol, and by genkwanin, 7-*O*-methylrosmanol, diosmetin, and 12-*O*-methylcarnosic acid, respectively. No relevant information was added when CL and CCL metabolites were studied all together (**Supplementary Figure S7**). When cluster analysis was applied to the dataset of the CL metabolites to detect groupings among the samples, almost all the accessions from the Ligurian geographical area joined into a large group except for a few outliers (e.g., 9 and 22, and one of the three samples of 18, 27, and 32 accessions). Among the samples from Campania, only one sample of the 37 accession was included in the large cluster of Ligurian samples (**Supplementary Figure S8**). This grouping was substantially confirmed for CCL metabolites, with similar patterns of outliers; only two samples of 33 and 34 accessions from Campania showed similarity with Ligurian samples, as shown in **Supplementary Figures S9, S10**, where all the 38 variables (CL and CCL metabolites) were jointly considered.

Explorative analysis of the dataset was performed by PCA, which represents in a few plots the grouping of the accessions, the correlations among the metabolites, and the relationships between accessions and metabolites. **Supplementary Figure S11** shows the biplot of the 37 accessions described by the 11 CL metabolites on the plane of the first two PCs, explaining the 52% of the total variance. Accessions 36 and 37 (six samples), corresponding to cultivars not represented among the Ligurian samples, were characterized by low content of alanine, valine, and fumarate and high content of proline, choline, and asparagine. On the plane of the third and fourth PCs (29% of variance), low contents of fructose and sucrose characterized all the accessions from Campania (**Supplementary Figure S12**).

PCA performed on the 37 accessions described by the 27 CCL metabolites was also strongly affected by the difference between the two geographical regions. The presence of outliers (accessions 36 and 37 from Campania) was evident on the plane of the first two PCs, explaining the 51% of the total variance (**Supplementary Figure S13**). On the plane of the third and fourth PCs (17% of the variance), one accession from Campania at high content of luteolin and isorosmanol had a very strong leverage effect, making the visualization of the information contained in the data difficult (**Supplementary Figure S14**). The biplot on the fifth and sixth PCs (14% of the variance) showed that the accessions from Campania formed a separated group, at low content of carnosic acid, isorhamnetin-7-*O*-rutinoside, and epicatechin, and high content of epiisorosmanol and isorosmanol (**Supplementary Figure S15**). For these reasons, the data analysis was repeated excluding the accessions from Campania, to improve the analysis of the accessions from Ligurian region.

**Figure 3:**
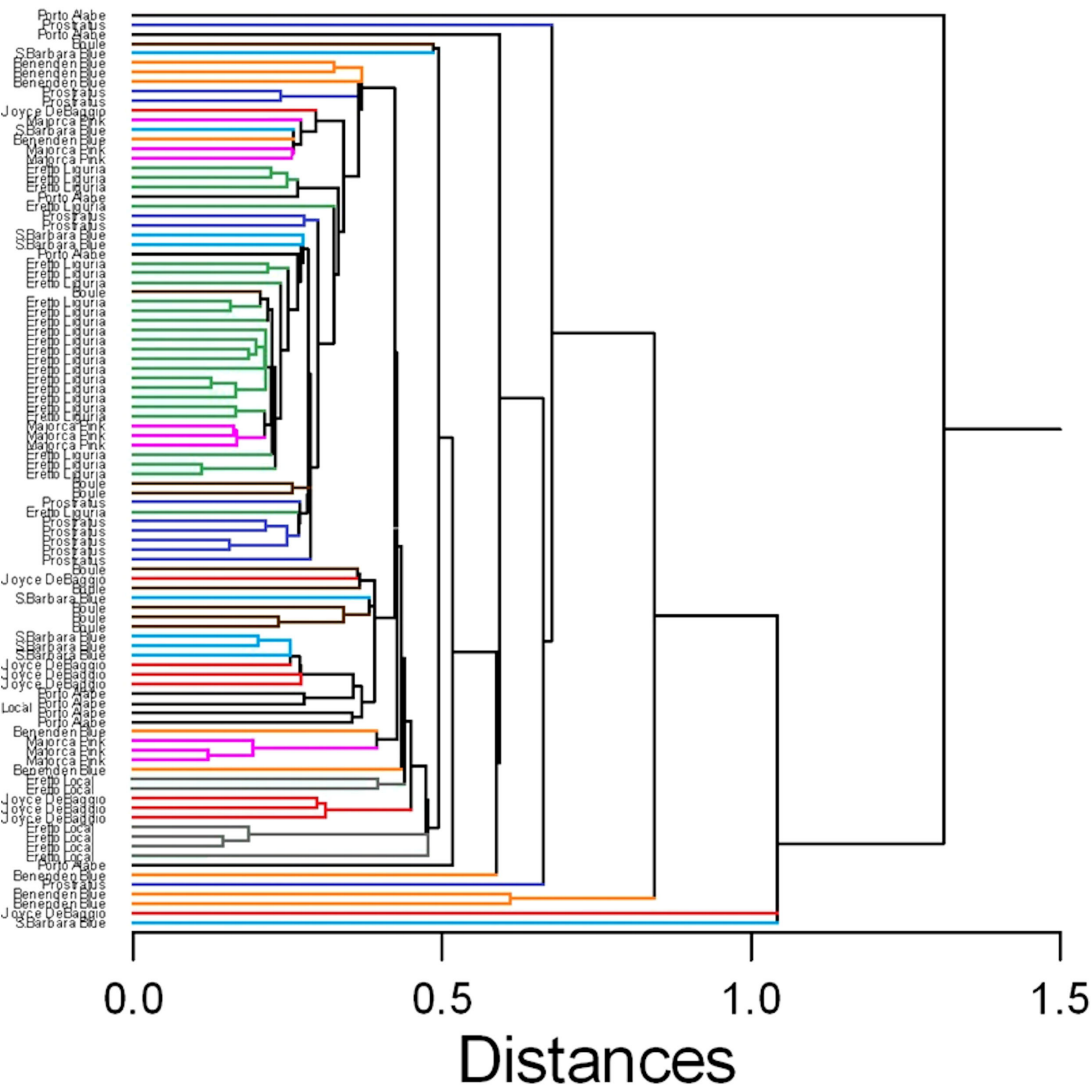
Similarity dendrogram of the 96 rosemary samples collected in Liguria. Hierarchical cluster analysis was applied to the 27 Chenomx 500 MHz custom library metabolites (CCL), using single linkage method based on Euclidean distance. The different ecotypes/cultivars are indicated by the name and shown with different color.

**Figure 4:**
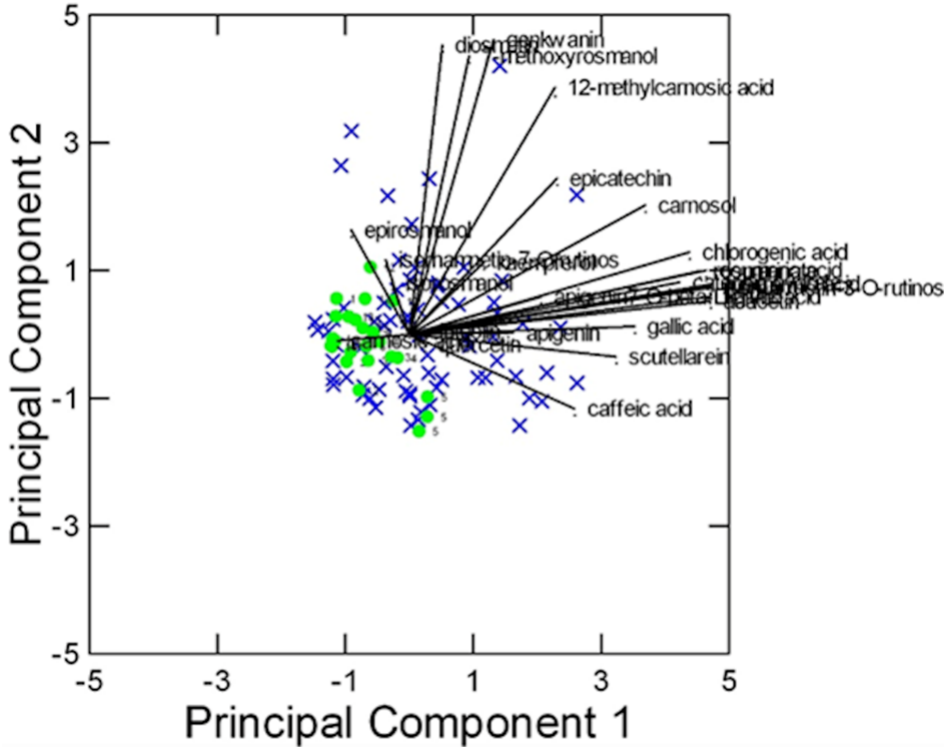
Results of PCA of the 27 Chenomx 500 MHz custom library metabolites (CCL) measured in 96 samples: biplot of Principal Components 1 and 2 (53% of explained variance). 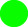 = “Eretto Liguria”; × = other ecotypes/cultivars.

**Figure 5:**
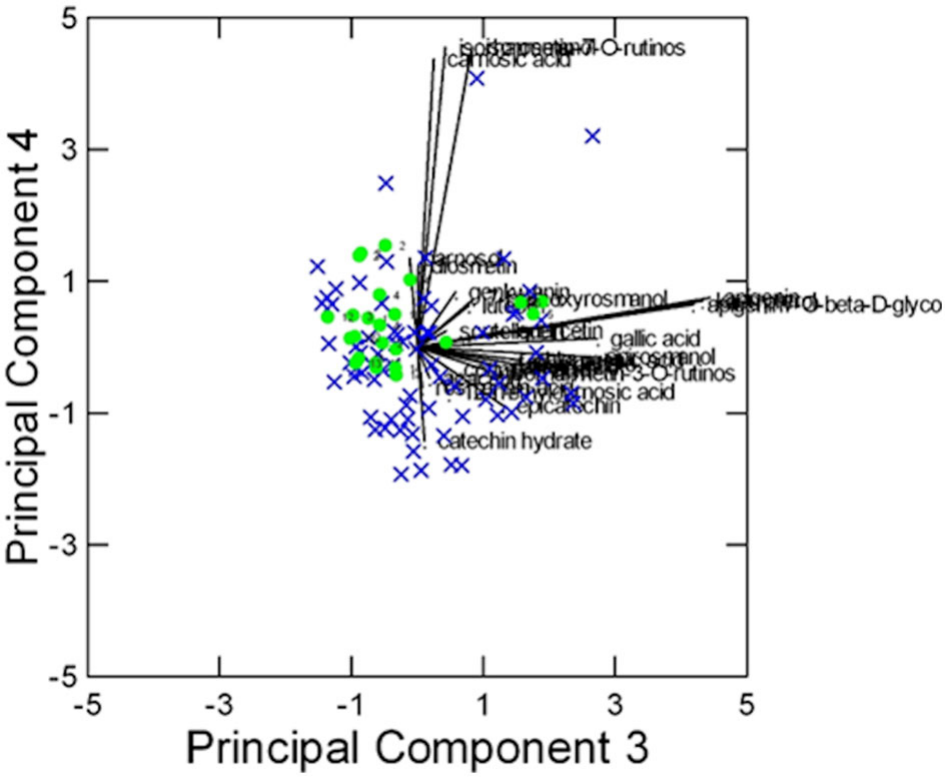
Results of PCA of the 27 Chenomx 500 MHz custom library metabolites (CCL) measured in 96 samples: biplot of Principal Components 3 and 4 (24% of explained variance). 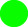 = “Eretto Liguria”; × = other ecotypes/cultivars.

In this case, despite some degree of overlapping among the accessions, the ‘Eretto Liguria’ appeared characterized, as shown by the dendrogram in **Figure 3**, where these accessions (green color) were grouped in one cluster including only a few other accessions, and by the biplots of PCA (**Figures 4**, **5**) on the plane of the first four PCs. ‘Eretto Liguria’ was characterized by low content of the variables at high loading on PC1, i.e., methylrosmarinate; acacetin; catechin hydrate; rosmarinic, ferulic, coumaric, and chlorogenic acids; rutin; and isorhamnetin-3-*O*-rutinoside. The variables at high loading on PC2 (12-*O*-methylcarnosic acid, 7-*O*-methylrosmanol, genkwanin, and diosmetin) were not discriminant among the accessions, and the high content of these metabolites allowed the detection of a few anomalous samples. Three CCL metabolites showed high loading on PC4, i.e., carnosic acid, isorhamnetin-7-*O*-rutinoside, and isorosmanol. Most of the ‘Eretto Liguria’ samples showed a higher content of carnosic acid with respect to other accessions such as ‘Santa Barbara Blue’, ‘Boule’, and ‘Joyce DeBaggio’.

The results obtained by SOMs were in agreement with those of PCA (**Supplementary Figures S16–S21**). The number of clusters was assessed based on the k-means algorithm and on the Davies–Bouldin index (DBI) (Davies and Bouldin, 1979). This index allows the identification of the most reliable number of clusters that corresponds to a minimum value of DBI (**Supplementary Figures S18A, S21A**). CL metabolites appeared less important to characterize the ecotype ‘Eretto Liguria’, as it was present in five of the six clusters, mainly characterized by acetate, sucrose, and fumarate (**Supplementary Figures S17, S18**). As for CCL metabolites, all the accessions of ‘Eretto Liguria’ were in the bottom cluster of the map, except for only one accession (5) (**Supplementary Figures S20, S21**). A higher content of carnosic acid characterized the neurons containing these accessions compared with the other clusters (**Supplementary Figures S19, S20**). In addition, accession 5 showed a high carnosic acid content (**Supplementary Figures S19**, C7, **Figure S20**). The other ecotypes/cultivars occupied the top part of the map at high content of methylrosmarinate, acacetin, carnosol, rosmarinic acid, ferulic acid, coumaric acid, chlorogenic acid, rutin, and isorhamnetin-3-*O*-rutinoside (narcissin), i.e., the group of highly correlated variables (**Supplementary Figure S6B**). The Ligurian accessions did not show significantly different relative amounts of abietane diterpenoids. Carnosic acid was the only compound that appeared to characterize the ‘Eretto Liguria’ ecotype.

### 3.3 Activity against bacterial soft rot

A total of 12 representative Ligurian rosemary accessions (i.e., ‘Eretto Liguria’ (1, 3, and 5), ‘Prostratus’ (Prostrata Group) (6 and 9), ‘Eretto’ (local ecotype) (7), ‘Boule’ (Prostrata Group, ‘Rampant Boule’) (16), ‘Joyce DeBaggio’ (20), ‘Benenden Blue’ (22), ‘Majorca Pink’ (26), ‘Porto Alabe’ (28), and ‘Santa Barbara Blue’ (32) (**Supplementary Table S1**), were selected for bacterial soft rot assay. The exposure for 5 min of a liquid suspension of *P. carotovorum* subsp. *carotovorum* (10^7^ CFU/mL) to a solution of the methanolic extracts of the selected rosemary accessions at the concentration of 1,000 ppm inhibited the bacterial soft rot at values ranging from 20.8% to 100%, compared to the untreated control (PC). The extracts 1, 6, 16, 20, 22, 26, 28, and 32 showed efficacy values (Abbott index) ranging from 76.9% to 100% (**Table 1**). These values were significantly different from those obtained with extracts 3, 5, 7, and 9, although 7 and 9 showed efficacy values in a medium range (63.7%). Pure abietane diterpenoids, i.e., isorosmanol, 12-*O*-methylcarnosic acid, carnosic acid, carnosol, and 7-*O*-methylrosmanol, characteristic of rosemary extracts, were then tested. The exposure for 5 min of a liquid suspension of *P. carotovorum* subsp. *carotovorum* (10^7^ CFU/mL) to a solution of isorosmanol, carnosol, and 7-*O*-methylrosmanol, at the concentration of 1,000 ppm, completely inhibited the bacterial soft rot. At the same test conditions, 12-*O*-methylcarnosic acid inhibited the bacterial soft rot by 70.3%, and carnosic acid by 30%, compared to the untreated control (PC) (**Table 2**) (**Supplementary Figure S22**).

**Table 1:** Evaluation of the activity against bacterial soft rot induced by *Pectobacterium carotovorum* subsp. *carotovorum* after treatments with methanolic extracts of rosemary accessions *^a^*.

Extracts	Concentration (ppm)	Mean ± SEd of the rot radius (mm)	Abbott index (%)e
1	1000	2.0±1.7 ^a^	80.2
3	1000	8.0±1.7 ^cd^	20.8
5	1000	7.3±2.5 ^bcd^	27.4
6	1000	0.7±0.6 ^a^	93.4
7	1000	3.7±2.3 ^ab^	63.7
9	1000	3.7±2.3 ^ab^	63.7
16	1000	1.3±1.2 ^a^	86.8
20	1000	2.0±0.0 ^a^	80.2
22	1000	0.0±0.0 ^a^	100
26	1000	1.0±0.0 ^a^	90.1
28	1000	0.0±0.0 ^a^	100
32	1000	2.3±1.5 ^a^	76.9
DW (PC)*^b^*	0	10.1±0.9 ^d^	0
DW (NC)*^c^*	0	0.0±0.0 ^a^	100

^a^ 1: “Eretto Liguria”; 3: “Eretto Liguria”; 5: “Eretto Liguria”; 6: ‘Prostratus’ (Prostrata Group); 7: “Eretto” (local ecotype); 9: ‘Prostratus’ (Prostrata Group); 16: ‘Boule’ (Prostrata Group, ‘Rampant Boule’); 20: ‘Joyce DeBaggio’; 22: ‘Benenden Blue’; 26: ‘Majorca Pink’; 28: “Porto Alabe”; 32: ‘Santa Barbara Blue’, **Supplementary Table S1**, **Supplementary Material**. Values are reported as average of the rot radius (mm) measured during the 3 trials. The test was repeated 3 times and performed in 3 replicates (I, II, III: data not reported). ^b^ DW (PC): inoculated distilled water/DMSO working solution 1:1 v/v (Positive Control). ^c^ DW (NC): not inoculated distilled water/DMSO working solution 1:1 v/v (Negative Control). ^d^ Different letters indicate significant difference among treatments (p ≤ 0.05, Tukey HSD test). ^e^ Abbott index % = [(Average in mm of rot radius of PC - Average in mm of rot radius) x 100] / Average in mm of rot radius of PC.

**Table 2:** Evaluation of the activity against bacterial soft rot induced by *Pectobacterium carotovorum* subsp. *carotovorum* after treatments with rosemary abietane diterpenoids.

Pure compounds	Concentration (ppm)	Mean±SEc of the rot radius (mm)	Abbott index (%)d
carnosic acid	1000	7.0±0.0 ^c^	30.7
carnosol	1000	0.0±0.0 ^a^	100
7-*O-*methylrosmanol	1000	0.0±0.0 ^a^	100
isorosmanol	1000	0.0±0.0 ^a^	100
12-*O-*methylcarnosic acid	1000	3.0±1.0 ^b^	70.3
DW (PC)*^a^*	0	10.1±0.9 ^d^	0
DW (NC)*^b^*	0	0.0±0.0 ^a^	100

The test was repeated 3 times and performed in 3 replicates (I, II, III: data not reported). ^a^ DW (PC): inoculated distilled water/DMSO working solution 1:1 v/v (Positive Control). ^b^ DW (NC): not inoculated distilled water/DMSO working solution 1:1 v/v (Negative Control). ^c^ different letters indicate significant difference among treatments (p ≤ 0.05, Tukey HSD test). ^d^ Abbott index % = [(Average in mm of rot radius of PC - Average in mm of rot radius) x 100] / Average in mm of rot radius of PC.

### 3.4 *In vitro* evaluation of antimicrobial activity against *Pectobacterium carotovorum* subsp. *carotovorum*

Although the effect of the selected extracts and pure compounds on the bacterial growth was previously evaluated in the antimicrobial preliminary assay (**Supplementary Table S9**), they were further tested by a disk diffusion method to deeply investigate their antimicrobial activity against the strain of *P. carotovorum* subsp. *carotovorum.* As observed in the preliminary test, extracts and pure compounds showed a mild ability to inhibit the growth of the bacterium. In the disk diffusion test, the extract 26 was active against the strain of *P. carotovorum* subsp. *carotovorum* at concentrations higher than 250 μg/mL; the extracts 1, 7, 16, 20, 22, and 28 at concentrations higher than 500 μg/mL; the extract 6 at concentrations higher than 1,000 μg/mL; and extracts 5, 9, and 32 at concentrations higher than 2,000 μg/mL. Isorosmanol, carnosic acid, and carnosol showed to be active against the strain of *P. carotovorum* subsp. *carotovorum* at concentrations higher than 250 μg/mL, and 12-*O*-methylcarnosic acid and 7-*O*-methylrosmanol at concentrations higher than 1,000 μg/mL (**Supplementary Table S10**). The activity of the tested antibiotic ampicillin against the same strain was several orders of magnitude higher than extracts and pure compounds with a concentration activity value below 1 μg/mL. This result, compared with literature data (Ashmawy et al., 2014; Shaheen and Issa, 2020; Suaza-Gaviria et al., 2023), led to the assessment of a low antimicrobial activity of the selected extracts and pure compounds against the strain of *P. carotovorum* subsp. *carotovorum*.

### 3.5 Enzymatic activity

*In vitro* inhibition of pectinase activity by rosemary diterpenes was investigated using an enzymatic assay. The untreated bacterial suspension of *P. carotovorum* subsp. *carotovorum* at the concentration of 10^7^ CFU/mL showed a pectinase activity of 234.5 U/L corresponding to 234.5 μmol of galacturonic acid hydrolyzed from polygalacturonic acid per minute at 25°C and pH 4 (test conditions). The pectinase activity of the treated bacterial inoculum of *P. carotovorum* subsp. *carotovorum* was 0 U/L for carnosic acid, 20.4 U/L for 12-*O*-methylcarnosic, 0 U/L for 7-*O*-methylrosmanol, 2.15 U/L for carnosol, and 0 U/L for isorosmanol. Carnosic acid, 7-*O*-methylrosmanol, and isorosmanol was shown to be able to inhibit the pectinase activity of bacterial suspension of *P. carotovorum* subsp. *carotovorum* at the concentration of 1,000 ppm. Carnosol and 12-*O*-methylcarnosic applied at the same concentration were shown to partially inhibit the pectinase activity.

### 3.6 Molecular docking

**Figure 6:**
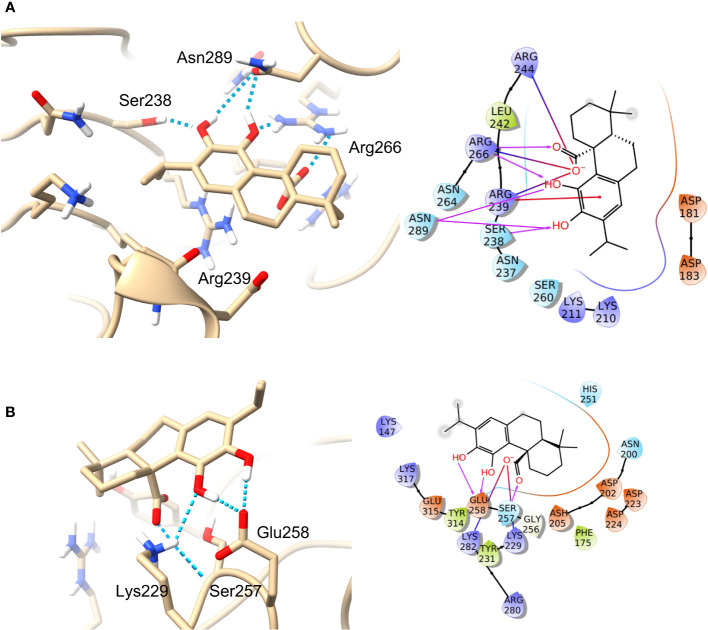
Binding pose and interaction of carnosic acid docked to ligand binding site of *P. carotovorum* pectate lyase 1 (PelA) **(A)** and endo-polygalacturonase (PehA) **(B)**. Left: the protein is reported as light-brown ribbons; the ligand is reported as capped sticks; H-bonds are presented as cyan dotted lines. Right, the ligand is surrounded by the protein residues represented as follows: the negatively charged residues are indicated in red, polar residues are in cyan, hydrophobic residues are shown in green, and H-bonds are depicted as purple arrows.

The predicted molecular interactions of carnosic acid, carnosol, 7-*O*-methyl-rosmanol, 12-*O*-methyl-carnosic acid, and isorosmanol with the binding site residues of *P. carotovorum* pectate lyase 1 (PelA) and *P. carotovorum* endo-polygalacturonase (PehA) were studied. The Ramachandran plot of the *P. carotovorum* pectate lyase 1 (PelA) modeled structure showed that 80.5% of residues were in the most favored region, 19.1% were in additional allowed regions, and 0.3% of residues were in disallowed regions. These results confirmed the high quality of the homology model. The ProSA-web has shown a Z-score of −7.66 that falls in the range of scores commonly found in the case of a similar native protein (**Supplementary Figure S23**). In the *P. carotovorum* pectate lyase 1 active site, all compounds showed the catechol moiety bound with H-bonds (**Supplementary Figure S24**). 12-*O*-Methylcarnosic acid and carnosol showed a common H-bond interaction with Arg239. Carnosic acid, 7-*O*-methylrosmanol, isorosmanol, and 12-*O*-methylcarnosic acid were bound through H-bonds to Arg266 and Asn289 in the active site cleft. The docking poses generated for the selected compounds were similar, except for carnosic acid, for which a “head–tail arrangement” was observed (**Figure 6**). Concerning docking scores, the best result was obtained for carnosol (−4.445 kcal/mol), although no substantial differences to the other compounds were apparent (**Table 3**). In the *P. carotovorum* endo-polygalacturonase (PehA) active site, 7-*O*-methylrosmanol and carnosic acid achieved the best docking score values of −4.766 kcal/mol and −4.377 kcal/mol, respectively (**Table 4**). These two compounds also reported a similar binding pose and two common interactions. 7-*O*-Methylrosmanol was predicted to form two H-bonds with Glu258, one H-bond with Lys229, and one additional H-bond with Asn200. The same interaction pattern with Glu258 and Lys229 was also predicted for carnosic acid (**Figure 6**). Isorosmanol and carnosol showed consistent binding poses, with the catechol moiety bound to Glu315 and Tyr314 (**Supplementary Figure S25**). All the selected compounds showed a hydrophobic interaction with the Phe175 planar ring, reported as an important residue in the binding cleft (Pickersgill et al., 1998).

**Table 3:** Docking interaction parameters of the studied compounds in PelA active site.

Ligand molecules	Glide binding energy (kcal/mol)	GlideScore SP (kcal/mol)	Docking score SP	H-bond interacting amino acids	Hydrophobic interactions	Salt bridges
carnosic acid	-24.744	-3.573	-3.570	Ser238, Arg266, Asn289	Arg239, Asn289	Arg239, Arg244, Arg266
carnosol	-28.934	-4.445	-4.445	Asp183, Asn237, Ser238, Arg239	Leu242	–
7-*O*-methyrosmanol	-34.297	-3.987	-3.987	Asn237, Ser238, Asn264, Arg266, Asn289	Asn289	Lys210
isorosmanol	-27.147	-3.756	-3.756	Asp183, Arg244, Arg266, Asn289	Lys211, Arg239	–
12-*O*-methylcarnosic acid	-25.654	-3.495	-3.492	Ser238, Arg239, Arg244, Arg266, Asn289	Asn289	–

**Table 4:** Docking interaction parameters of the studied compounds in PehA active site.

Ligand molecules	Glide binding energy (kcal/mol)	GlideScore SP (kcal/mol)	Docking score SP	H-bond interacting amino acids	Hydrophobic nteractions	Salt bridges
carnosic acid	-31.530	-4.379	-4.377	Lys229, Ser257, Glu258	Phe175	Lys229, Lys282
carnosol	-29.116	-3.744	-3.744	Ser257, Tyr314, Glu315	Phe175	Lys229, Lys282
7-*O*-methylrosmanol	-34.093	-4.766	-4.766	Asn200, Lys229, Glu258	Phe175, Tyr314	Lys229, Lys282
isorosmanol	-33.217	-4.203	-4.202	Asn200, Asp223, Lys229, Ser257, Lys282, Tyr314, Glu315	Phe175	Lys229, Lys282
12-*O*-methylcarnosic acid	-26.724	-3.986	-3.983	Gln148, Asn200	Phe175	–

## 4 Resource identification initiative

Chenomx NMR Suite 8.6 (Chenomx Inc., Edmonton, 252 Canada) was used to identify the metabolites. NMRProcFlow 1.4.14 (INRA UMR 1332 BFP, Bordeaux Metabolomics Facility, Villenave d’Ornon, France) was used for quantitative analysis of NMR spectra. Systat software for Windows Version 13 (Systat Software Inc., Chicago, IL, USA) and Matlab R2022a and SOM toolbox 2.1 (The MathWorks, Inc., Natick, MA, USA) were used for multivariate data analysis. Schrödinger Suite Schrödinger Release 2020-4: Maestro version 12.6.144, Glide, Ligprep, Prime (Schrödinger, LLC: New York, NY, 2021) was used for *in silico* studies.

## 5 Discussion

Plant bio-residues were identified as an excellent source of high-added value products potentially useful as beneficial food constituents and supplements, cosmetics, chemopreventive agents, and drug adjuvants. Hence, it is of growing interest to develop sustainable strategy to fully valorize agri-food by-products to obtain bioactive components and preparations (Lemes et al., 2021). The aim of the present study was to characterize the qualitative profile of a local rosemary ecotype for comparison with other cultivars and ecotypes and to identify new possible applications in the agro-food chain. In the current study, targeted NMR-based metabolite analysis was used to study local rosemary accessions compared with commercial cultivars grown in both the same and different climatic conditions and geographical areas. All samples were collected in summer when the content of abietane diterpenoids is at its maximum (Hidalgo et al., 1998). Unsupervised techniques, i.e., principal component analysis (PCA), clustering, and self-organizing maps (SOMs), were applied (Corsaro et al., 2022). Multivariate analysis showed that accessions from the Campania region differed from the Ligurian ones, thus leading to the hypothesis of the influence of geographical location in the composition and abundance of metabolites in rosemary extracts. The evident differences in the metabolite profiles of the samples collected in the two Italian regions (Liguria and Campania) seemed to confirm the relevance of the local environmental conditions in the characterization of the rosemary samples. Carnosic acid characterized northern accessions, consistent with the fact that in drought conditions typical of Southern Italy, it undergoes oxidation to other abietane diterpenes such as isorosmanol and other related compounds (Munné-Bosch et al., 2000; Zhang et al., 2012). However, genetic factors became more important when a more restricted geographical area was considered, such as the Ligurian region. This result is in accordance with the literature (Hidalgo et al., 1998) and for volatile constituents of rosemary essential oil (Li et al., 2016), where genetic and origin are the key factors in chemotype variation. The accessions of ‘Eretto Liguria’ analyzed in the present study, even though they were collected in different locations of the same region (i.e., coastal and inland, different producers, different soils, etc.), appeared well characterized, while accessions of other ecotypes/cultivars, although grown in the same location in the same conditions, were not so similar to each other. The concentrations of carnosic acid, carnosol, and rosmarinic acid were considered decisive for the ranking of rosemary accessions (Wellwood and Cole, 2004). In our study, maps of CCL compounds (**Supplementary Figures S19, S20**) showed that the most abundant abietane diterpenes in Ligurian rosemary extracts (**Supplementary Table S1**) were 12-*O*-methylcarnosic acid (CCL1), 7-*O*-methylrosmanol (CCL2), carnosic acid (CCL7), carnosol (CCL12), and isorosmanol (CCL23). 12-*O*-Methylcarnosic acid (CCL1) characterized ‘Benenden Blue’ (21, 22), ‘Prostratus’ (Prostrata Group) (9), ‘Joyce DeBaggio’ (18), ‘Majorca Pink’ (25), ‘Porto Alabe’ (27 and 28), and ‘Santa Barbara Blue’ (30, 31, and 32). 7-*O*-methylrosmanol (CCL2) characterized ‘Eretto Liguria’ (12), ‘Prostratus’ (Prostrata Group) (9), 18 ‘Joyce DeBaggio’ (18), and ‘Santa Barbara Blue’ (32). Higher amounts of carnosic acid (CCL7) were shown by ‘Eretto Liguria’ (1, 2, 4, 12, 13, and 14), ‘Eretto’ (local ecotype) (7), ‘Prostratus’ (Prostrata Group) (6 and 11), and ‘Boule’ (Prostrata Group, ‘Rampant Boule’) (17). Carnosol (C12) characterized ‘Eretto’ (local ecotype) (7), ‘Prostratus’ (Prostrata Group) (9), ‘Joyce DeBaggio’ (18, 19), ‘Santa Barbara Blue’ (31 and 32), ‘Benenden Blue’ (22), and ‘Porto Alabe’ (28). Isorosmanol (CCL23) characterized ‘Prostratus’ (Prostrata Group) (9), ‘Joyce DeBaggio’ (18), and ‘Santa Barbara Blue’ (32). Epiisorosmanol (CCL27) was widespread among accessions but in very small amount. Abietane diterpenoids were distributed among the Ligurian accessions with no relatively different quantities. The ‘Eretto Liguria’ ecotype appeared well characterized by a high carnosic acid content, which can be considered a good source of abietane diterpenes.

Abietane diterpenoids are reported as potent antimicrobials and in phytopatological defense (Birtić et al., 2015; González, 2015; Bellumori et al., 2021). In the present work, the activity against bacterial soft rot induced by *P. carotovorum* subsp. *carotovorum* of the methanolic extracts of the rosemary accessions, and of selected abietane diterpenoids, was tested. *P. carotovorum* subsp. *carotovorum* causes severe soft rots in the field and in storage, leading to extensive harvest losses. In the field, *P. carotovorum* subsp. *carotovorum* can survive in the soil for up to 6 months, even in the absence of plant debris, and it can also be present in aerosols and water irrigation. Wet conditions increase the effects of its spreading; therefore, some seasons have a high level of damage (OEPP/EPPO, 1994). In addition, airborne insects can carry bacteria from one plant to another (Rossmann et al., 2018). The most distinguishing feature of the pathogenicity of soft rot bacteria as *P. carotovorum* subsp. c*arotovorum* produces several plant cell-wall-degrading enzymes, such as pectinases, cellulases, and proteases, leading to tissue decomposition and the release of nutrients for bacterial growth (Pritchard et al., 2016). The bacterium enters potato tubers through lenticels and fresh wounds, roots, and above-ground plant parts. Tubers harvested from plants infected during the growing season may develop a soft rot in storage, which results in considerable postharvest losses (OEPP/EPPO, 1994; Hadizadeh et al., 2019). Currently, the control of soft rot pathogens relies on integrated pest management because no chemical treatments, such as synthetic bactericides, are currently recommended to control this disease (Mantsebo et al., 2014). Due to the ease of developing soft rot rapidly, the potato soft rot test is usually performed to define the ability of an inoculum to induce pectolytic activity (Lelliott et al., 1966). In our experiment, potato tuber assay was used to test the methanolic extracts’ ability to reduce the soft rot induced by a strain of *P. carotovorum* subsp. c*arotovorum* applied on potato tissue after treatment and measuring tissue maceration. The best results in the inhibition of bacterial soft rot were attributable to ‘Eretto Liguria’ (1), ‘Prostratus’ (Prostrata Group) (6), ‘Boule’ (Prostrata Group, ‘Rampant Boule’) (16), ‘Joyce DeBaggio’ (20), ‘Benenden Blue’ (22) ‘Majorca Pink’ (26), ‘Porto Alabe’ (28), and ‘Santa Barbara Blue’ (32). The inhibition induced by various extracts appeared then not linked to the distribution of the different accessions in the clusters (**Supplementary Figures S17, S18, S20, S21**), thus suggesting that the differences in relative abundance of individual compounds within the phytocomplex are almost negligible. The bacterial soft rot assay performed with the pure compounds showed the best results for 7-*O*-methylrosmanol, carnosol and isorosmanol, while 12-*O*-methylcarnosic acid and carnosic acid were less active. The low inhibition activity of carnosic acid is probably related to the phytotoxicity effect observed *in vivo* when the compound is applied at concentration higher than 500 ppm (unpublished data). The mild antimicrobial activity showed by the extracts and pure compounds in the disk diffusion test suggested that the ability in inhibiting the progression of bacterial soft rot on potato tissue could be only partially affected by the activity on the bacterial growth, and different mechanisms could probably lead it.

Based on these results and considering the relevant role of the pectinase enzymes in the soft rot infections and the increasing interest in their biotechnological application, a computational approach was employed to derive the potential binding mode of the abietane diterpenoids to selected pectinase enzymes. Pathogens secrete plant cell-wall-degrading enzymes as virulence factors. Pectinases are mainly produced by plant pathogens, and they are involved in plant cell wall degradation (Davidsson et al., 2013). Pectic enzymes are considered a central factor in plant tissue maceration during soft rot infection (Abbott and Boraston, 2008). Pectinases are classified into three major groups according to the substrate and the mechanism of action. *Pectobacterium* spp. produces polygalacturonase (PG, EC 3.2.1.15), polygalacturonate lyase (PGL, EC 4.2.2.2), and oligogalacturonide lyase (OGL, EC 4.2.2.6) (Sakai et al., 1993). Pectin degradation by endopolygalacturonase lyase (PGL), also called pectic transeliminase, involves a β-elimination mechanism and a basic residue acting as a Brønsted base to abstract the proton at the C-5 position of the galacturonate residue (Hugouvieux-Cotte-Pattat et al., 2014). These extra cellular enzymes are implicated in the degradation of plant tissue, in particular in the cleavage of (1-4)-α-D-galacturonan to give oligosaccharides with 4-deoxy-α-D-galact-4-enuronosyl groups at their non-reducing ends (Collmer and Keen, 1986; Marín-Rodríguez et al., 2002). At present, pectate lyases are under investigation for their biotechnological and industrial applications (Wu et al., 2020). Most of the polygalacturonate lyase activity results from the cluster activity of five major isoenzymes, PelA to PelE, which were differentiated by an increasing pI from PelA to PelE (Collmer and Keen, 1986). A more alkaline pH than the hydrolytic enzymes and divalent cations were found to be essentials for the activity of pectate lyases (Tardy et al., 1997). PelA has been defined as critical for developing symptoms (Barras et al., 1994), and its production could be the first step of an infectious process (Nachin and Barras, 2000). The peculiar role of this isoenzyme in the infection process has been underlined (Beaulieu and Van Gijsegem, 1993). Moreover, the optimum pH for its enzymatic activity is reported to be 8–8.5 (Hugouvieux-Cotte-Pattat et al., 2014). Endo-polygalacturonase (endoPG), also known as pectic hydrolase, cleaves the α-(1–4) linkages between D-galacturonic acid residues of homogalacturonan, the main component of pectin, by hydrolysis (Sakai et al., 1993). The strict similarity in the whole electrostatic properties of polygalacturonase and polygalacturonate lyase (pectate lyase) substrate-binding clefts, which bind and cleave the same substrate, polygalacturonic acid, has been revealed (Pickersgill et al., 1998).

Among the several enzymes produced by *P. carotovorum*, only the crystal structure of endo-polygalacturonase (PehA) from *P. carotovorum* (Pickersgill et al., 1998) has been reported in the Protein Data Bank (Berman et al., 2000). Moreover, since the three-dimensional structure of the *P. carotovorum* pectate lyase 1 (PelA) has not yet been solved, a new homology model of PelA was built using the crystal structure of pectate lyase C (Scavetta et al., 1999) as the template, due to the high amount of sequence similarity. A detailed analysis of the active site revealed a high degree of conservation of the residues of the ligand binding site of the family 1 Pels (PelA) and PelC (PDB ID, 2EWE) (Abbott and Boraston, 2008). The docking simulations suggested that the selected compounds may interact with both modeled PelA and PehA binding sites. As expected, no substantial differences in docking scores were observed given the structural similarity between the compounds. The hypothetical bond of the abietane diterpenoids at the PelA and PehA active sites may prevent the pectolytic reactions on potato tubers. Further studies of the pectinase enzymes 3D-structures will be needed to derive the pectolytic activity inhibition exerted by these natural compounds. The ability to inhibit the pectinase activity by the tested compounds was then assayed *in vitro*. Quantitative pectinase activity determination confirmed that carnosic acid, 7-*O*-methylrosmanol, and isorosmanol can inhibit the pectinase activity of bacterial suspension of *P. carotovorum* subsp. *carotovorum*. The obtained results suggest that treatments with methanolic extracts of rosemary can also reduce the severity of soft rot disease during storage, and these extracts could be further studied as an alternative strategy for bacterial soft rot management. The NMR-based metabolomic software used in the present study required a specific sample preparation based on the use of deuterated methanol. Therefore, the methanolic extracts for antimicrobial and bacterial soft rot assay were prepared. Additional research is needed involving less-impacting binary solvent mixtures (e.g., water and organic solvent) (Plaskova and Mlcek, 2023). Based on these results, a further study will provide various bacterial strains acting on the pectinase enzymes. Moreover, considering the possible phytopathological application, the use of dried powder material could be considered.

## 6 Conclusion

Targeted NMR-based metabolite analysis was employed to analyze local rosemary accessions compared with commercial cultivars grown in the same/or in different climatic conditions and geographical areas. Unsupervised techniques were applied, i.e., PCA, clustering, and SOMs (Corsaro et al., 2022). Multivariate data analysis showed that accessions from the Campania region differed from the Ligurian ones, thus leading to the hypothesis of the influence of geographical location in the composition and abundance of metabolites in rosemary extracts. Moreover, cluster analysis reported the accessions of ‘Eretto Liguria’ to be well characterized among the other Ligurian cultivars and ecotypes, showing high content of carnosic acid. The methanolic extracts of the rosemary accessions and all the selected abietane diterpenoids exhibited activity against soft rot induced by *P. carotovorum* subsp. *carotovorum* on potato tuber slices. ‘Eretto Liguria’ achieved one of the best results in inhibiting bacterial soft rot. Isorosmanol, carnosol, and 7-*O*-methylrosmanol completely inhibited the bacterial soft rot. In addition, the *in silico* study suggested possible molecular interactions of the selected abietane diterpenoids with *P. carotovorum* subsp. *carotovorum* pectolytic enzymes. Finally, the *in vitro* enzymatic assay confirmed the possibility of inhibition of pectinase activity. The possible use of rosemary methanolic extracts and pure abietane diterpenoids in preventing and reducing the soft rot disease strictness has been underlined.

## Data availability statement

The raw data supporting the conclusions of this article will be made available by the authors, without undue reservation.

## Author contributions

VI, VP, and GDo performed phytochemical investigations. VI performed the metabolite identification. VI, NM, MG, and GDr performed the multivariate analysis. AL and GM performed the antibacterial investigation. VI and JK performed the *in silico* study. AB and ND performed the conception and design of the study and wrote sections of the manuscript. All authors contributed to the article and approved the submitted version.
