# Supplementary material for: Targeted metabolite profiling of Salvia rosmarinus Italian local ecotypes and cultivars and inhibitory activity against Pectobacterium carotovorum subsp. carotovorum
Source: Front Plant Sci. 2024 Feb 2;15:1164859. doi: 10.3389/fpls.2024.1164859 (PMC10883066; doi:10.3389/fpls.2024.1164859)
Supplement: Supplementary file 1 [file DataSheet_1.pdf]

Targeted metabolite profiling of *Salvia rosmarinus* Italian local ecotypes and cultivars and inhibitory activity against *Pectobacterium carotovorum* subsp. *carotovorum*

Valeria Iobbi[1⊥], Giuliana Donadio[2⊥], Anna Paola Lanteri[3], Norbert Maggi[4], Johannes Kirchmair[5], Valentina Parisi[2#], Giovanni Minuto[3], Andrea Copetta[6], Mauro Giacomini[5], Angela Bisio[1*], Nunziatina De Tommasi[2*], Giuliana Drava[1]

[1] Department of Pharmacy, University of Genova, Genova, Italy
[2] Department of Pharmacy, University of Salerno, Fisciano, Italy
[3] Centro di Sperimentazione e Assistenza Agricola (CeRSAA), Albenga, Italy
[4] Department of Informatics, Bioengineering, Robotics and System Science, University of Genova, Genova, Italy
[5] Department of Pharmaceutical Sciences, Division of Pharmaceutical Chemistry, University of Vienna, Vienna, Austria
[6] Centro di Ricerca Orticoltura e Florovivaismo (CREA), Sanremo, Italy
[#]PhD Program in Drug Discovery and Development, Department of Pharmacy, University of Salerno, Fisciano, Italy.

[⊥]These authors have contributed equally to this work and share first authorship

*** Correspondence:** Angela Bisio angela.bisio@unige.it; Nunziatina De Tommasi detommasi@unisa.it

Supplementary Material

1 Supplementary Figures and Tables

1.1 Supplementary Figures

Figure S1. Representative ^{1}H-NMR spectrum of *S. rosmarinus* (accession 2, Table S1) leaves extract in CD_3OD-KH_2PO_4 in D_2O at pH 6.0, 500 MHz.
Figure S2. Representative HSQC spectrum of *S. rosmarinus* (accession 2, Table S1) leaves extract in CD_3OD-KH_2PO_4 in D_2O at pH 6.0.
Figure S3. Representative HMBC spectrum of *S. rosmarinus* (accession 2, Table S1) leaves extract in CD_3OD-KH_2PO_4 in D_2O at pH 6.0.
Figure S4. Representative COSY spectrum of *S. rosmarinus* (accession 2, Table S1) leaves extract in CD_3OD-KH_2PO_4 in D_2O at pH 6.0.
Figure S5. ^{1}H-NMR spectra.
Figure S6. Similarity dendrograms obtained from cluster analysis (single linkage method based on Pearson coefficients) applied.
Figure S7. Similarity dendrogram obtained from cluster analysis (single linkage method based on Pearson coefficients) applied to the 38 metabolites (Chenomx 500 MHz custom library metabolites (CCL) and Chenomx 500 MHz version 11 library metabolites (CL) jointly) measured on 111 rosemary samples.
Figure S8. Similarity dendrogram of the 111 rosemary samples obtained by cluster analysis (single linkage method based on Euclidean distance) based on the 11 Chenomx 500 MHz version 11 library metabolites (CL).
Figure S9. Similarity dendrogram of the rosemary accessions (111 samples) obtained by cluster analysis (single linkage method based on Euclidean distance) based on the 27 Chenomx 500 MHz custom library metabolites (CCL).
Figure S10. Similarity dendrogram of the 111 rosemary samples obtained by cluster analysis (single linkage method based on Euclidean distance) based on the 38 metabolites (Chenomx 500 MHz custom library metabolites (CCL) and Chenomx 500 MHz version 11 library metabolites (CL) jointly).

Figure S11. Results of PCA of Chenomx 500 MHz version 11 library metabolites (CL) measured in 111 samples: biplot of Principal Components 1 and 2 (52% of explained variance).
Figure S12. Results of PCA of Chenomx 500 MHz version 11 library metabolites (CL) measured in 111 samples: biplot of Principal Components 3 and 4 (29% of explained variance).
Figure S13. Results of PCA of Chenomx 500 MHz custom library metabolites (CCL) measured in 111 samples: biplot of Principal Components 1 and 2 (51% of explained variance).
Figure S14. Results of PCA of Chenomx 500 MHz custom library (CCL) metabolites measured in 111 samples: biplot of Principal Components 3 and 4 (17% of explained variance).
Figure S15. Results of PCA of Chenomx 500 MHz custom library metabolites (CCL) measured in 111 samples: biplot of Principal Components 5 and 6 (14% of explained variance).
Figure S16. SOM, U-matrix and maps for each Chenomx 500 MHz version 11 library metabolite (CL). Similar color gradations indicate highly correlated variables.
Figure S17. SOM: Graphical representation of map for Chenomx 500 MHz version 11 library metabolites (CL).
Figure S18. SOM: Map clusterization for Chenomx 500 MHz version 11 library metabolites (CL).
Figure S19. U-matrix and maps for each Chenomx 500 MHz custom library (CCL) metabolite.
Figure S20. Graphical representation of Chenomx 500 MHz custom library (CCL) metabolites on map.
Figure S21. Map clusterization.
Figure S22. Bacterial soft rot assay (a) and graphs of the results (b, c): potato tuber slices inoculated with the bacterial suspension of *P. carotovorum* subsp. *carotovorum* after 5 days of incubation at 25 °C.
Figure S23. ProSA z-score (left) and Ramachandran plot (right) of the *P. carotovorum* pectate lyase 1 (PelA) homology model.
Figure S24. Binding pose (left) and interaction (right) of the selected compounds docked to ligand binding site of *P. carotovorum* pectate lyase 1 (PelA).
Figure S25. Binding pose (left) and interaction (right) of the selected compounds docked to ligand binding site of *P. carotovorum* endo-polygalacturonase (PehA).

1.2 Supplementary Tables

Table S1. List of rosemary accessions used in the study.
Table S2. Rosemary accessions data.
Table S3. Brief description of the commercial cultivars used in the study.
Table S4. Morphological traits of the ecotype "Eretto Liguria".
Table S5. ^{1}H NMR chemical shifts (δ) and coupling constants (Hz) of Chenomx 500 MHz custom library metabolites (CCL) identified by using 1D spectra in CD_3OD-KH_2PO_4 in D_2O at pH 6.0, 500 MHz.
Table S6. Selected ^{1}H NMR chemical shifts (δ) and coupling constants (Hz) of Chenomx 500 MHz version 11 library metabolites (CL).
Table S7. Pearson correlation coefficients among 500 MHz version 11 Chenomx library metabolites (CL). [a]
Table S8. Pearson correlation coefficients among custom 500 MHz version 11 Chenomx library (CCL) metabolites. [a]
Table S9. Quantification in CFU/mL of *Pectobacterium carotovorum* subsp. *carotovorum* after the exposure to the extracts / pure compounds at 1000 ppm, 500 ppm, 50 ppm and 0 ppm, respectively.
Table S10. Disk diffusion test.

Supplementary Figures

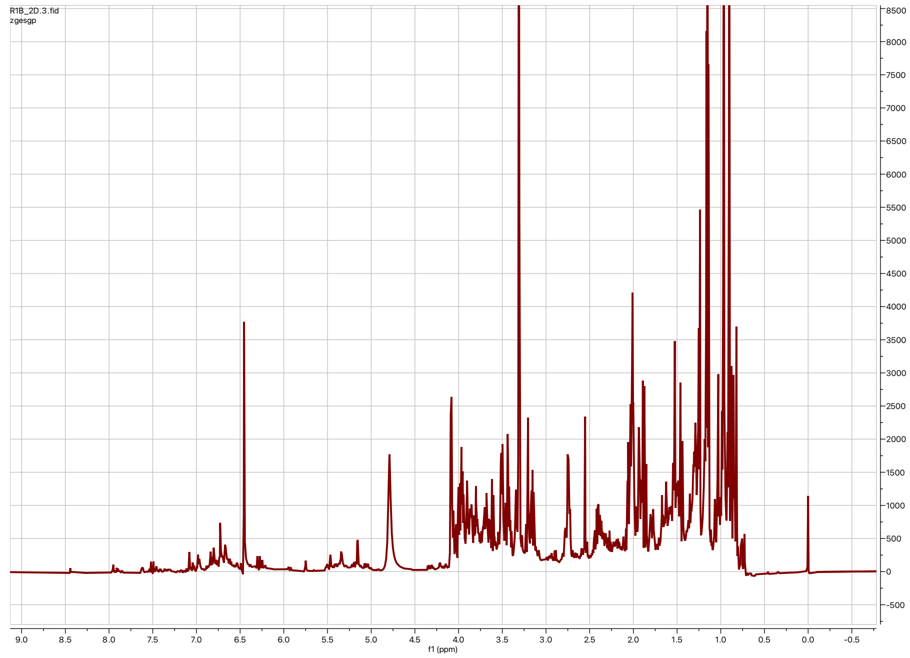

Figure S1. Representative ^{1}H-NMR spectrum of *S. rosmarinus* (accession 2, Table S1) leaves extract in CD_3OD-KH_2PO_4 in D_2O at pH 6.0, 500 MHz.

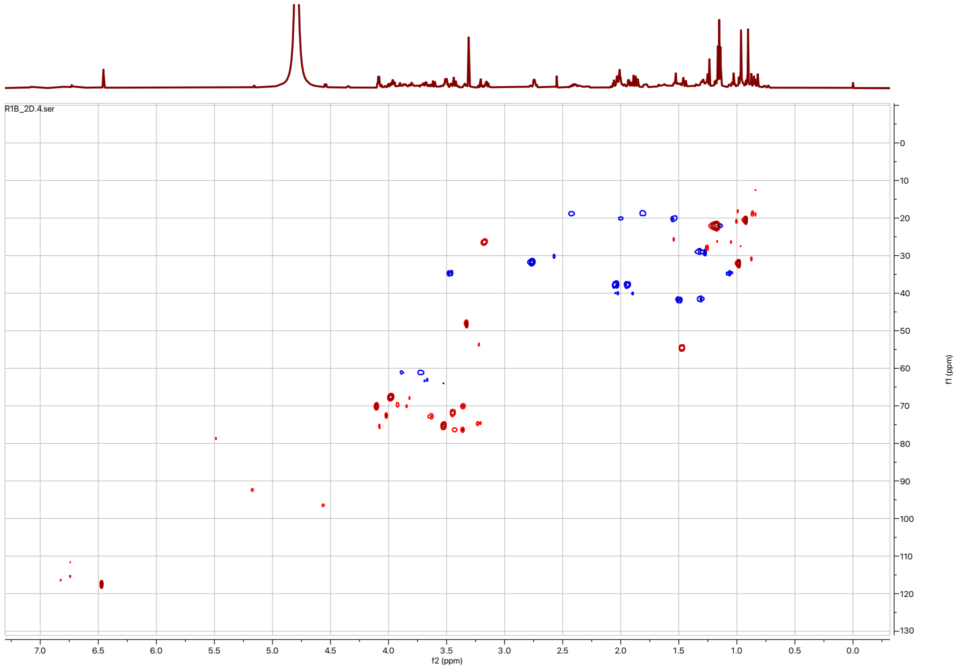

Figure S2. Representative HSQC spectrum of *S. rosmarinus* (accession 2, Table S1) leaves extract in CD_3OD-KH_2PO_4 in D_2O at pH 6.0.

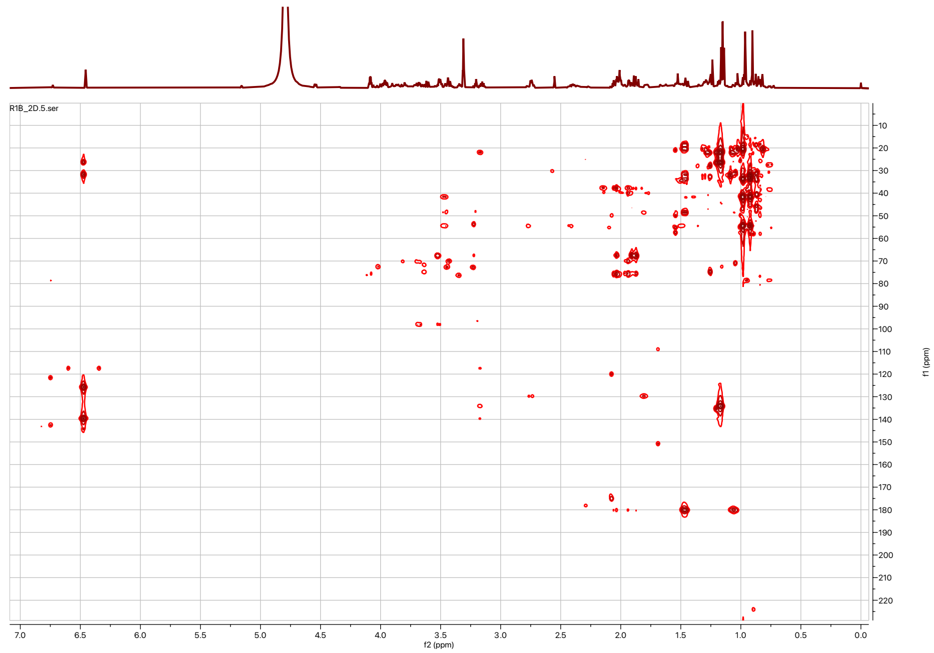

Figure S3. Representative HMBC spectrum of *S. rosmarinus* (accession 2, Table S1) leaves extract in CD_3OD-KH_2PO_4 in D_2O at pH 6.0.

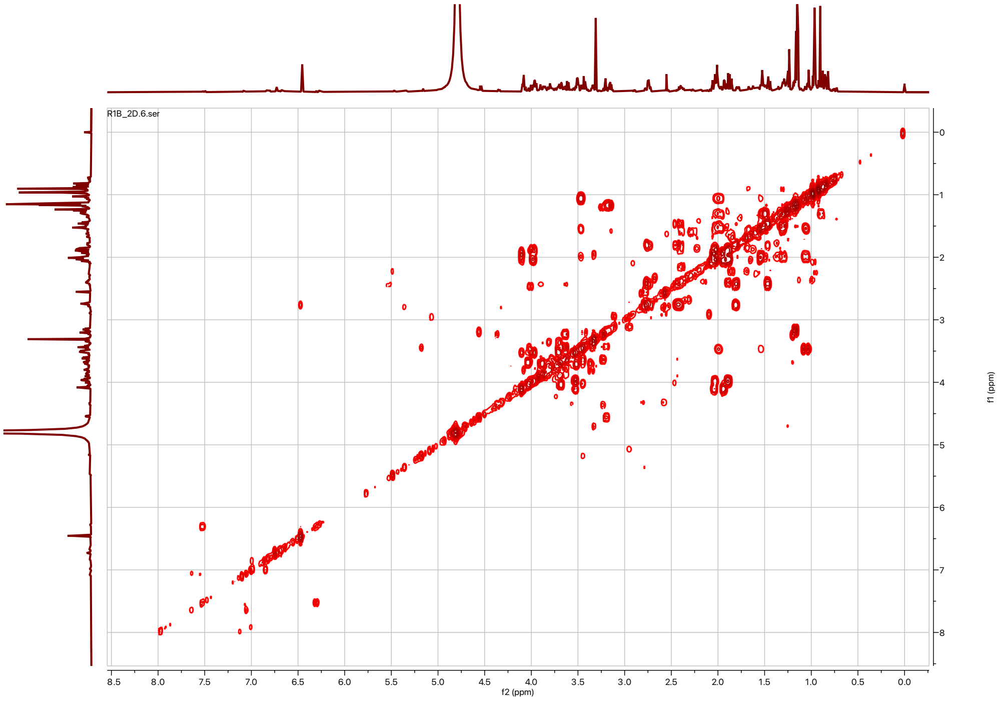

Figure S4. Representative COSY spectrum of *S. rosmarinus* (accession 2, Table S1) leaves extract in CD_3OD-KH_2PO_4 in D_2O at pH 6.0.

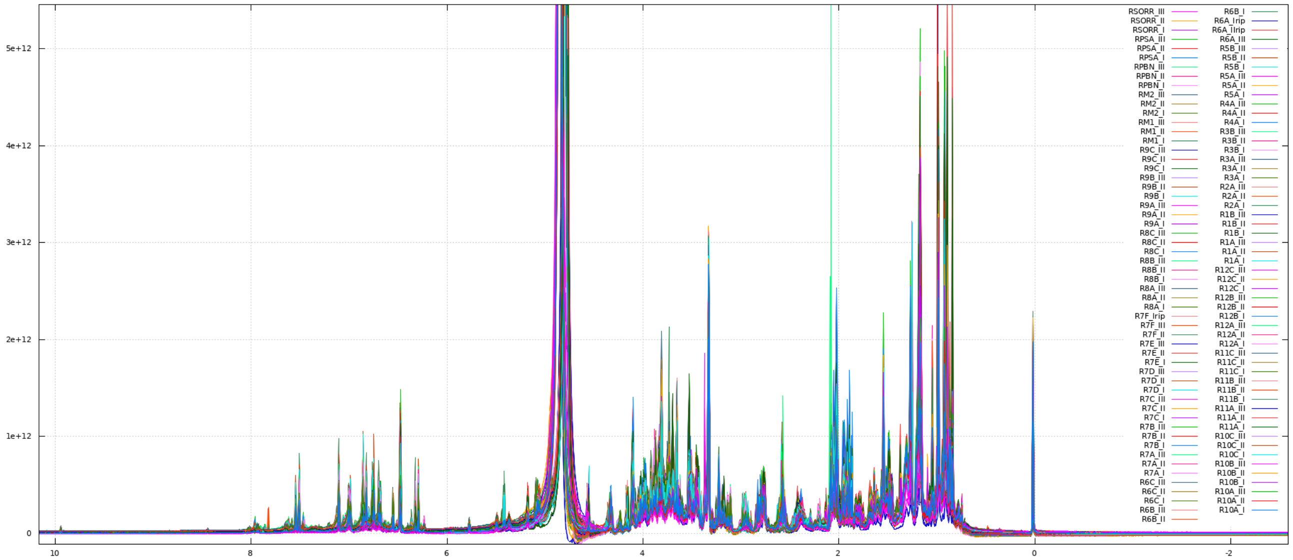

1

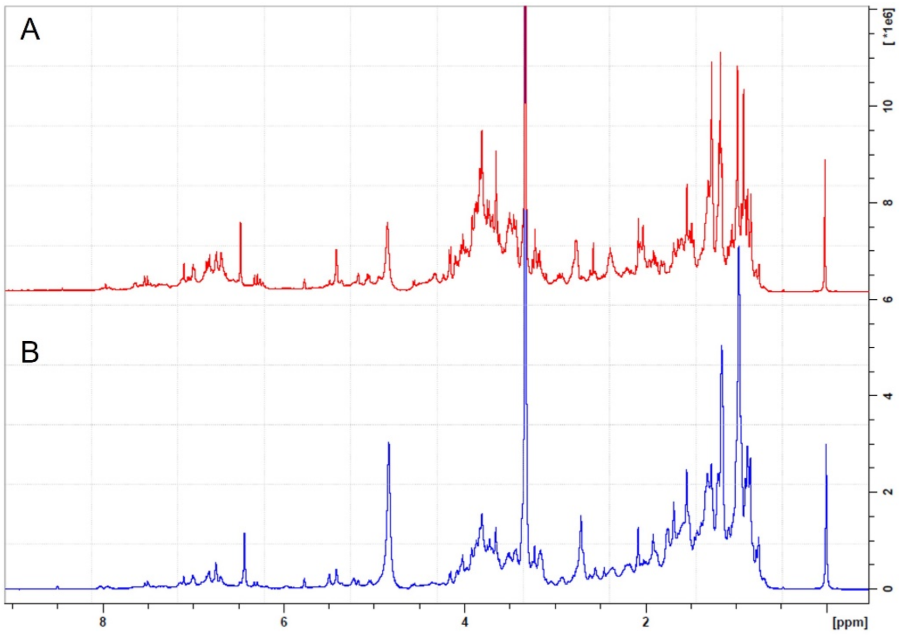

2

Figure S5. ^{1}H-NMR spectra.
1: spectra of the rosemary extracts (direct extraction) using the open access software NMRProcFlow v1.4.14. Spectra of the extracts are presented in different colors.
2: comparison between an NMR spectrum of the direct extraction for the NMR experiment with CD_3OD 0.01% 3-(trimethylsilyl)propionic-2,2,3,3-d_4 acid sodium salt (TSP)/ D_2O 90 mM H_2KPO_4 buffer 6.5:2.5 (A) and NMR spectrum (CD_3OD) of the methanolic extract for the soft rot assay (B).

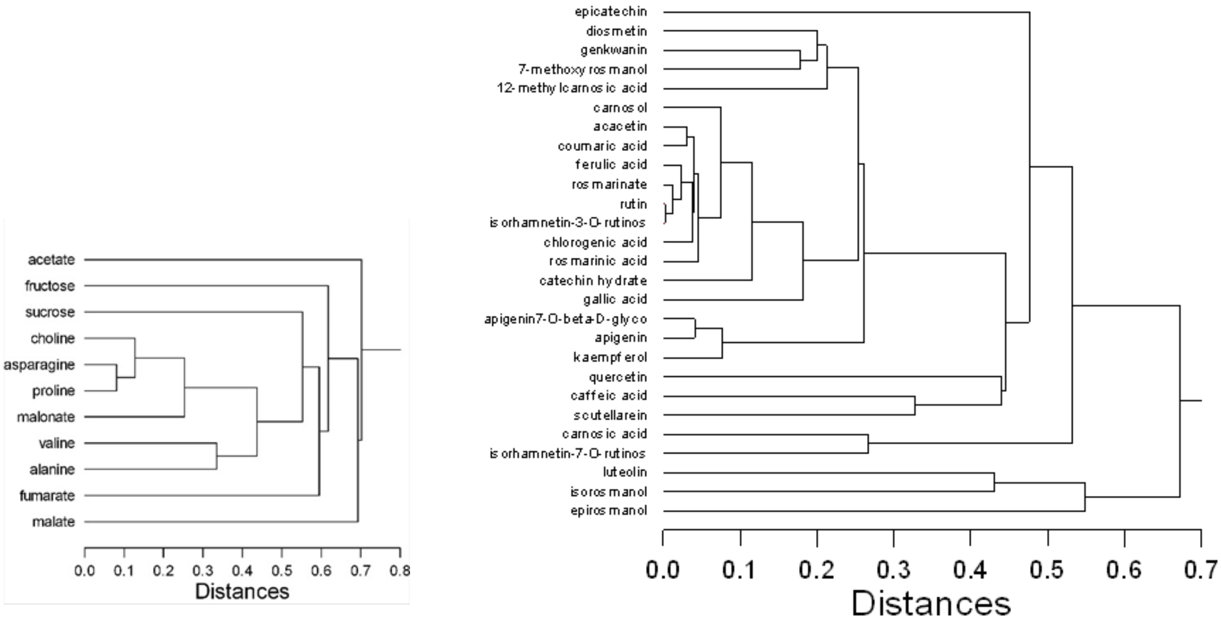

A **B**

Figure S6. Similarity dendrograms obtained from cluster analysis (single linkage method based on Pearson coefficients) applied.
A) to the 11 metabolites of the Chenomx 500 MHz version 11 library (CL); B) to the 27 metabolites of the Chenomx 500 MHz custom library (CCL) measured on 111 rosemary samples.

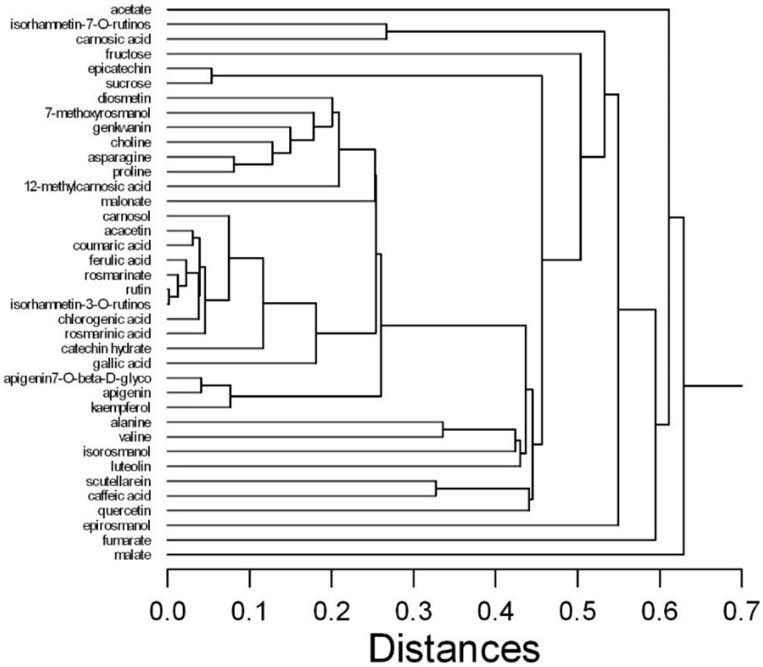

Figure S7. Similarity dendrogram obtained from cluster analysis (single linkage method based on Pearson coefficients) applied to the 38 metabolites (Chenomx 500 MHz custom library metabolites (CCL) and Chenomx 500 MHz version 11 library metabolites (CL) jointly) measured on 111 rosemary samples.

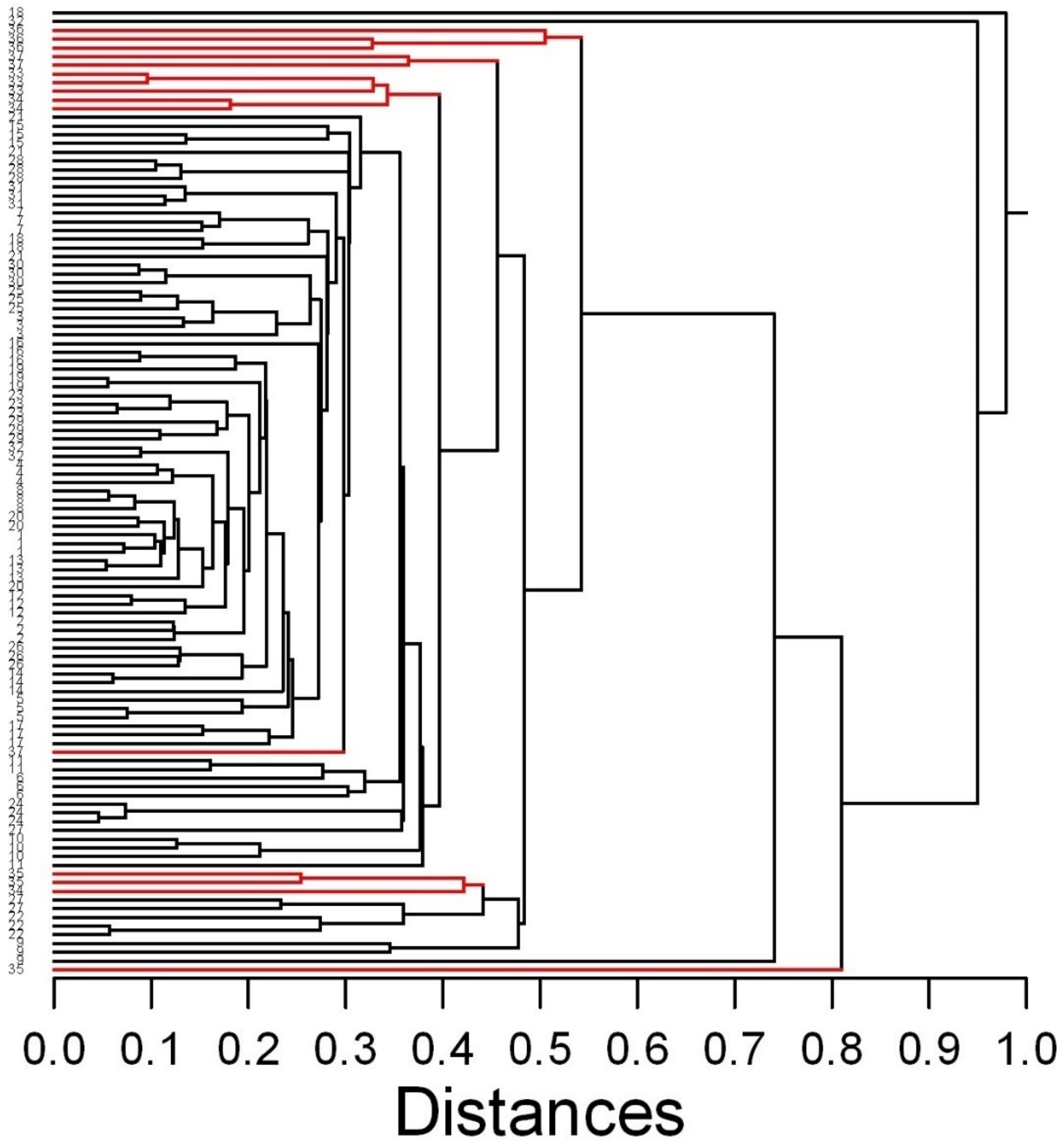

Figure S8. Similarity dendrogram of the 111 rosemary samples obtained by cluster analysis (single linkage method based on Euclidean distance) based on the 11 Chenomx 500 MHz version 11 library metabolites (CL). black line = Liguria; red line = Campania

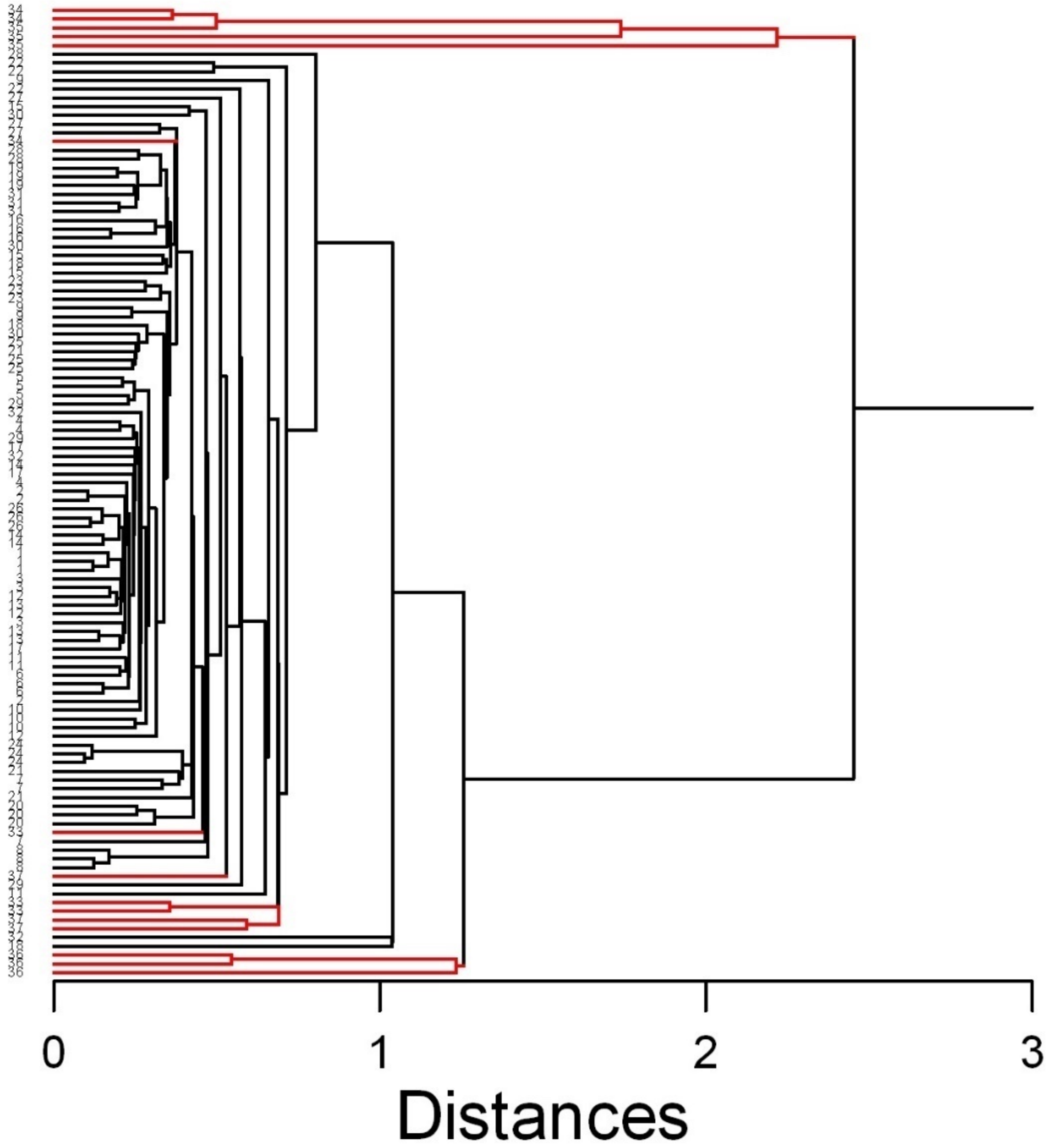

Figure S9. Similarity dendrogram of the rosemary accessions (111 samples) obtained by cluster analysis (single linkage method based on Euclidean distance) based on the 27 Chenomx 500 MHz custom library metabolites (CCL).
black line = Liguria; red line = Campania

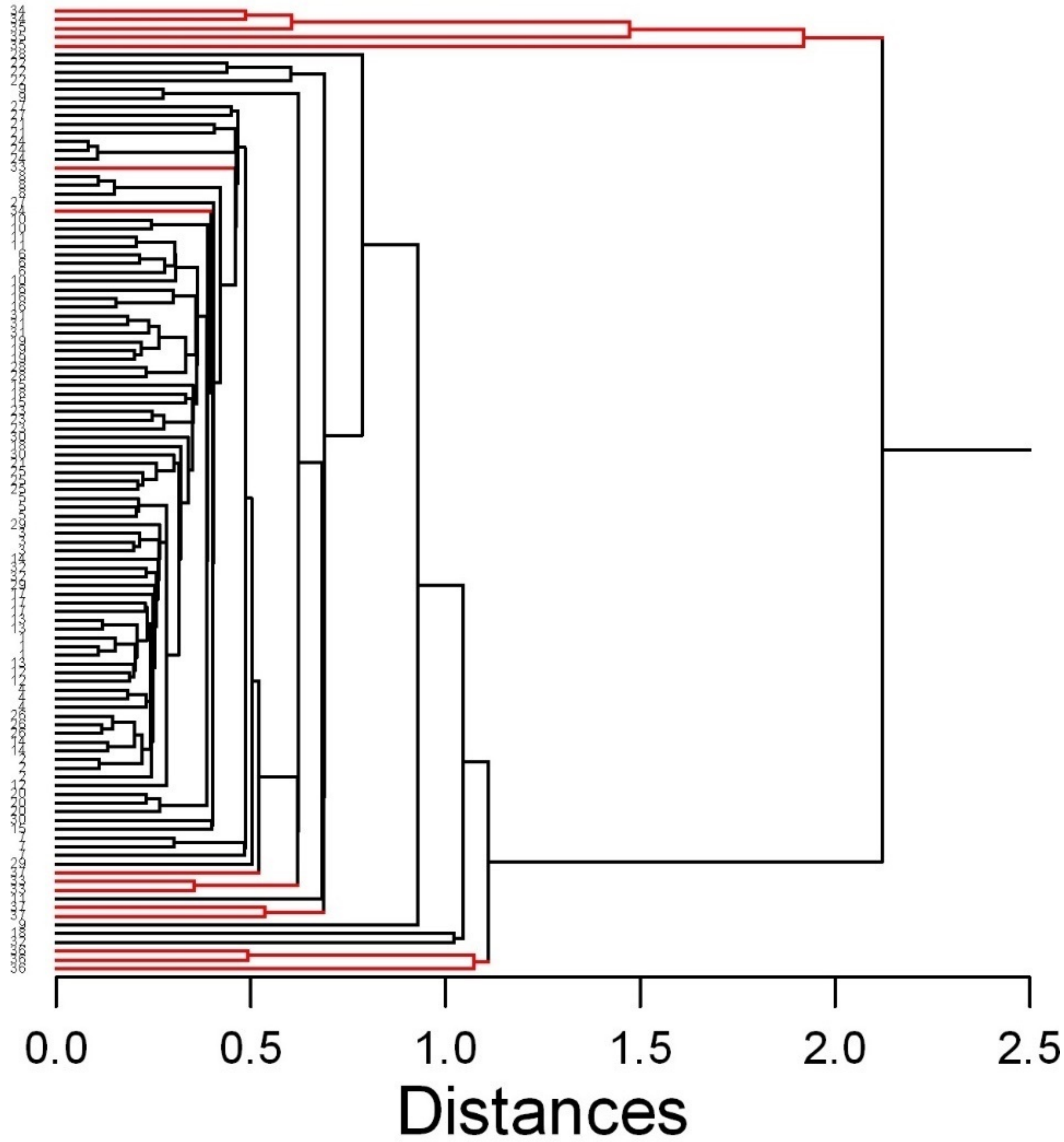

Figure S10. Similarity dendrogram of the 111 rosemary samples obtained by cluster analysis (single linkage method based on Euclidean distance) based on the 38 metabolites (Chenomx 500 MHz custom library metabolites (CCL) and Chenomx 500 MHz version 11 library metabolites (CL) jointly).
black line = Liguria; red line = Campania

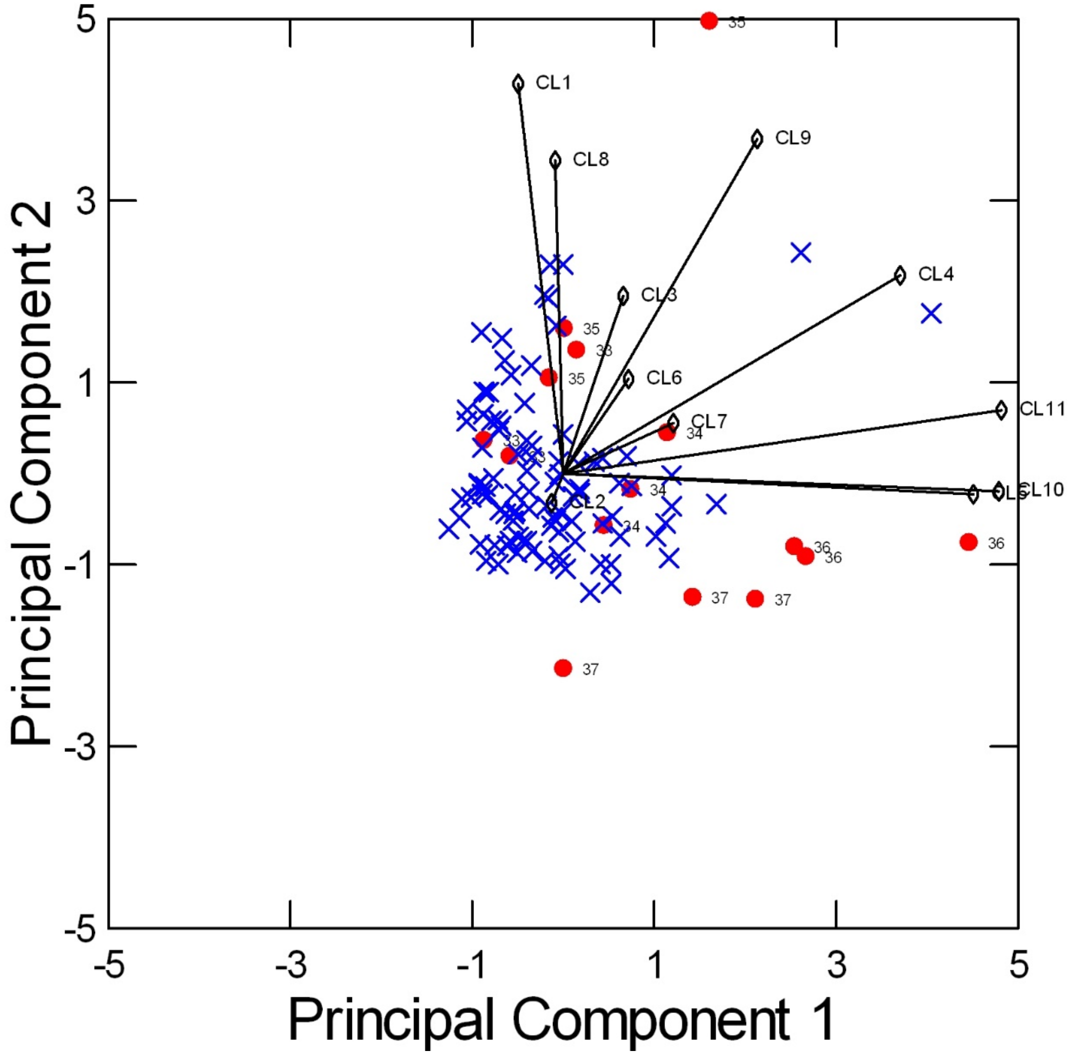

Figure S11. Results of PCA of Chenomx 500 MHz version 11 library metabolites (CL) measured in 111 samples: biplot of Principal Components 1 and 2 (52% of explained variance).
× = Liguria; • = Campania

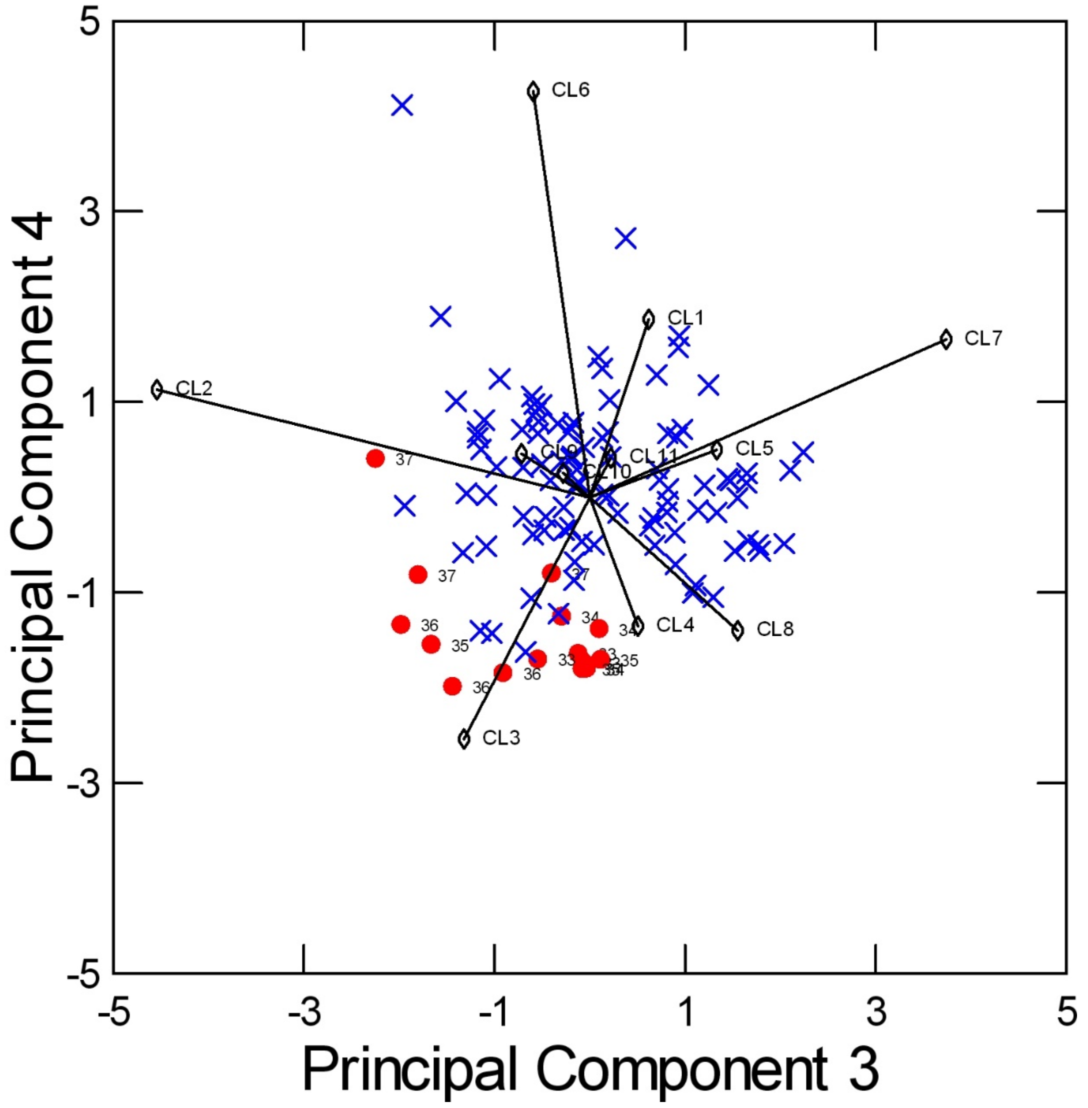

Figure S12. Results of PCA of Chenomx 500 MHz version 11 library metabolites (CL) measured in 111 samples: biplot of Principal Components 3 and 4 (29% of explained variance).
× = Liguria; • = Campania

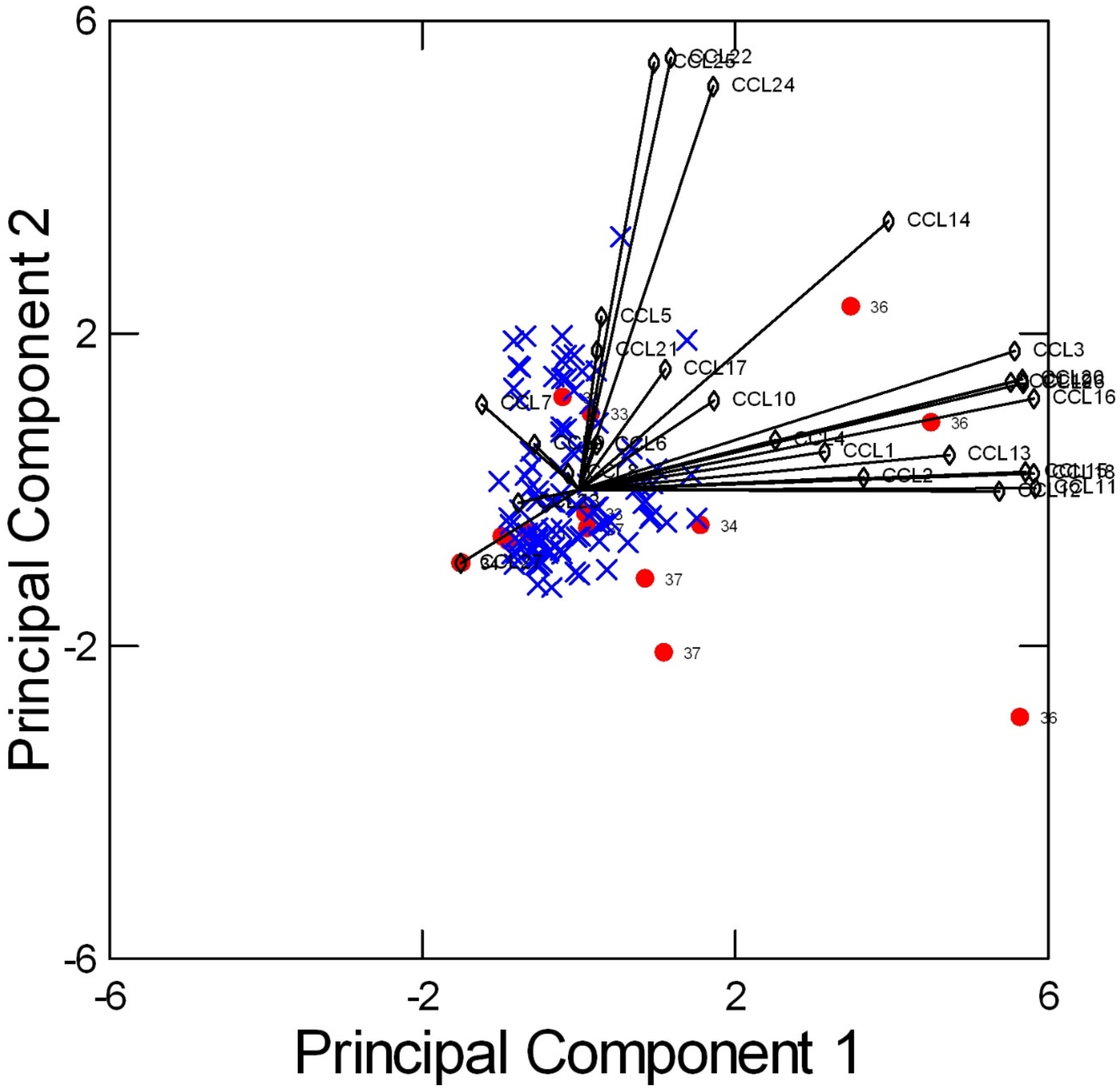

Figure S13. Results of PCA of Chenomx 500 MHz custom library metabolites (CCL) measured in 111 samples: biplot of Principal Components 1 and 2 (51% of explained variance).
× = Liguria; • = Campania

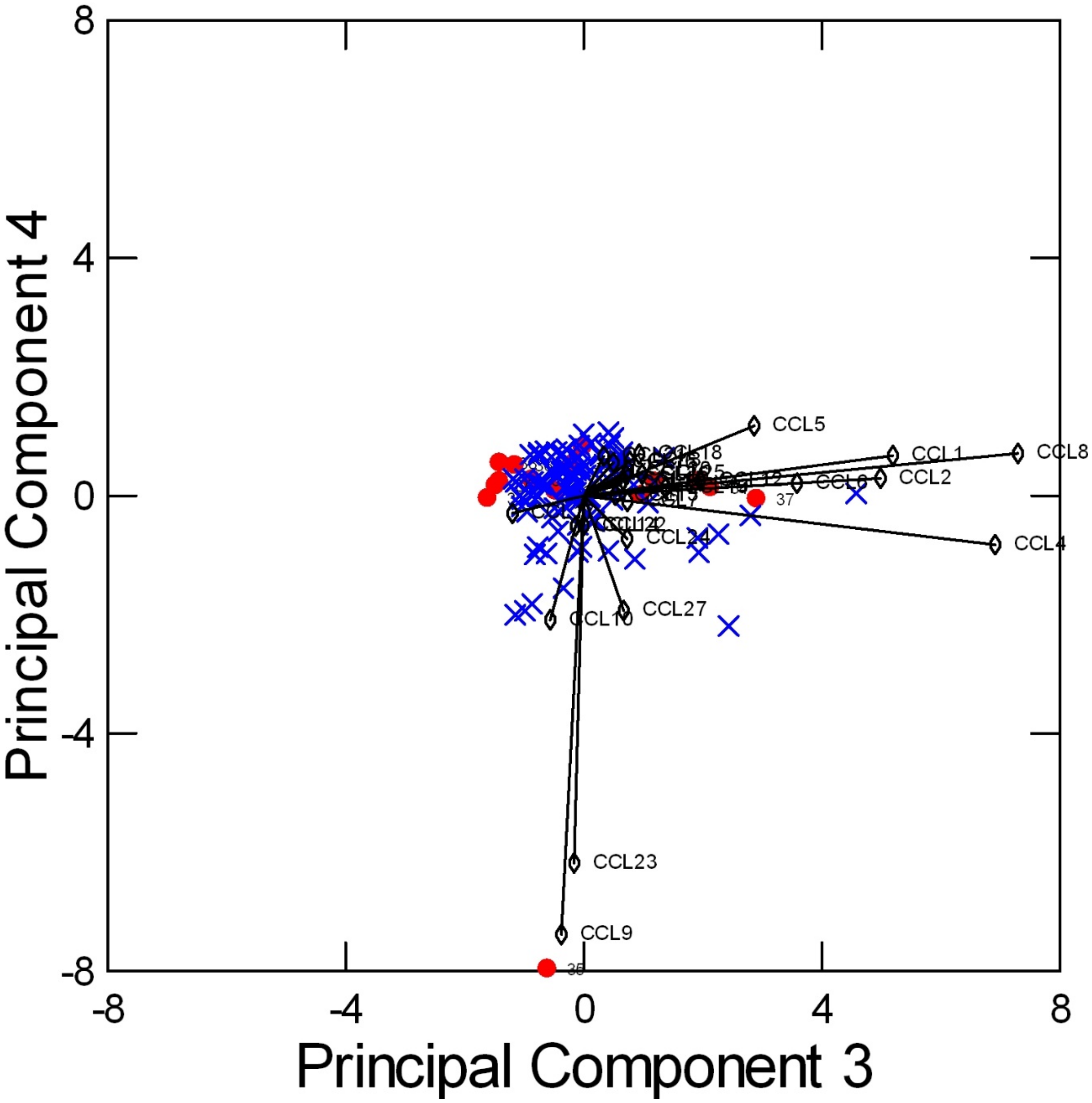

Figure S14. Results of PCA of Chenomx 500 MHz custom library (CCL) metabolites measured in 111 samples: biplot of Principal Components 3 and 4 (17% of explained variance).
× = Liguria; • = Campania

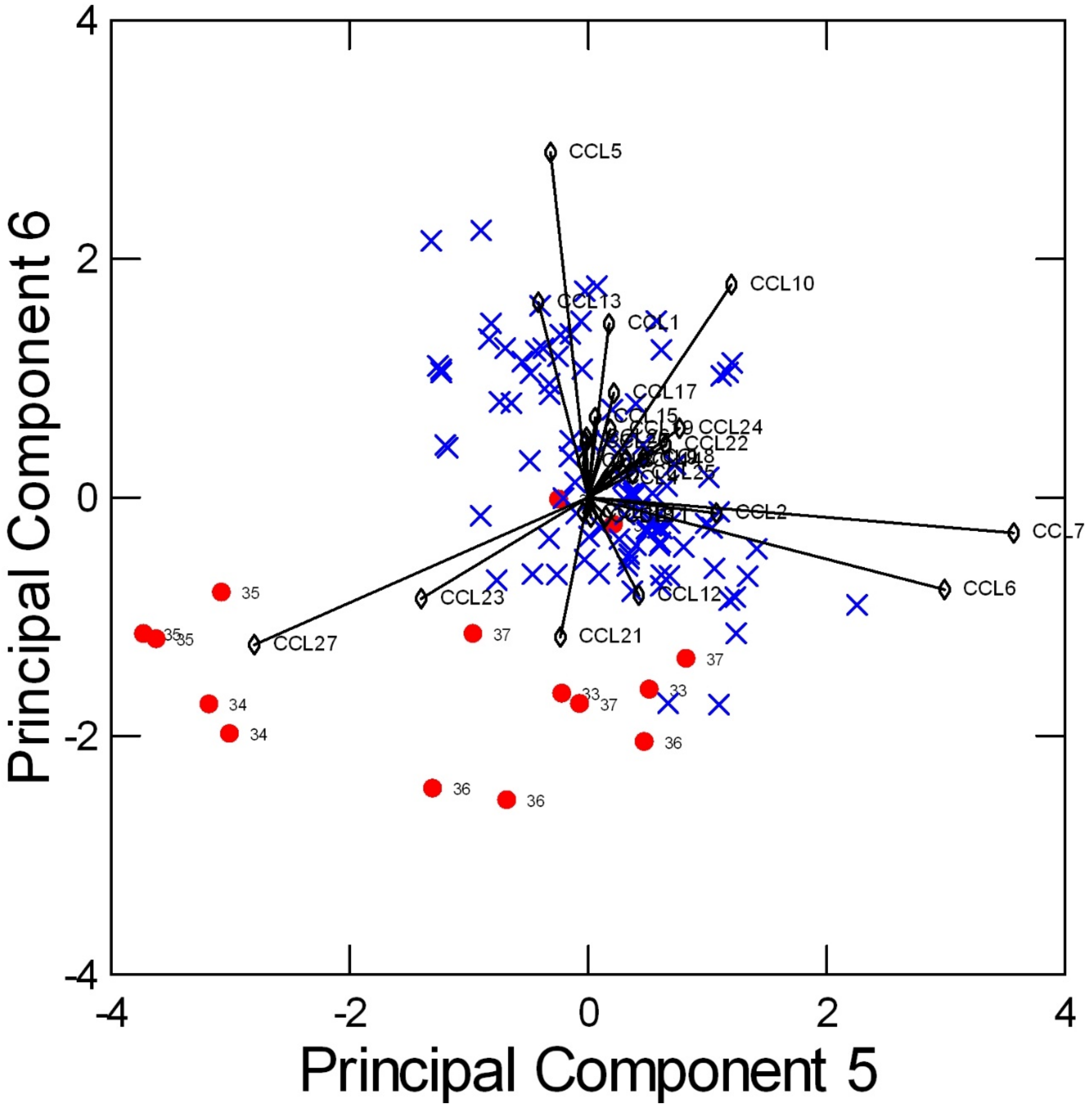

Figure S15. Results of PCA of Chenomx 500 MHz custom library metabolites (CCL) measured in 111 samples: biplot of Principal Components 5 and 6 (14% of explained variance).
× = Liguria; • = Campania

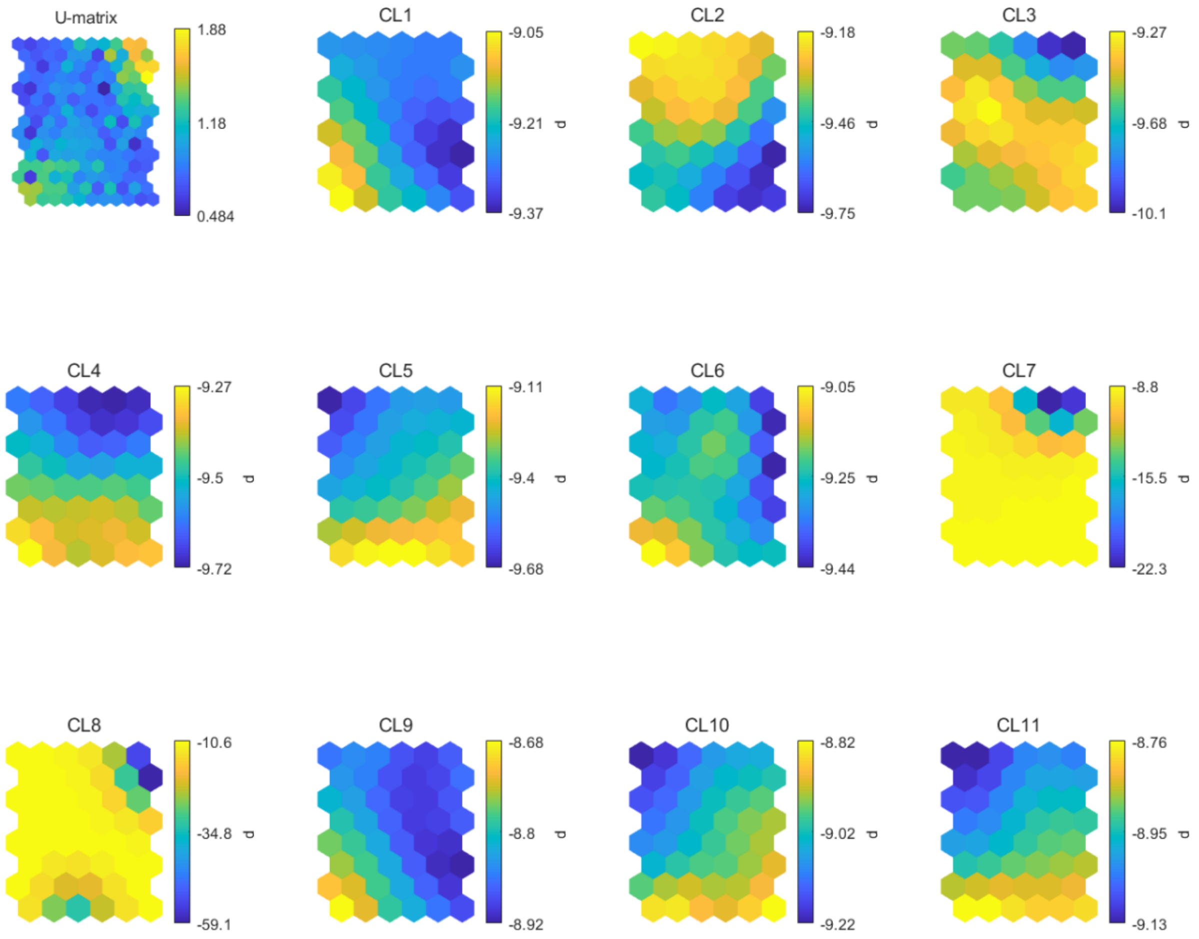

Figure S16. SOM, U-matrix and maps for each Chenomx 500 MHz version 11 library metabolite (CL). Similar color gradations indicate highly correlated variables.
CL1: alanine; CL2: acetate; CL3: malate; CL4: malonate; CL5: choline; CL6: fructose; CL7: sucrose; CL8: fumarate; CL9: valine; CL10: proline; CL11: asparagine.

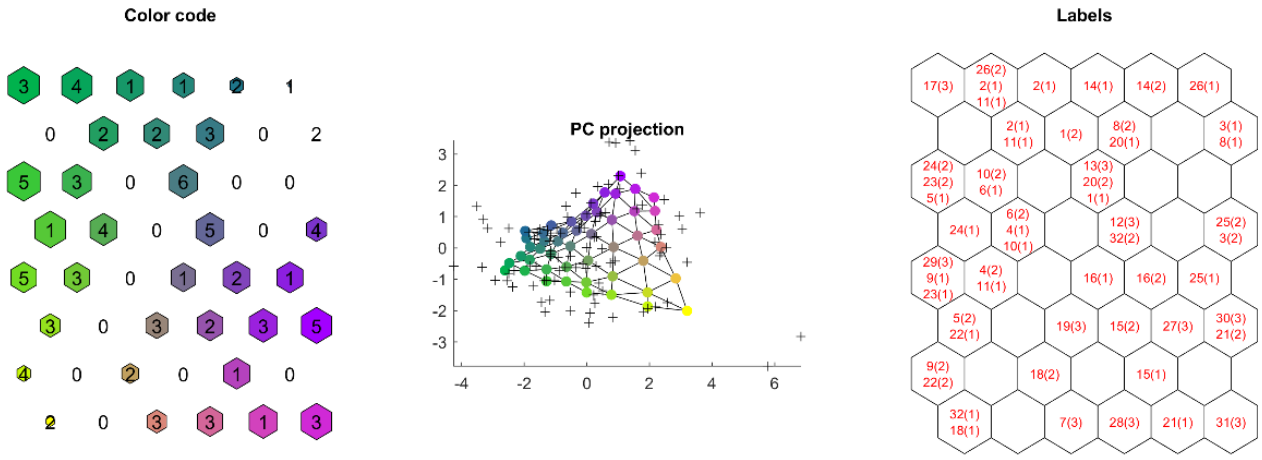

A B C

Figure S17. SOM: Graphical representation of map for Chenomx 500 MHz version 11 library metabolites (CL).
A. SOM output map with color code association. Similar colors have similar characteristics, numbers correspond to hit numbers. Dimensions of hexagons are related to the distance between neurons (biggest indicates greater distance); B. principal Component projection of the map; C. labelled SOM output map, for each neuron the corresponding accession number and number of replicates (in parentheses) are shown.

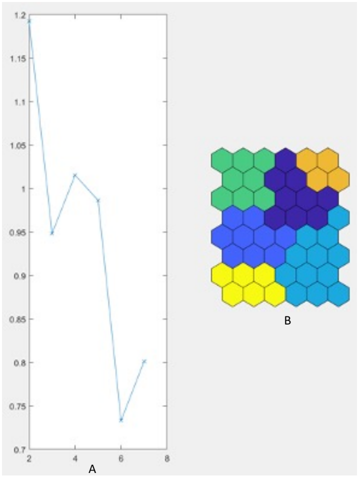

Figure S18. SOM: Map clusterization for Chenomx 500 MHz version 11 library metabolites (CL).
A. Davies-Bouldine index progression: minimum value fits the best number of clusters. B. 6 clusters.

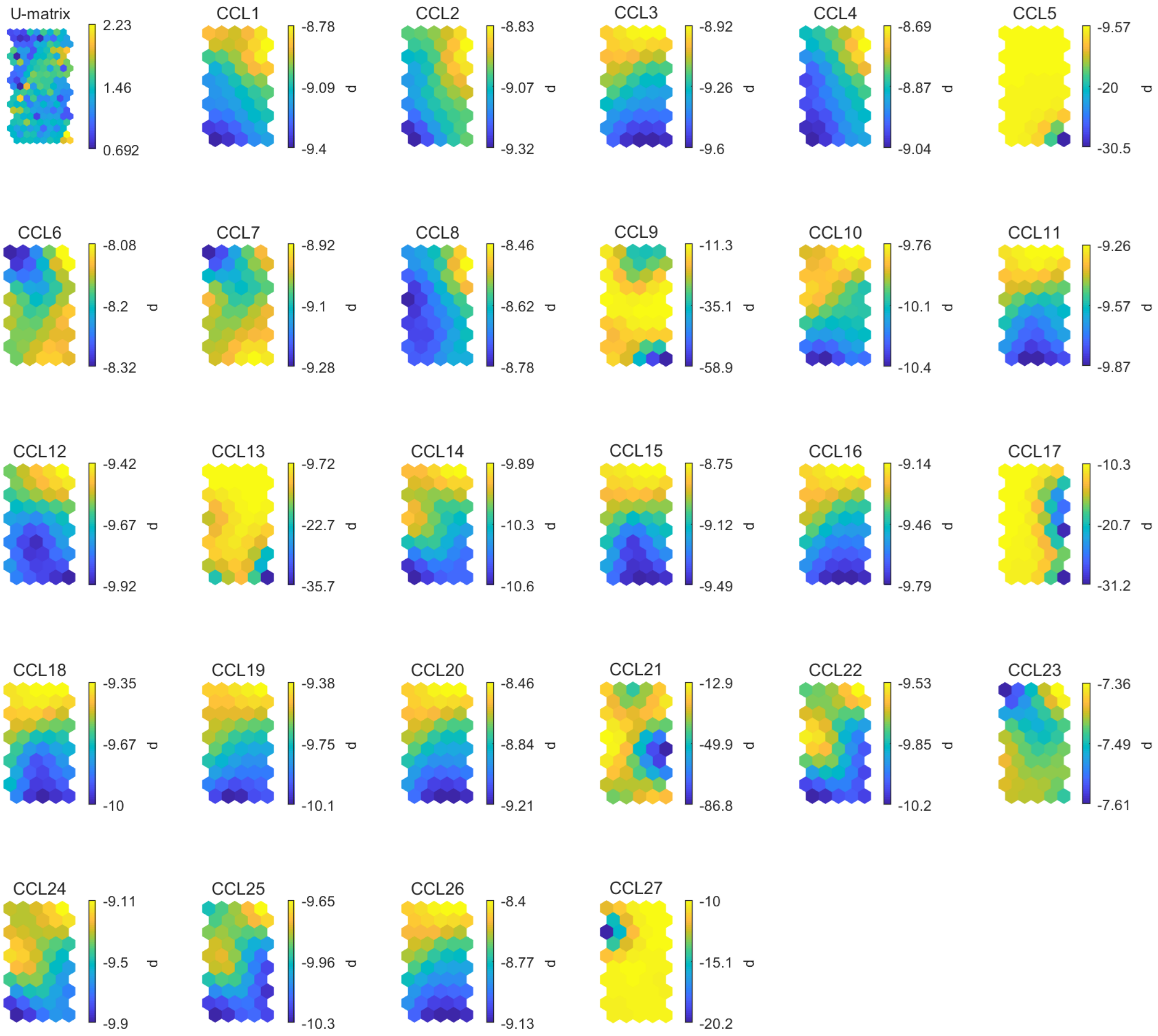

Figure S19. U-matrix and maps for each Chenomx 500 MHz custom library (CCL) metabolite. Similar color gradations indicate highly correlated variables. CCL1: 12-*O*-methylcarnosic acid; CCL2: 7-*O*-methylrosmanol; CCL3: methylrosmarinate; CCL4: genkwanin; CCL5: epicatechin; CCL6: isorhamnetin-3-*O*-β-D-rutinoside; CCL7: carnosic acid; CCL8: diosmetin; CCL9: luteolin; CCL10: scutellarein; CCL11: acacetin; CCL12: carnosol; CCL13: catechin hydrate; CCL14: gallic acid; CCL15: rosmarinic acid; CCL16: ferulic acid; CCL17: caffeic acid; CCL18: coumaric acid; CCL19: chlorogenic acid; CCL20: rutin; CCL21: quercetin; CCL22: apigenin; CCL23: isorosmanol; CCL24: apigenin-7-*O*-β-D-glycoside; CCL25: kaempferol; CCL26: isorhamnetin-3-*O*-rutinoside (narcissin); CCL27: epiisorosmanol.

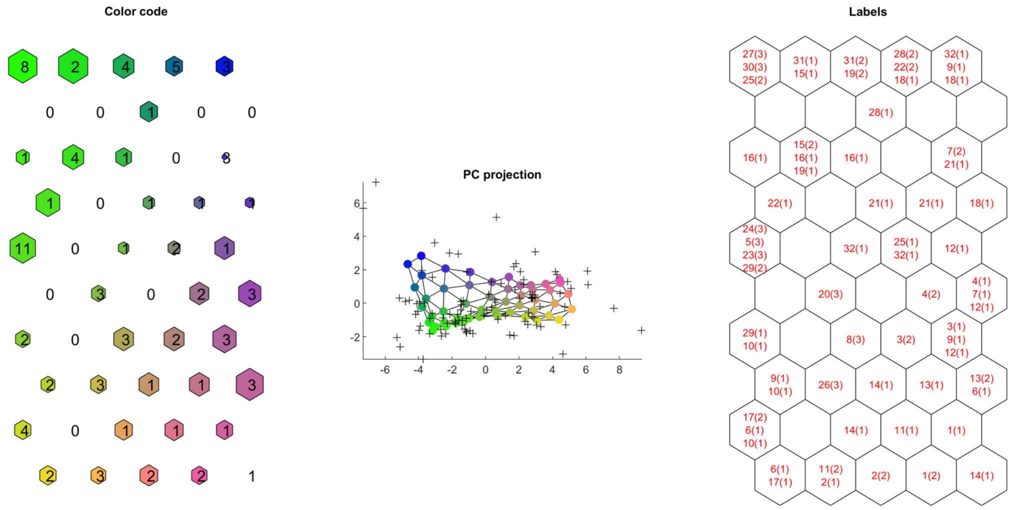

A B C

Figure S20. Graphical representation of Chenomx 500 MHz custom library (CCL) metabolites on map. A. SOM output map with colour code association. Similar colours have similar characteristics, numbers correspond to hit numbers. Dimensions of hexagons are related to the distance between neurons (biggest indicates greater distance); B. Principal component projection of the map; C. Labelled SOM output map, for each neuron the corresponding accession number (listed in Table S1, Supplementary Material) and number of replicates (in parentheses) are shown.

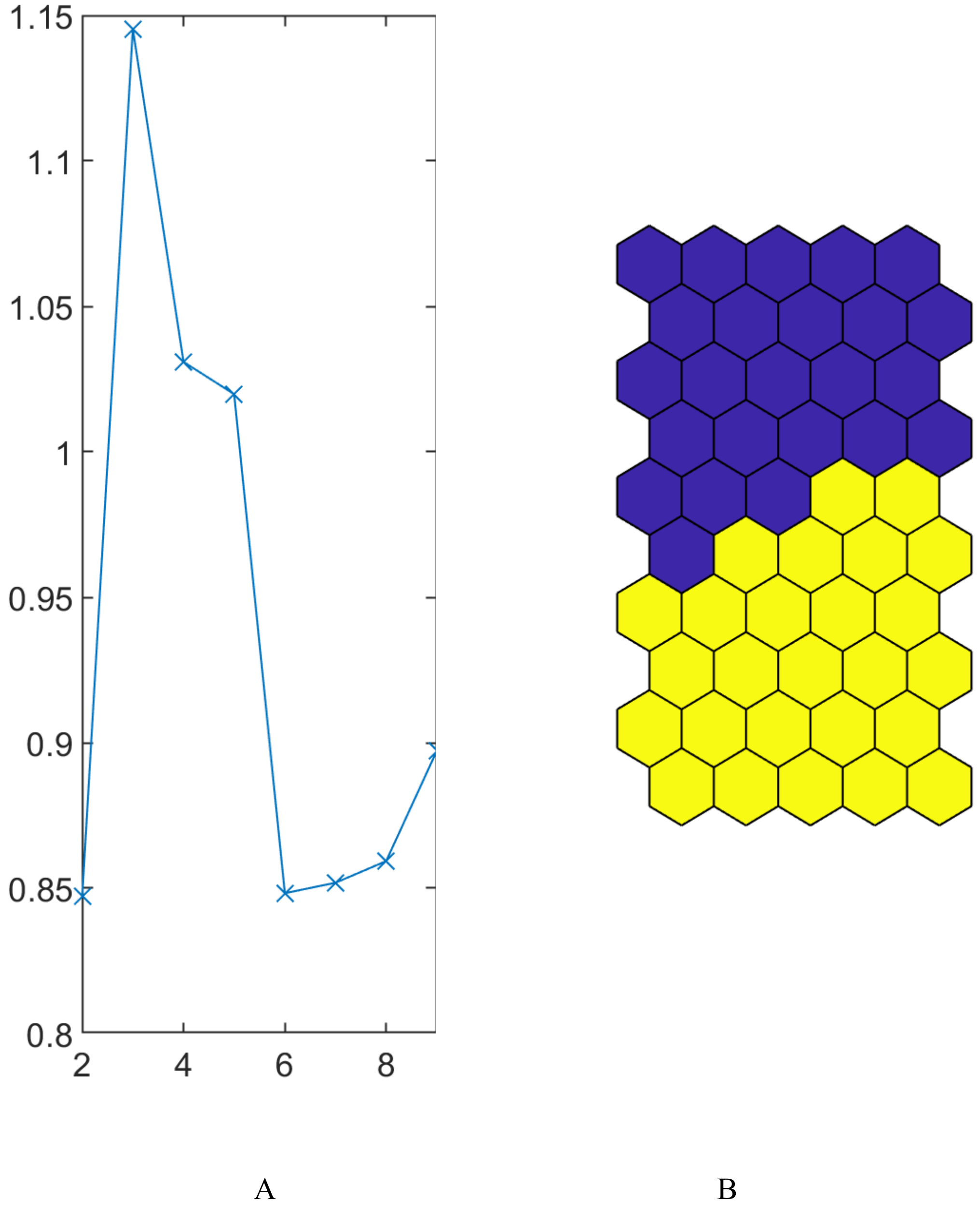

Figure S21. Map clusterization.
A. Davies-Bouldine index progression: minimum value fits the best number of clusters; B. 2 clusters.

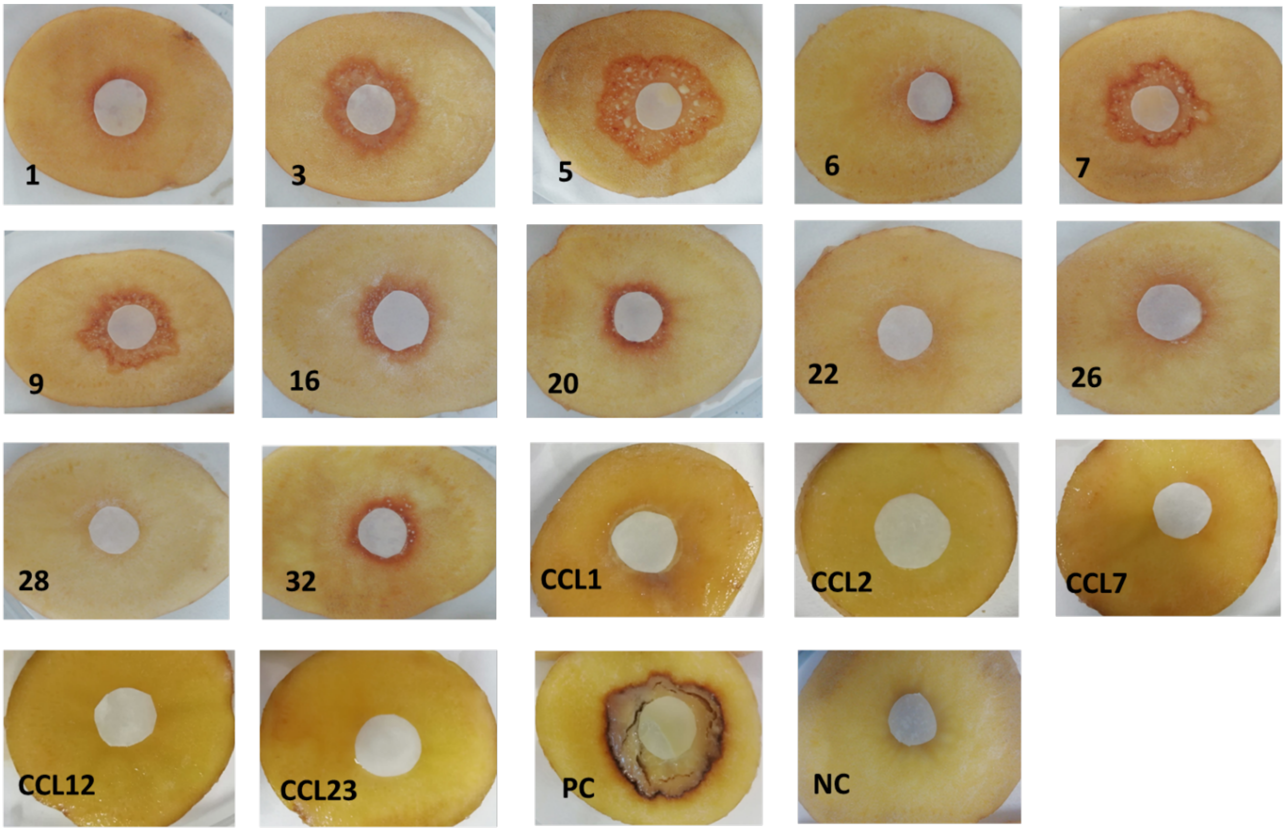
1
3
5
6
7
9
16
20
22
26
28
32
CCL1
CCL2
CCL7
CCL12
CCL23
PC
NC

a

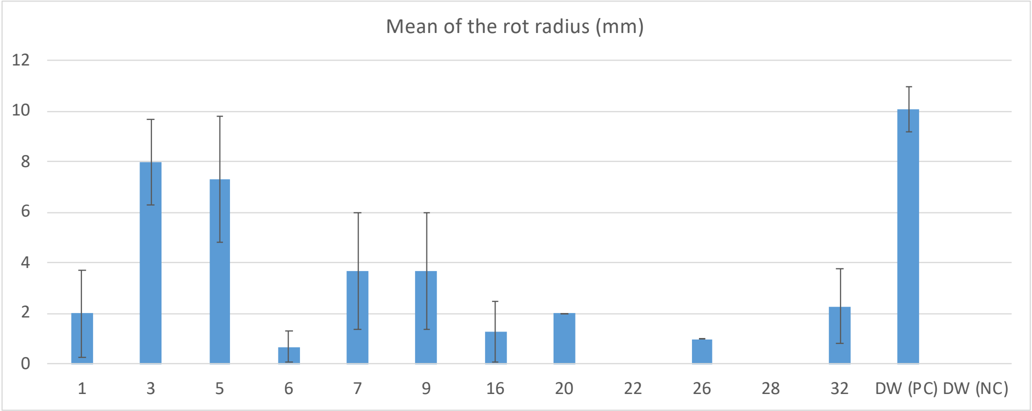
Mean of the rot radius (mm)
12
10
8
6
4
2
0
1
3
5
6
7
9
16
20
22
26
28
32
DW (PC)
DW (NC)

b

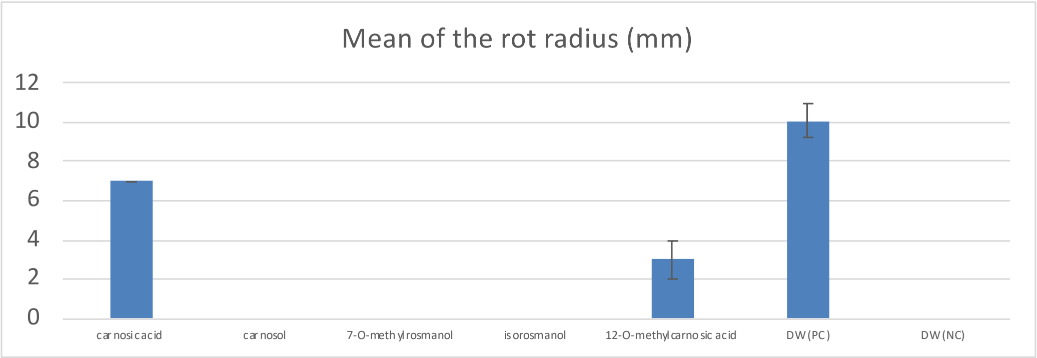
Mean of the rot radius (mm)
12
10
8
6
4
2
0
carnosic acid
carnosol
7-O-methylrosmanol
isorosmanol
12-O-methylcarnosic acid
DW (PC)
DW (NC)

c

Figure S22. Bacterial soft rot assay (a) and graphs of the results (b, c): potato tuber slices inoculated with the bacterial suspension of *P. carotovorum* subsp. *carotovorum* after 5 days of incubation at 25 °C.

The filter paper discs 10 mm in diameter were soaked with a bacterial suspension previously exposed for 5 minutes to the solutions of rosemary extracts (Table S1: 1, 3, 5, 6, 7, 9, 20, 22, 26, 28, 32) and pure compounds (Figure 6: CCL1: 12-*O*-methylcarnosic acid; CCL2: 7-*O*-methylrosmanol; CCL7: carnosic acid; CCL12: carnosol; CCL23: isorosmanol) in sterile distilled water and DMSO working solution (1:1 v/v); PC: positive control (paper disc was soaked with a solution of inoculated sterile distilled water and DMSO working solution 1:1 v/v PC); NC: negative control (paper disc soaked with a solution of not inoculated distilled water/DMSO working solution 1:1 v/v). The presence of tissue rotting, and the development of rot radius is directly correlated with the bacterial pectolytic activity. The exposure for 5 minutes to a solution of isorosmanol, carnosol and 7-*O*-methylrosmanol completely inhibited the bacterial pectolytic activity. At the same test conditions, 12-*O*-methylcarnosic acid inhibited the pectolytic activity by 70.3%, and carnosic acid by 30%, compared to the positive control (PC) (Table 2).

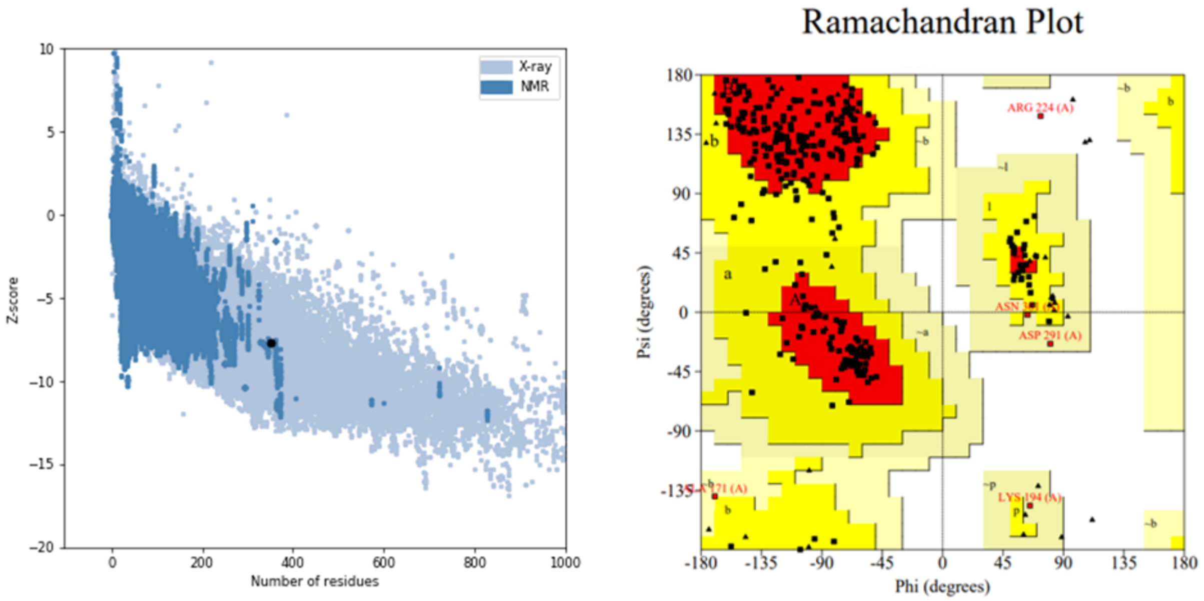

Figure S23. ProSA z-score (left) and Ramachandran plot (right) of the *P. carotovorum* pectate lyase 1 (PelA) homology model.

The z-score of -7.66 suggests that the homology model is properly folded and 99.6% of the protein residues being under the favoured region shows that almost all the protein residues are properly sterically placed in 3D space.

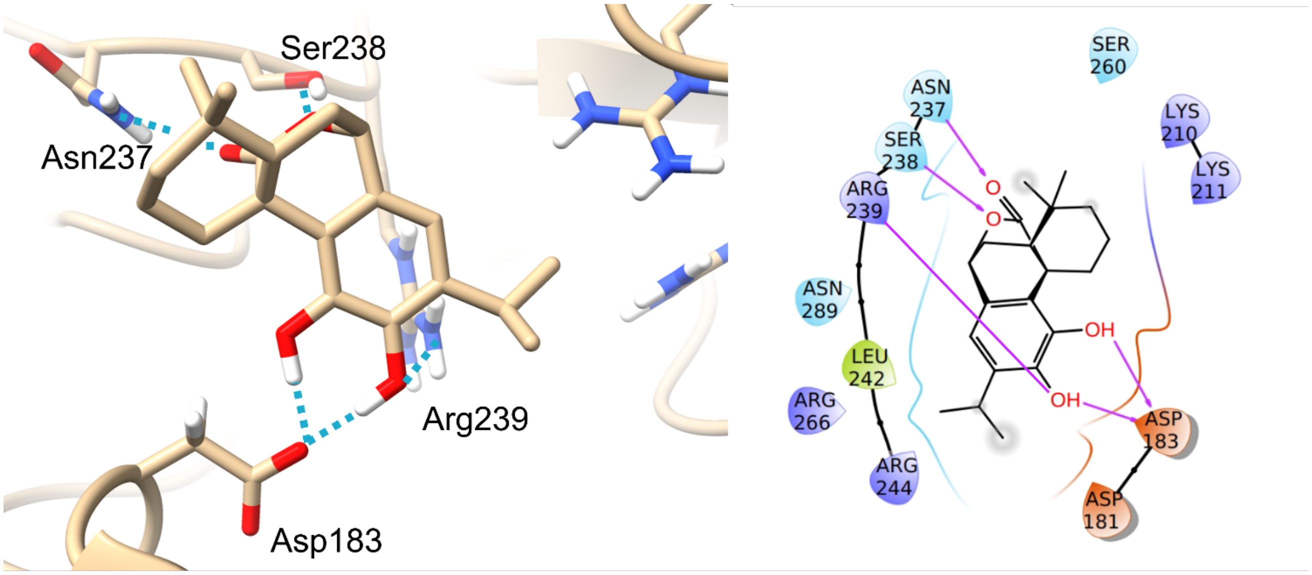

carnosol

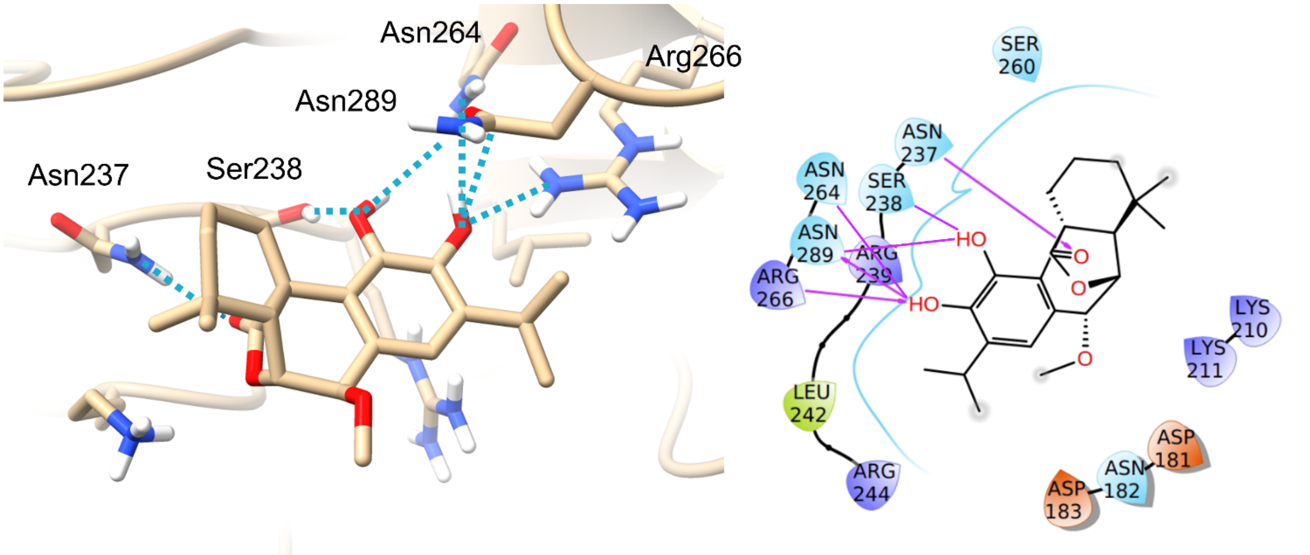

7-*O*-methyl-rosmanol

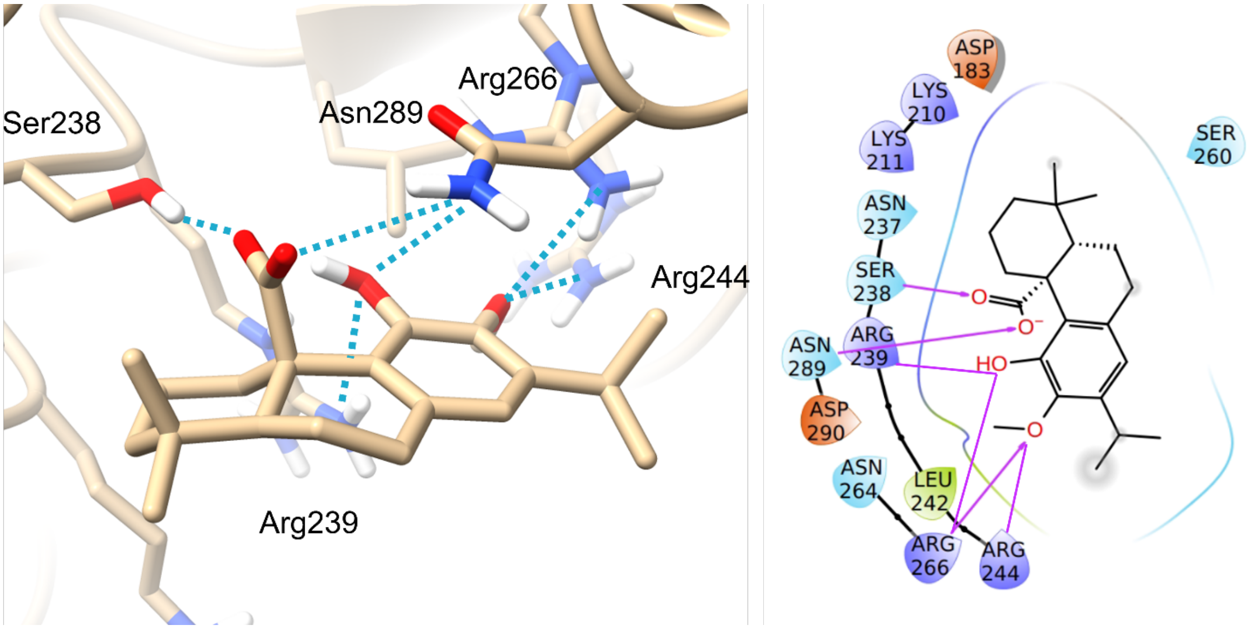

12-*O*-methyl-carnosic acid

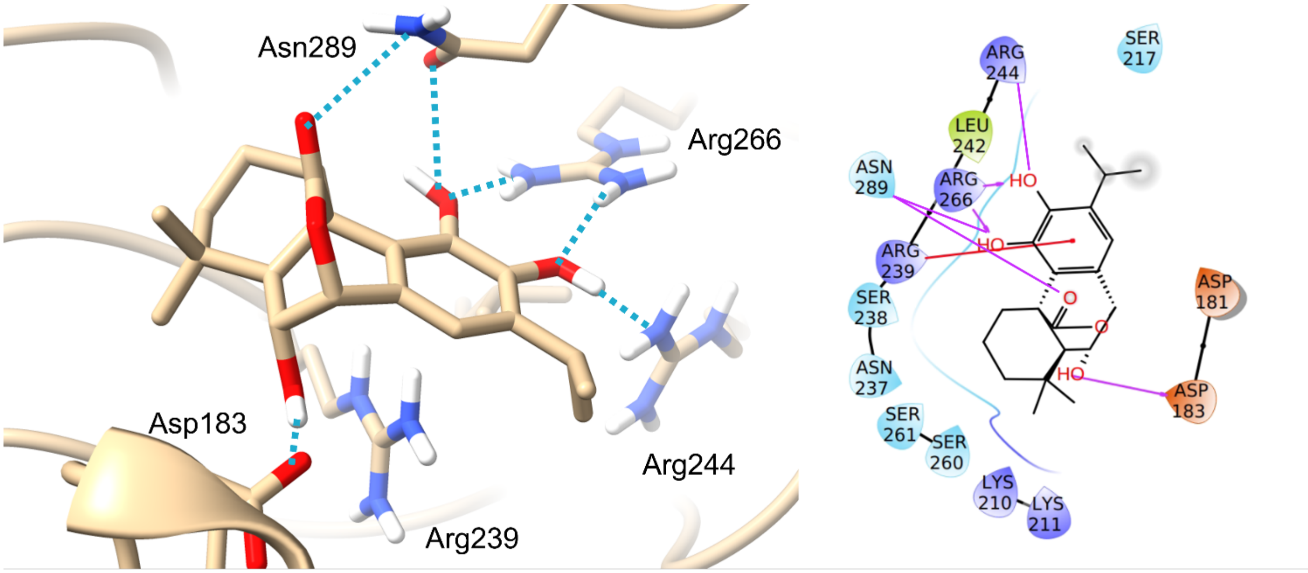

isorosmanol

Figure S24. Binding pose (left) and interaction (right) of the selected compounds docked to ligand binding site of *P. carotovorum* pectate lyase 1 (PelA).
Left) The protein is reported as light-brown ribbons; the ligand is reported as capped sticks; H-bonds are presented as cyan dotted lines. Right) The ligand is surrounded by the protein residues represented as follows: the negatively charged residues are indicated in red, polar residues are in cyan, hydrophobic residues are shown in green; H-bonds are depicted as purple arrows.

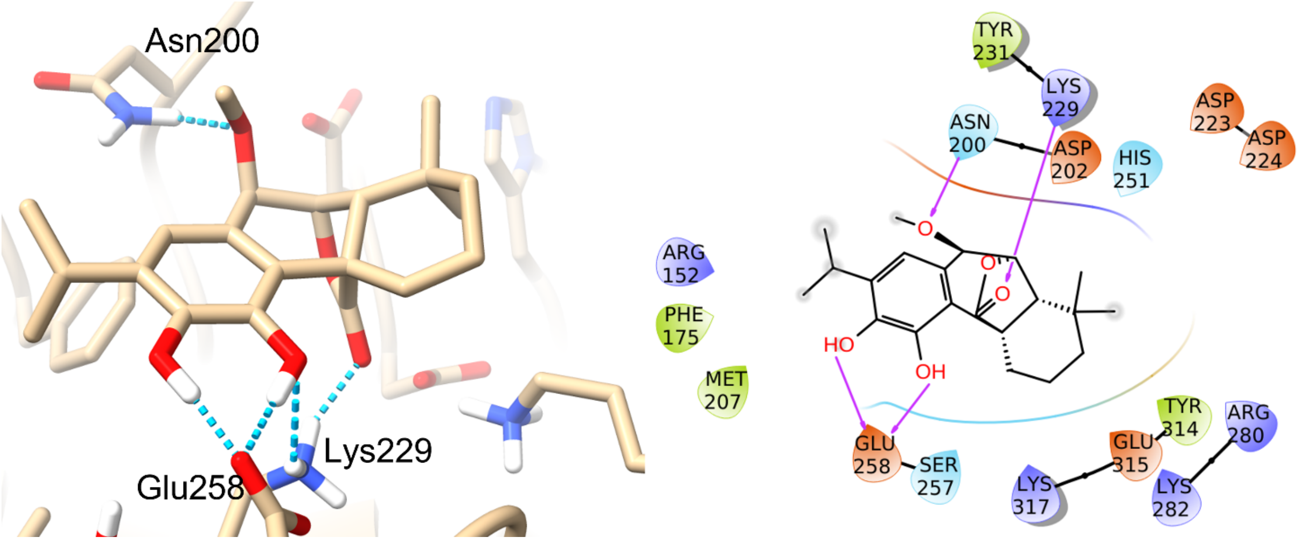

7-*O*-methyl-rosmanol

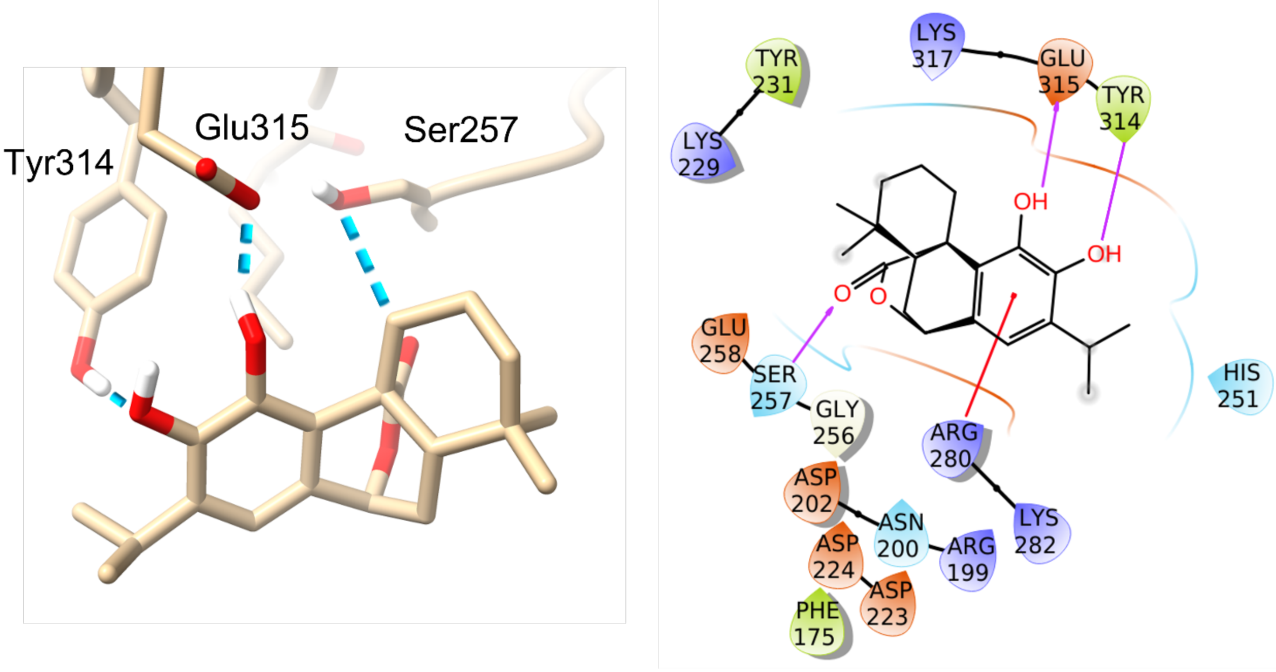

carnosol

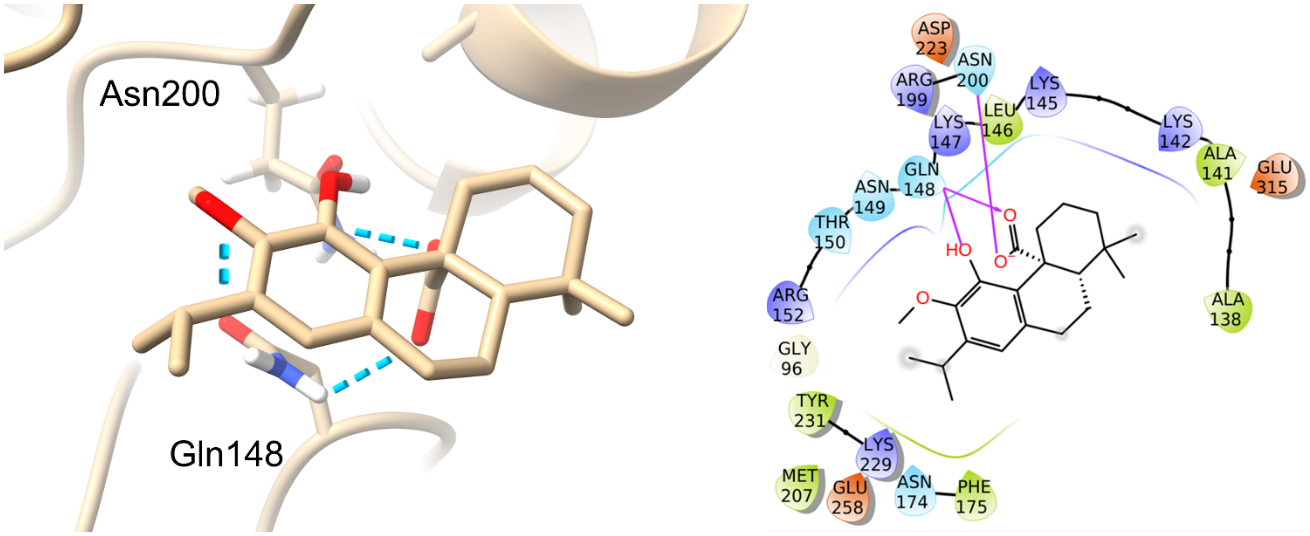

12-*O*-methyl-carnosic acid

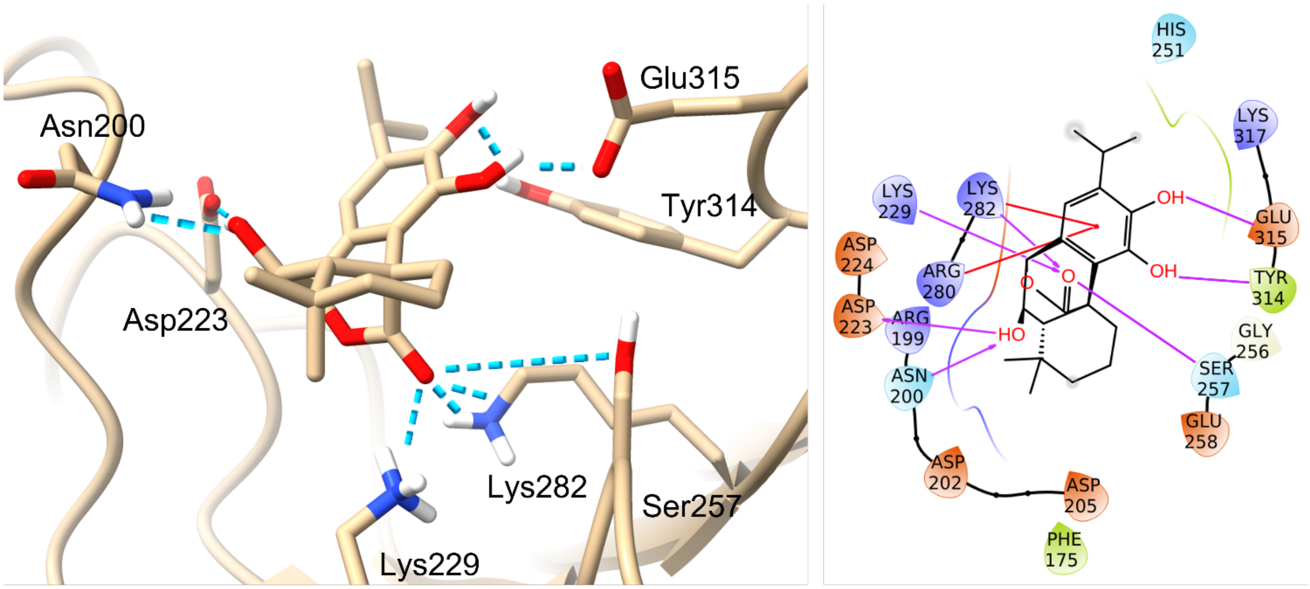

isorosmanol

Figure S25. Binding pose (left) and interaction (right) of the selected compounds docked to ligand binding site of *P. carotovorum* endo-polygalacturonase (PehA).
Left) The protein is reported as light-brown ribbons; the ligand is reported as capped sticks; H-bonds are presented as cyan dotted lines. Right) The ligand is surrounded by the protein residues represented as follows: the negatively charged residues are indicated in red, polar residues are in cyan, hydrophobic residues are shown in green; H-bonds are depicted as purple arrows.

1.2 Supplementary Tables

Table S1. List of rosemary accessions used in the study.

Accession number	Ecotype or Cultivar name	Grower[a]	Location[b]	Soil texture type[c]
1	"Eretto Liguria"	CeRSAA	Albenga	clay
2	"Eretto Liguria"	CeRSAA	Albenga	clay
3	"Eretto Liguria"	local farmer	Savona	clay loam
4	"Eretto Liguria"	local farmer	Vessalico	clay
5	"Eretto Liguria"	local farmer	Vessalico	clay
6	'Prostratus' (Prostrata Group)	local farmer	Ruta di Camogli	clay
7	"Eretto" (local ecotype)	local farmer	Ruta di Camogli	clay
8	"Eretto" (local ecotype)	local farmer	Ruta di Camogli	clay
9	'Prostratus' (Prostrata Group)	local farmer	Ruta di Camogli	clay
10	'Prostratus' (Prostrata Group)	local farmer	Ruta di Camogli	clay
11	'Prostratus' (Prostrata Group)	local farmer	Ruta di Camogli	clay
12	"Eretto Liguria"	CREA	Sanremo	organic[d]
13	"Eretto Liguria"	CREA	Sanremo	organic[d]
14	"Eretto Liguria"	CREA	Sanremo	organic[d]
15	'Boule' (Prostrata Group, 'Rampant Boule')	CREA	Sanremo	organic[d]
16	'Boule' (Prostrata Group, 'Rampant Boule')	CREA	Sanremo	organic[d]
17	'Boule' (Prostrata Group, 'Rampant Boule')	CREA	Sanremo	organic[d]
18	'Joyce DeBaggio'	CREA	Sanremo	organic[d]
19	'Joyce DeBaggio'	CREA	Sanremo	organic[d]
20	'Joyce DeBaggio'	CREA	Sanremo	organic[d]
21	'Benenden Blue'	CREA	Sanremo	organic[d]
22	'Benenden Blue'	CREA	Sanremo	organic[d]
23	'Benenden Blue'	CREA	Sanremo	organic[d]
24	'Majorca Pink'	CREA	Sanremo	organic[d]
25	'Majorca Pink'	CREA	Sanremo	organic[d]
26	'Majorca Pink'	CREA	Sanremo	organic[d]
27	"Porto Alabe"	CREA	Sanremo	organic[d]
28	"Porto Alabe"	CREA	Sanremo	organic[d]
29	"Porto Alabe"	CREA	Sanremo	organic[d]
30	'Santa Barbara Blue'	CREA	Sanremo	organic[d]
31	'Santa Barbara Blue'	CREA	Sanremo	organic[d]
32	'Santa Barbara Blue'	CREA	Sanremo	organic[d]
33	"Porto Alabe"	local farmer	Pesco Sannita	clay loam
34	'Gorizia'	local farmer	Benevento	clay
35	'Boule' (Prostrata Group, 'Rampant Boule')	local farmer	Benevento	clay
36	'Farinole'	local farmer	San Marco di Castellabate	sand
37	'Capri' (Prostrata Group, 'Capri')	local farmer	Seiano di Vico Equense	sand

[a] CeRSAA: Centro di Sperimentazione e Assistenza Agricola, Regione Rollo 98, 17031 Albenga, Italy; CREA - Research Centre for Vegetable and Ornamental Crops, Corso Inglesi 508, 18038 Sanremo, Italy.
[b] Albenga (SV): latitude 44°02′56.81″N, longitude 8°12′46.86″E, elevation 5 m.a.s.l; Savona (SV): latitude 44°18′28.71″N, longitude 8°28′51.66″E, elevation 4 m.a.s.l; Vessalico (IM): latitude 44°02′46.74″N, longitude 7°57′43.43″E, elevation 197 m.a.s.l; Ruta di Camogli (GE): latitude 44°20′36.68″N, longitude 9°10′10.29″E, elevation 269 m.a.s.l; Sanremo (IM): latitude 43°49′05.23″N, longitude 7°′26.12″E, elevation 80 m.a.s.l; Pesco Sannita (BN): latitude 41°14′N, longitude 14°49′E, elevation 8393 m.a.s.l; Benevento (BN): latitude 41°08′N, longitude 14°47′E, elevation 135 m.a.s.l; San Marco di Castellabate (SA): latitude 40°16′02.64″N, longitude 14°56′20.33″E, elevation 15 m.a.s.l; Seiano di Vico Equense (NA): latitude 40°40′N, longitude 14 14°26′E, elevation 85 m.a.s.l.
[c] (Yolcubal et al., 2004).
[d] grown in the experimental fields of CREA, in the open air. The cultivars were propagated via stem cuttings and were grown in 20 L black plastic pots with a commercial substrate (Terfor Hochmoor® Vulcan Invernale pumex) suitable for nursery crops. A controlled release fertilizer (Geogreen Horti-Cote®Plus, 6 months release period, N–P–K–Mg 16–6–11–2 plus microelements) was added in the amount of 5 g/L of substrate. Fertigation was performed monthly with Ferty 1 (Planta Düngemittel GmbH, N–P–K–Mg 20–7–10–2, 1.5 g/L).

Table S2. Rosemary accessions data.

Ecotype or Cultivar name	Plant habitus	Status	Register type[a] / references	Synonyms[b]	Voucher[c]
"Eretto Liguria"	erect	Liguria (Italy) local ecotype	-	-	HMGBH. e/7219.20 23.006
'Prostratus' (Prostrata Group)	prostrate (matforming)	accepted, registered	COM (CPVO, 2022; RHS, 2022)	*R. officinalis* Prostratus Group; *R. officinalis* 'Corsica Prostratus'; *R. officinalis* 'Venzano Prostrate'; *R. corsicus* 'Prostratus'; *S. × lavandulacea* misapplied; *R. officinalis* lavandulaceus; *R. officinalis* repens; *R. officinalis* creeping; *R. × lavandulaceus* misapplied; *R. repens; R. officinalis* var. *prostratus*	HMGBH. e/7219.20 23.007
'Boule' (Prostrata Group, 'Rampant Boule')	prostrate (trailing)	accepted, registered	COM (CPVO, 2022; RHS, 2022)	*Rosmarinus* 'Boule'; *R. officinalis* (Prostratus Group) 'Rampant Boule'; *R. officinalis* (Prostratus Group) 'Boule'	HMGBH. e/7219.20 21.003
'Joyce DeBaggio'	erect (upright)	registered	COM (Begum et al., 2013; CPVO, 2022)	-	HMGBH. e/7219.20 21.002
'Benenden Blue' (Angustifolia Group, 'Benenden Blue')	erect (upright, bushy)	accepted, registered	COM (Begum et al., 2013; CPVO, 2022; RHS, 2022)	*R. officinalis* 'Benenden Blue'; *R. officinalis* 'Collingwood Ingram'; *R. officinalis* var. *angustissimus* 'Benenden Blue'; *S. rosmarinus* 'Collingwood Ingram'	HMGBH. e/7219.20 23.001)
'Majorca Pink'	semi-erect (upright, bushy)	accepted, registered	COM (Begum et al., 2013; CPVO, 2022; RHS, 2022)	*R. officinalis* 'Majorca Pink'	HMGBH. e/7219.20 17.003
"Porto Alabe"	semi-erect	Sardinia (Italy) local ecotype	-	-	HMGBH. e/7219.20 23.002
'Santa Barbara Blue'	erect	unresolved, registered	COM (Begum et al., 2013; CPVO, 2022; RHS, 2022)	*R. officinalis* 'Santa Barbara Blue'	HMGBH. e/7219.20 23.003
'Gorizia'	erect (upright, bushy)	accepted, registered	COM (CPVO, 2022; RHS, 2022)	*R. officinalis* 'Gorizia'	HMGBH. e/7219.20 21. 001
'Farinole'	semi-erect	accepted, registered	COM (CPVO, 2022; RHS, 2022)	*R. officinalis* 'Farinole'	HMGBH. e/7219.20 23.005
'Capri' (Prostrata Group, 'Capri')	trailing	accepted, registered	COM (CPVO, 2022)	*R. officinalis* (Prostratus Group) 'Capri'	HMGBH. e/7219.20 23.004

[a] COM = Commercial register
[b] (RHS, 2022)
[c] The vouchers of all the accessions were deposited at the Herbarium of Giardini Botanici Hanbury (La Mortola, Ventimiglia, Italy)

Table S3. Brief description of the commercial cultivars used in the study.

Cultivar	Common name	Origin	Description
'Prostratus' (Prostrata Group).	Rosemary Prostrata Group (RHS, 2022)	Mediterranean area	Compact evergreen shrubs of prostrate mat-forming habit, with narrowly linear, aromatic thick, needle-like leaves, spirally arranged around thin woody stems. Pale blue flowers in small axillary clusters in spring and summer, occasionally in autumn. (RHS, 2022)
'Boule'	Rosemary 'Rampant Boule' (RHS, 2022)	France	Prostrate (trailing) habitus, 60 cm high at maturity. Highly pubescent internode, 8.8-10.6 mm long and 2.6-3.4 mm wide. Elliptical dark green leaves 19.6-24.0 mm long and 2.4-2.8 mm wide; acute apex. Inflorescence 14.8-17.8 mm long. Calix 5.0-6.1 mm long, and highly pubescent. Pale purplish-blue corolla (UPOV 50)[a] 10.5-11.2 long; 5.8-6.5 mm wide lower lip with scarce concavity, white central stripe, and dark purplish blue discontinuous lateral stripes (UPOV 52)[a]. (Cervelli and Masselli, 2013)
'Joyce DeBaggio'	Rosemary, Golden Rain (Begum et al., 2013)	Virginia (USA) – cultivar selected by Thomas De Baggio	Semi - upright habitus, 90 cm high at maturity. Highly pubescent internode 10.8-13.2 mm long and 3.3-4.5 mm wide. Variegated leaves (light green in central zone and golden-yellow at leaf margins) 28.2-33.4 mm long and 1.8-2.4 mm wide; acute apex. Inflorescence 12.3-14.6 mm long. Calix 4.0-4.6 mm long, and highly pubescent. Pale purplish-blue corolla (UPOV 50)[a] 7.3-9.1 long; 5.4-6.6 mm wide, lower lip with moderate concavity, white central stripe, and dark purplish blue lateral stripes (UPOV 52)[a] (Begum et al., 2013; Cervelli and Masselli, 2013).
'Benenden Blue'	Rosemary, Benenden Blue (Begum et al., 2013; RHS, 2022)	Corsica. Cultivar deriving from a chemotype selected by Collingwood Ingram (UK) in 1930	Upright or semi - upright habitus, 90 cm high at maturity. Poorly pubescent internode 10.5-13.1 mm long and 2.3-2.5 mm wide. Light green leaves 17.8-23.0 mm long and 1.8-2.2 mm wide; acute apex. Inflorescence 18.0-20.0 mm long. Calix 4.2-5.2 mm long, and poorly pubescent. Pale purplish-blue corolla (UPOV 50)[a] 9.2-10.1 long; 4.2-5.2 mm wide lower lip with high concavity, white central stripe, and discontinuous lateral stripes and medium purplish-blue dots (UPOV 51)[a] (Begum et al., 2013; Cervelli and Masselli, 2013).
'Majorca Pink'	Rosemary, Spanish (Begum et al., 2013), rosemary 'Majorca Pink' (RHS, 2022)	Spain	Urpright (bushy) or semi - upright habitus, 110 cm high at maturity. Highly pubescent internode 10.6-16.8 mm long and 2.1-3.9 mm wide. Elliptic light green leaves 10.3-14.7 mm long and 1.5-1.7 mm wide; acute apex. Inflorescence 16.6-18.2 mm long. Calix 4.1-4.9 mm long with highly pubescent. Pale violet corolla (UPOV 44)[a] 9.7-11.5 long; 5.9-7.1 mm wide lower lip with moderate concavity, white central stripe, and medium violet lateral stripes and dots (UPOV 45)[a] (Cervelli and Masselli, 2013).
"Porto Alabe"	-	Sardinia (Italy), accession collected and commercialized by an Italian farmer, "Il Bolfone", Cherasco (CN), and given to CREA collection	Semi - upright habitus, 70 cm high at maturity. Poorly pubescent internode 10.0-12.8 mm long and 2.0-2.6 mm wide. Light green leaves 20.2-25.6 mm long and 1.9-2.2 mm wide; acute apex. Inflorescence 14.4-17.6 mm long. Calix 4.4-5.0 mm long and highly pubescent. Pale purplish-blue corolla (UPOV 50)[a] 9.9-10.9 long; 3.4-4.3 mm wide lower lip with moderate concavity, white central stripe, and lateral stripes and medium purplish-blue dots (UPOV 51)[a] (Cervelli and Masselli, 2013).
'Santa Barbara Blue'	-	USA	Upright habitus, 110 cm high at maturity. Highly pubescent internode 9.3-11.5 mm long and 2.4-3.2 mm wide. Light green leaves 24.1-29.3 mm long and 2.1-2.7 mm wide; acute apex. Inflorescence 15.9-18.9 mm long. Calix 4.6-5.3 mm long and highly pubescent. Medium light blue corolla (UPOV 54)[a] 9.5-10.8 long; 5.2-6.5 mm wide lower lip with moderate concavity, white central stripe, and medium purplish blue lateral stripes (UPOV 51)[a] (Cervelli and Masselli, 2013).
'Gorizia'	Rosemary Gorizia (Begum et al., 2013; RHS, 2022)	-	'Gorizia' is a vigorous, aromatic, evergreen shrub with narrow, upright shoots to 1.5m. Internode 12.0-19.5 mm long and 4.5-6.5 mm wide. Fragrant dark green leaves twice the size of most cultivars: 22.8-33.8 mm long and 3.0-4.5 mm wide; acute apex. Inflorescence 15.3-22.7 mm long. Calix 6.2-7.6 mm long and highly pubescent. Relatively large, light violet-blue corolla (UPOV 50)[a] 15.3-22.7 long; 6.2-7.6 mm wide lower lip with moderate concavity, white central stripe, and medium purplish blue lateral stripes (UPOV 51)[a] appear in spring and often again in autumn (Begum et al., 2013; RHS, 2022).
'Farinole'	Rosemary 'Farinole'(RHS, 2022)	Corse (France)	Perennial evergreen shrub, with fine foliage, and very fragrant essential oil. The flower is blue. The plant reaches a maximum height of 30 - 35 cm (Malcuit and Deleuil, 1957; Paradis, 2004; Jeanmonod, 2015).
'Capri' (Prostrata Group, 'Capri')	Rosemary 'Capri' (RHS, 2022)	-	'Capri' is a highly aromatic, low, spreading, evergreen shrub to about 15cm high and a metre or more across, with bright green, needle-like foliage. Small, pale blue flowers are produced in spring and often again in autumn (RHS, 2022).

[a] UPOV color group (UPOV, 2018)

Table S4. Morphological traits of the ecotype "Eretto Liguria".

Common name	Origin	Description
"Eretto Liguria"	Unknown - mostly cultivated in Ligurian region	Ecotype with upright habitus, 120 cm high at maturity. Poorly pubescent internode 12.9-14.3 mm long and 2.3-3.2 mm wide. Elliptical light green leaves 23.5-29.1 mm long and 2.4-3.1 mm wide; acute apex. Inflorescence 15.6-23.2 mm long. Calix 5.1-5.8 mm long with anthocyanin colouration and highly pubescent. Pale purplish-blue corolla (UPOV 50)[a] 10.5-11.7 long; 5.1-6.0 mm wide lower lip with moderate concavity, white central stripe, and purplish blue discontinuous lateral stripes (UPOV 51)[a].

[a] UPOV color group (UPOV, 2018)

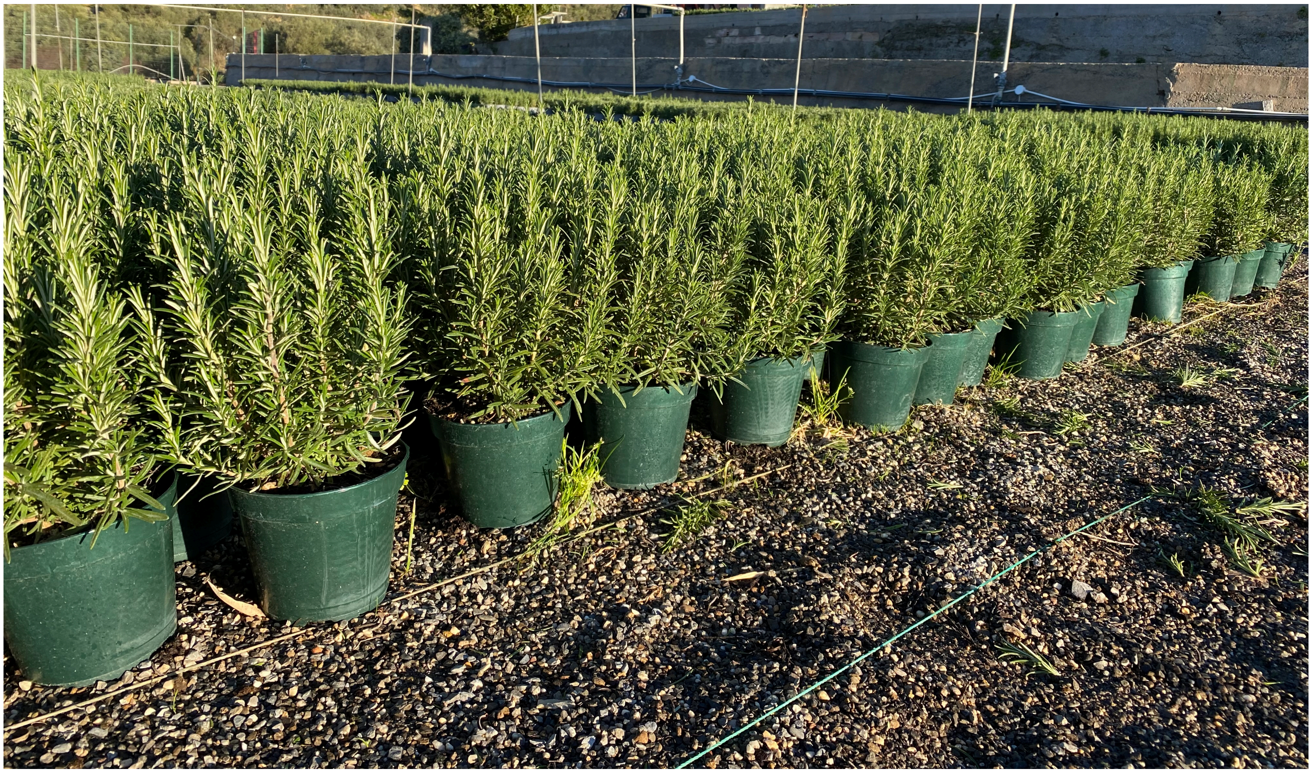

Salvia rosmarinus "Eretto Liguria"

Table S5. ^{1}H NMR chemical shifts (δ) and coupling constants (Hz) of Chenomx 500 MHz custom library metabolites (CCL) identified by using 1D spectra in CD_3OD-KH_2PO_4 in D_2O at pH 6.0, 500 MHz.

CCL number	metabolite	selected characteristic NMR chemical shifts in ppm (multiplicity, *J*)	other chemical shifts in ppm (multiplicity, *J*)	MSI Status [b]	references
**1**	12-*O*-methylcarnosic acid	3.69 (s)	6.48 (s), 3.43 (m), 3.16 (m), 2.77 [a], 2.75 [a], 2.55 (m), 1.91 (m), 1.60 (m), 1.52 [a], 1.47 [a], 1.32 [a], 1.16 (d, *J* = 6.5 Hz), 1.14 (d, *J* = 6.5 Hz), 1.10 [a]	1	(Richheimer et al., 1996; Pukalskas et al., 2005)
**2**	7-*O*-methylrosmanol	3.67 (s)	6.79 (s), 4.87 (d, *J* = 3.2 Hz), 4.31 (d, *J* = 3.2 Hz), 3.27 [a], 3.19 [a], 1.95 [a], 1.93 [a], 1.62 (m), 1.45 [a], 1.42 [a], 1.25 (m), 1.19 [a], 1.17 [a], 1.02 (s), 0.91 (s)	1	(Richheimer et al., 1996)
**3**	methylrosmarinate	7.01 (dd, *J* = 8.0, 1.8 Hz)	7.56 (d, *J* = 15.9 Hz), 7.10 (d, *J* = 1.8 Hz), 6.85 (dd, *J* = 8.0, 1.8 Hz), 6.77 [a], 6.76 [a], 6.61 (d, *J* = 8.0 Hz), 6.29 (d, *J* = 15.9 Hz), 5.21 (dd, *J* = 7.7, 5.0 Hz), 3.73 (s), 3.08 [a], 3.03 [a]	1	(Kuo et al., 2000; Correia et al., 2020)
**4**	genkwanin	3.92 (s)	7.92 (dd, *J* = 8.4, 2.0 Hz), 7.00 (dd, *J* = 8.4, 2.0 Hz), 6.72 (d, *J* = 2.2 Hz), 6.70 (s), 6.40 (d, *J* = 2.2 Hz),	1	(Gomes et al., 2011)
**5**	epicatechin	4.23 (s)	6.99 (br s), 6.83 (br s), 6.83 (br s), 6.01 (d, *J* = 1.8 Hz), 5.97 (d, *J* = 1.8 Hz), 4.84 (s), 4.23 (m), 2.87 (dd, *J* = 16.8, 4.5 Hz), 2.71 (dd, *J* = 16.8, 3.1 Hz)	1	(Abd El-Razek, 2007)
**6**	isorhamnetin-7-*O*-rutinoside	1.09 (d, *J* = 6.2 Hz)	7.89 (d, *J* = 1.8 Hz), 7.64 (dd, *J* = 8.7, 1.8 Hz), 6.97 (d, *J* = 8.7 Hz), 6.46 (s), 6.25 (s), 5.14 [a], 5.13 [a], 3.95 (s), 3.79 (d, *J* = 10.9 Hz), 3.70 – 3.20 [a] (sugar protons), 1.09 (d, *J* = 6.2 Hz),	1	(Mohamed et al., 2020)
**7**	carnosic acid	6.44 (s)	3.40 (m), 3.15 (m), 2.72 (m), 2.54 (m), 1.87 (m), 1.77 (m), 1.44 – 1.60 [a], 1.29 [a], 1.15 (s), 1.14 (s), 0.96 (s), 0.93 (s)	1	(Pukalskas et al., 2005)
**8**	diosmetin	3.94 (s)	7.54 (dd, *J* = 8.5, 2.3 Hz), 7.41 (d, *J* = 2.3 Hz), 7.11 (d, *J* = 8.5 Hz), 6.62 (s), 6.50 (d, *J* = 2.3 Hz), 6.24 (d, *J* = 2.2 Hz)	1	(Park et al., 2007)
**9**	luteolin	6.59 (s)	7.43 (m), 6.96 (dd, *J* = 8.9, 1.6 Hz), 6.69 (s), 6.49 (d, *J* = 2.2 Hz), 6.24 (d, *J* = 2.0 Hz)	1	(Lin et al., 2015)
**10**	scutellarein	6.63 (br s)	7.88 (dd, *J* = 8.4, 2.0 Hz), 6.98 (dd, *J* = 8.4, 1.8 Hz), 6.63 (br s)	1	(Li et al., 2006)
**11**	acacetin	6.69 (s)	7.98 (dd, *J* = 8.5, 1.8 Hz), 7.13 (dd, *J* = 8.5, 2.0 Hz), 6.52 (d, *J* = 2.2 Hz), 6.26 (d, *J* = 2.2 Hz), 3.91 (s)	1	(King-Díaz et al., 2015)
**12**	carnosol	6.73 (s)	5.48 (m), 3.23 (m), 2.77 (m), 2.53 (ddd, *J* = 14.0, 13.9, 4.4 Hz), 2.22 (ddd, *J* = 14.2, 4.9, 4.9 Hz), 1.87 [a], 1.83 [a], 1.68 (m), 1.61 (m), 1.50 (m), 1.31 (m), 1.19 (d, *J* = 3.6 Hz), 1.18 (d, *J* = 3.6 Hz), 0.86 (s), 0.85 (s)	1	(Pukalskas et al., 2005)
**13**	catechin hydrate	6.83 (d, *J* = 8.2 Hz)	6.86 (d, *J* = 2.0 Hz), 6.75 (dd, *J* = 8.2, 2.1 Hz), 5.99 (br s), 5.90 (br s), 4.63 (d, *J* = 7.4 Hz), 4.05 (m), 2.83 (dd, *J* = 16.2, 5.41 Hz), 2.51 (dd, *J* = 16.2, 7.8 Hz),	1	(Kiehlmann and Tracey, 1986)
**14**	gallic acid	7.05 (s)	-	1	(López-Martínez et al., 2015)
**15**	rosmarinic acid	6.80 (s)	7.50 (d, *J* = 16.0 Hz), 7.09 50 (d, *J* = 2.0 Hz), 6.98 (dd, *J* = 8.2, 2.0 Hz), 6.83 (d, *J* = 8.2 Hz), 6.80 (br s), 6.73 (d, *J* = 8.1 Hz), 6.67 (dd, *J* = 8.1, 2.0	1	(Lecomte et al., 2010)

			Hz), 6.29 (d, *J* = 16.0 Hz), 5.03 (dd, *J* = 10.0, 3.3 Hz), 3.09 (dd, *J* = 14.5, 3.3 Hz), 2.92 (dd, *J* = 14.5, 10.0 Hz)		
**16**	ferulic acid	7.07 (dd, *J* = 8.2, 1.5 Hz)	7.46 (d, *J* = 15.9 Hz), 7.18 (d, *J* = 1.5 Hz), 6.85 (d, *J* = 8.2 Hz), 6.34 (d, *J* = 15.9 Hz), 3.89 (s)	1	(López-Martínez et al., 2015)
**17**	caffeic acid	7.42 (d, *J* = 15.9 Hz)	7.07 (d, *J* = 1.7 Hz), 6.96 (d, *J* = 8.3 Hz)	1	(López-Martínez et al., 2015)
**18**	*p*-coumaric acid	7.52 (d, *J* = 15.9 Hz)	7.48 (dd, *J* = 8.2, 1.0 Hz), 6.85 (dd, *J* = 8.2, 1.2 Hz), 6.32 (d, *J* = 15.9 Hz)	1	(López-Martínez et al., 2015)
**19**	chlorogenic acid	7.59 (d, *J* = 15.9 Hz)	7.11 (d, *J* = 1.6 Hz), 7.01 (dd, *J* = 8.3, 1.6 Hz), 6.84 (dd, *J* = 8.3, 1.6 Hz), 6.34 (d, *J* = 15.9 Hz), 5.34 (ddd, *J* = 10.7, 10.7, 1.9 Hz), 4.16 (ddd, *J* = 4.0, 3.5, 3.4 Hz), 3.75 (dd, *J* = 9.0, 3.3 Hz), 2.14 [a], 2.10 [a], 2.01 [a], 1.98 [a.]	1	(Pauli et al., 1999)
**20**	rutin	6.94 (d, *J* = 8.5 Hz)	7.67 (d, *J* = 2.2 Hz), 7.61 (dd, *J* = 8.5, 2.3 Hz), 6.46 (d, *J* = 2.1 Hz), 6.25 (d, *J* = 2.1 Hz), 5.04 (d, *J* = 7.7 Hz), 4.53 (br s), 3.80 – 3.25 [a] (sugar protons), 1.11 (d, *J* = 6.2 Hz)	1	(Napolitano et al., 2012)
**21**	quercetin	7.72 (d, *J* = 2.1 Hz)	7.63 (dd, *J* = 8.5, 2.1 Hz), 6.95 (d, *J* = 8.5 Hz), 6.46 (d, *J* = 1.9 Hz), 6.24 (d, *J* = 1.9 Hz)	1	(Napolitano et al., 2012)
**22**	apigenin	7.89 (dd, *J* = 8.5, 1.2 Hz)	6.98 (dd, *J* = 8.5, 1.2 Hz), 6.64 (s), 6.50 (d, *J* = 2.2 Hz), 6.24 (d, *J* = 2.1 Hz)	1	(Shen et al., 1993)
**23**	isorosmanol	1.42 (m)	7.88 (s), 6.98 (s), 4.16 (dd, *J* = 11.5, 4.2 Hz), 4.07 (dd, *J* = 11.5, 6.2 Hz), 3.23 (m), 2.39 (m), 1.99 [a], 1.97 [a], 1.60 (m), 1.23 (s), 1.22 (s)	1	(Pukalskas et al., 2005)
**24**	apigenin-7-*O*-β-D-glycoside	7.92 (dd, *J* = 8.4, 1.2 Hz)	6.99 (dd, *J* = 8.4, 1.2 Hz), 6.86 (d, *J* = 2.1 Hz), 6.55 (d, *J* = 2.1 Hz), 5.14 (d, *J* = 7.1 Hz), 3.94 (br d, *J* = 10.4 Hz), 3.75 (dd, *J* = 12.4, 5.6 Hz), 3.65-3.42 [a] (sugar protons)	1	(Liu et al., 2012; Peng et al., 2016)
**25**	kaempferol	8.07 (dd, *J* = 8.4, 1.2 Hz)	6.96 (dd, *J* = 8.4, 1.2 Hz), 6.46 (d, *J* = 2.1 Hz), 6.24 (d, *J* = 2.2 Hz)	1	(Napolitano et al., 2012)
**26**	isorhamnetin-3-*O*-β-D-rutinoside (narcissin)	6.97 (dd, *J* = 8.0, 2.0 Hz)	7.89 (d, *J* = 2.0 Hz), 7.64 (d, *J* = 8.0 Hz), 6.44 (d, *J* = 1.8 Hz), 6.23 (d, *J* = 1.8 Hz), 5.14 (d, *J* = 7.4 Hz), 4.53 (br s), 3.95 (s), 3.90 – 3.26 [a] (sugar protons), 1.09 (d, *J* = 6.2 Hz)	1	(Rastrelli et al., 1995)
**27**	epiisorosmanol	5.21 (d, *J* = 4.3 Hz)	6.81 (s), 4.34 (m), 3.23 (m), 2.73 (br d, *J* = 14.4 Hz), 2.54 (ddd, *J* = 14.4, 14.2, 4.4 Hz) 1.80 (m), 1.61 (m),1.38-1.52 [a], 1.21 (br s), 1.19 (br s), 1.02 (s), 0.88 (s)	1	(Pukalskas et al., 2005)

[a] Overlapped signals
[b] MSI level of identification according to Sumner et al. (Sumner et al., 2007)

Table S6. Selected ^{1}H NMR chemical shifts (δ) and coupling constants (Hz) of Chenomx 500 MHz version 11 library metabolites (CL).

CL number	metabolite	selected characteristic NMR chemical shifts in ppm (multiplicity, *J*)	other chemical shifts in ppm (multiplicity, *J*)	MSI Status [a]	references
**1**	alanine	1.47 (d, *J* = 7.3 Hz)	3.78 (m)	2	(Govindaraju et al., 2000; Xiao et al., 2008)
**2**	acetate	1.91 (s)		2	(Govindaraju et al., 2000; Xiao et al., 2008)
**3**	malate	4.30 (d, *J* = 10.2, 3.1 Hz)	2.68 (dd, *J* = 15.4, 3.1 Hz), 2.37 (dd, *J* = 15.4, 10.2 Hz),	2	(Xiao et al., 2008)
**4**	malonate	3.12 (s)		2	(Xiao et al., 2008)
**5**	choline	4.06 (m)	3.50 (m), 3.19 (m)	2	(Govindaraju et al., 2000; Deborde et al., 2021)
**6**	fructose	4.11 (d, *J* = 4.0 Hz)	3.88 (dd, *J* = 10.0, 3.4 Hz)	2	(Xiao et al., 2008)
**7**	sucrose	4.21 (d, *J* = 8.7 Hz)	5.40 (d, *J* = 3.9 Hz), 4.04 (t, *J* = 8.4, 8.4 Hz)	2	(Xiao et al., 2008)
**8**	fumarate	6.51 (s)		2	(Xiao et al., 2008)
**9**	valine	0.98 (d, *J* = 7.0 Hz) and 1.03 (d, *J* = 7.0 Hz)	3.60 (d, *J* = 4.4 Hz), 2.26 (m)	2	(Govindaraju et al., 2000; Xiao et al., 2008)
**10**	proline	3.41 (m)	4.14 (m), 3.32 (m), 2.34 (m), 2.02 (m), 1.97 (m)	2	10
**11**	asparagine	4.00 (m)	2.96 (dd, *J* = 16.1, 4.6 Hz), 2.86 (dd, *J* = 16.1, 7.6 Hz),	2	(Xiao et al., 2008)

[a] MSI level of identification according to Sumner et al. (Sumner et al., 2007)

Table S7. Pearson correlation coefficients among 500 MHz version 11 Chenomx library metabolites (CL). [a]

	**alanine**	**acetate**	**malate**	**malonate**	**choline**	**fructose**	**sucrose**	**fumarate**	**valine**	**proline**	**asparagine**
**alanine**	1.000	–	–	–	–	–	–	–	–	–	–
**acetate**	-0.144	1.000	–	–	–	–	–	–	–	–	–
**malate**	0.084	0.133	1.000	–	–	–	–	–	–	–	–
**malonate**	0.159	-0.197	0.307	1.000	–	–	–	–	–	–	–
**choline**	-0.050	-0.202	0.005	**0.581**	1.000	–	–	–	–	–	–
**fructose**	**0.383**	0.298	-0.144	0.015	0.160	1.000	–	–	–	–	–
**sucrose**	0.243	**-0.510**	-0.030	0.197	**0.447**	0.279	1.000	–	–	–	–
**fumarate**	**0.405**	-0.289	0.218	**0.404**	0.054	-0.019	0.141	1.000	–	–	–
**valine**	**0.664**	0.048	0.154	**0.563**	**0.312**	0.117	0.071	0.279	1.000	–	–
**proline**	-0.087	0.021	0.074	**0.625**	**0.828**	0.150	0.182	-0.066	**0.406**	1.000	–
**asparagine**	0.072	-0.091	0.106	**0.747**	**0.872**	0.250	0.250	0.089	**0.494**	**0.919**	1.000

[a] significant values (p<0.05) are reported in bold character.

Table S8. Pearson correlation coefficients among custom 500 MHz version 11 Chenomx library (CCL) metabolites. [a]

	**12-*O*-methylcarnosic acid**	**7-*O*-methoxyrosmanol**	**rosmarinate**	**genkwanin**	**epicatechin**	**isorhamnetin-7-*O*-rutinoside**	**carnosic acid**	**diosmetin**	**luteolin**	**scutellarein**	**acacetin**	**carnosol**	**catechin hydrate**	**gallic acid**
**12-*O*-methylcarnosic acid**	1.000	–	–	–	–	–	–	–	–	–				
**7-*O*-methylrosmanol**	**0.787**	1.000	–	–	–	–	–	–	–	–				
**methylrosmarinate**	**0.588**	**0.593**	1.000	–	–	–	–	–	–	–				
**genkwanin**	**0.773**	**0.822**	**0.503**	1.000	–	–	–	–	–	–				
**epicatechin**	**0.524**	0.193	0.254	0.346	1.000	–	–	–	–	–				
**isorhamnetin-7-*O*-rutinoside**	0.253	**0.421**	0.085	**0.381**	0.066	1.000	–	–	–	–				
**carnosic acid**	-0.032	0.130	-0.124	0.035	0.025	**0.733**	1.000	–	–	–				
**diosmetin**	**0.546**	**0.509**	0.121	**0.799**	0.332	**0.467**	0.185	1.000	–	–				
**luteolin**	-0.118	-0.099	-0.129	0.018	-0.086	-0.037	0.078	-0.083	1.000	–				
**scutellarein**	0.208	0.110	0.336	0.084	0.252	0.150	0.183	0.043	0.327	1.000				
**acacetin**	**0.533**	**0.638**	**0.898**	**0.464**	0.056	0.102	-0.159	0.054	-0.098	0.329	1.000			
**carnosol**	**0.546**	**0.746**	**0.832**	**0.567**	0.010	0.237	-0.066	0.188	-0.124	0.232	**0.925**	1.000		
**catechin hydrate**	**0.589**	**0.411**	**0.793**	**0.371**	**0.394**	-0.039	-0.226	0.061	-0.019	**0.530**	**0.780**	**0.630**	1.000	
**gallic acid**	**0.416**	**0.387**	**0.819**	0.343	0.178	0.060	0.019	0.091	0.075	**0.466**	**0.611**	**0.549**	**0.590**	1.000
**rosmarinic acid**	**0.598**	**0.583**	**0.911**	**0.454**	0.250	0.111	-0.165	0.062	-0.099	**0.407**	**0.943**	**0.836**	**0.884**	**0.620**
**ferulic acid**	**0.544**	**0.612**	**0.977**	**0.470**	0.139	0.092	-0.158	0.063	-0.143	0.281	**0.947**	**0.886**	**0.765**	**0.766**
**caffeic acid**	0.035	-0.064	0.269	-0.078	0.194	-0.033	0.096	-0.067	0.123	**0.673**	0.226	0.136	**0.446**	**0.379**
**coumaric acid**	**0.561**	**0.663**	**0.916**	**0.492**	0.133	0.120	-0.167	0.071	-0.170	0.220	**0.970**	**0.914**	**0.747**	**0.580**
**chlorogenic acid**	**0.655**	**0.672**	**0.940**	**0.546**	0.311	0.170	-0.095	0.149	-0.071	**0.398**	**0.911**	**0.829**	**0.819**	**0.715**
**rutin**	**0.602**	**0.589**	**0.987**	**0.491**	0.270	0.101	-0.132	0.106	-0.114	**0.377**	**0.918**	**0.832**	**0.839**	**0.780**
**quercetin**	-0.012	-0.022	0.117	-0.030	0.028	0.037	0.069	-0.041	0.049	0.289	0.127	0.159	0.184	0.190
**apigenin**	0.195	0.157	**0.444**	0.173	**0.395**	0.206	0.256	0.064	0.180	**0.482**	0.231	0.187	0.310	**0.674**
**isorosmanol**	-0.212	-0.200	-0.194	-0.010	-0.149	-0.230	-0.207	-0.225	**0.570**	-0.174	-0.175	-0.136	-0.177	-0.170
**apigenin-7-*O*-β-D-glycoside**	0.333	0.294	**0.517**	0.281	**0.403**	0.242	0.256	0.137	0.197	**0.555**	0.321	0.268	**0.401**	**0.740**
**kaempferol**	0.247	0.207	**0.427**	0.257	**0.438**	0.222	0.220	0.129	0.050	**0.357**	0.198	0.214	0.287	**0.554**
**isorhamnetin-3-*O*-rutinoside**	**0.604**	**0.588**	**0.983**	**0.486**	0.263	0.092	-0.135	0.104	-0.097	**0.402**	**0.922**	**0.832**	**0.859**	**0.789**
**epiisorosmanol**	-0.302	**-0.364**	-0.300	-0.101	f0.261	-0.322	**-0.527**	-0.010	0.107	-0.317	-0.258	-0.255	-0.233	-0.286

	**rosmarinic acid**	**ferulic acid**	**caffeic acid**	**coumaric acid**	**chlorogenic acid**	**rutin**	**quercetin**	**apigenin**	**isorosmanol**	**apigenin-7-*O*-β-D-glycoside**	**kaempferol**	**isorhamnetin-3-*O*-rutinoside**	**epirosmanol**
**12-*O*-methylcarnosic acid**	–	–	–	–	–	–	–	–	–	–	–	–	–
**7-*O*-methylrosmanol**	–	–	–	–	–	–	–	–	–	–	–	–	–
**methylrosmarinate**	–	–	–	–	–	–	–	–	–	–	–	–	–
**genkwanin**	–	–	–	–	–	–	–	–	–	–	–	–	–
**epicatechin**	–	–	–	–	–	–	–	–	–	–	–	–	–
**isorhamnetin-7-*O*-rutinoside**	–	–	–	–	–	–	–	–	–	–	–	–	–
**carnosic acid**	–	–	–	–	–	–	–	–	–	–	–	–	–
**diosmetin**	–	–	–	–	–	–	–	–	–	–	–	–	–
**luteolin**	–	–	–	–	–	–	–	–	–	–	–	–	–
**scutellarein**	–	–	–	–	–	–	–	–	–	–	–	–	–
**acacetin**	–	–	–	–	–	–	–	–	–	–	–	–	–
**carnosol**	–	–	–	–	–	–	–	–	–	–	–	–	–
**catechin hydrate**	–	–	–	–	–	–	–	–	–	–	–	–	–
**gallic acid**	–	–	–	–	–	–	–	–	–	–	–	–	–
**rosmarinic acid**	1.000	–	–	–	–	–	–	–	–	–	–	–	–
**ferulic acid**	**0.934**	1.000	–	–	–	–	–	–	–	–	–	–	–
**caffeic acid**	0.292	0.226	1.000	–	–	–	–	–	–	–	–	–	–
**coumaric acid**	**0.947**	**0.961**	0.169	1.000	–	–	–	–	–	–	–	–	–
**chlorogenic acid**	**0.943**	**0.939**	0.288	**0.934**	1.000	–	–	–	–	–	–	–	–
**rutin**	**0.951**	**0.976**	0.317	**0.936**	**0.962**	1.000	–	–	–	–	–	–	–
**quercetin**	0.109	0.117	**0.559**	0.090	0.110	0.129	1.000	–	–	–	–	–	–
**apigenin**	0.272	**0.358**	**0.476**	0.222	**0.423**	**0.418**	**0.365**	1.000	–	–	–	–	–
**isorosmanol**	-0.188	-0.174	-0.114	-0.169	-0.191	-0.189	-0.015	-0.132	1.000	–	–	–	–
**apigenin-7-*O*-β-D-glycoside**	**0.361**	**0.430**	**0.495**	0.298	**0.537**	**0.497**	**0.387**	**0.959**	-0.160	1.000	–	–	–
**kaempferol**	0.247	0.330	**0.394**	0.225	**0.396**	**0.387**	**0.403**	**0.923**	-0.117	**0.879**	1.000	–	–
**isorhamnetin-3-*O*-rutinoside**	**0.954**	**0.975**	0.335	**0.930**	**0.960**	**0.998**	0.145	**0.418**	-0.195	**0.501**	**0.381**	1.000	–
**epiisorosmanol**	-0.282	-0.262	-0.195	-0.257	-0.307	-0.291	-0.092	-0.306	**0.451**	-0.335	-0.291	-0.294	1.000

[a] significant values ($p<0.05$) are reported in bold character.

Preliminary test carried out to define the working concentration for the soft rot assay

This assay was carried out to define the working concentration of extracts (Table S1) and pure compounds to be used during the sof rot assay aiming to evaluate their potential activity in inhibiting the pectolytic activity of *Pectobacterium carotovorum* subsp. *carotovorum*.

P. carotovorum subsp. *carotovorum* was cultured at 28 °C on a nutrient agar medium (NYDA: 8.0 g nutrient broth, 1.5 g glucose, 20.0 g agar, 4.0 g yeast extract, and 1 L deionized water) in Petri dishes for 2 days at 28 °C. The bacterial inoculum was prepared from a 2 day-old cultured bacteria in a vial containing Buffered Pepton Water (Generon-Italy) at the concentration of 10^8 CFU/mL.

Extracts and pure compounds and were dissolved separately in a solution of sterile distilled water and DMSO working solution (1:1 v/v) to obtain, after the inoculation, a concentration of 1000 ppm, 500 ppm, 50 ppm and 0 ppm respectively. These solutions were then inoculated with the bacterial suspension to obtain a final concentration of 10^7 CFU/mL of *P. carotovorum* subsp. *carotovorum*.

After 5 min of exposure, each suspension was quantified by serial dilutions plating method.

Serial dilutions were made in peptone water (from 10^{-1} to 10^{-9}); 0.1 mL of different dilutions were plated on NYDA agar plates in triplicate (3 plates each dilution) and incubated for 2 days at 28°C. Not inoculated solution at 0 ppm was also evaluated to verify the absence of activity of DMSO.

For each dilution, the CFU (Colony Forming Unit) of *P. carotovorum* subsp. *carotovorum* per mL of suspension was obtained. Final CFU of *P. carotovorum* subsp. *carotovorum* per mL of suspension for each single treatment and concentration was calculated as the average of the CFU/mL of the 3 replicates (data related to the dilutions are not reported).

Table S9. Quantification in CFU/mL of *Pectobacterium carotovorum* subsp. *carotovorum* after the exposure to the extracts / pure compounds at 1000 ppm, 500 ppm, 50 ppm and 0 ppm, respectively.

Extracts / pure compounds	Concentration (ppm)	Average of the replicates (CFU/mL)	Standard Deviation	Abbott index (%)[d]
**1**	50	2.70×10^{7}	1.00×10^{6}	0
**1**	500	8.67×10^{6}	2.89×10^{5}	68
**1**	1000	1.90×10^{6}	0.00	93
**3**	50	2.70×10^{7}	1.32×10^{6}	0
**3**	500	2.40×10^{7}	1.00×10^{6}	11
**3**	1000	2.17×10^{7}	1.53×10^{6}	20
**5**	50	2.69×10^{7}	2.20×10^{6}	0
**5**	500	2.37×10^{7}	2.08×10^{6}	12
**5**	1000	2.13×10^{7}	2.52×10^{6}	21
**6**	50	2.67×10^{7}	1.53×10^{6}	1
**6**	500	1.83×10^{7}	7.64×10^{6}	32
**6**	1000	1.50×10^{7}	0.00	44
**7**	50	2.67×10^{7}	1.15×10^{6}	1
**7**	500	2.40×10^{7}	1.00×10^{6}	11
**7**	1000	8.73×10^{6}	6.69×10^{6}	68
**9**	50	2.70×10^{7}	2.65×10^{6}	0
**9**	500	2.37×10^{7}	4.04×10^{6}	12
**9**	1000	1.37×10^{7}	7.29×10^{6}	49
**16**	50	2.68×10^{7}	7.64×10^{5}	1
**16**	500	2.47×10^{7}	5.77×10^{5}	9
**16**	1000	1.34×10^{7}	7.54×10^{6}	50
**20**	50	2.67×10^{7}	1.15×10^{6}	1
**20**	500	2.33×10^{7}	1.53×10^{6}	14
**20**	1000	9.67×10^{6}	5.77×10^{5}	64
**22**	50	2.68×10^{7}	1.26×10^{6}	1
**22**	500	1.77×10^{7}	6.81×10^{6}	35
**22**	1000	1.15×10^{7}	3.01×10^{6}	57
**26**	50	2.67×10^{7}	3.21×10^{6}	1
**26**	500	2.40×10^{7}	1.00×10^{6}	11
**26**	1000	1.21×10^{7}	3.35×10^{6}	55
**28**	50	2.65×10^{7}	5.00×10^{5}	2

**28**	500	$2.07x10^{7}$	$5.77x10^{5}$	23
**28**	1000	$7.93x10^{6}$	$9.29x10^{5}$	71
**32**	50	$2.60x10^{7}$	$5.20x10^{6}$	4
**32**	500	$1.80x10^{7}$	$2.65x10^{6}$	33
**32**	1000	$1.04x10^{7}$	$8.92x10^{6}$	61
**isorosmanol**	50	$2.69x10^{7}$	$3.82x10^{5}$	0
**isorosmanol**	500	$2.05x10^{7}$	$8.66x10^{5}$	24
**isorosmanol**	1000	$8.08x10^{6}$	$7.22x10^{5}$	70
**12-*O*-methylcarnosic acid**	50	$2.67x10^{7}$	$7.64x10^{6}$	1
**12-*O*-methylcarnosic acid**	500	$1.83x10^{7}$	$1.53x10^{6}$	32
**12-*O*-methylcarnosic acid**	1000	$1.06x10^{7}$	$8.71x10^{6}$	61
**carnosic acid**	50	$2.63x10^{7}$	$5.77x10^{5}$	2
**carnosic acid**	500	$2.03x10^{7}$	$6.43x10^{6}$	25
**carnosic acid**	1000	$1.11x10^{7}$	$8.78x10^{6}$	59
**carnosol**	50	$2.67x10^{7}$	$5.77x10^{5}$	1
**carnosol**	500	$2.40x10^{7}$	$1.00x10^{6}$	11
**carnosol**	1000	$9.93x10^{6}$	$1.15x10^{5}$	63
**7-*O*-methylrosmanol**	50	$2.63x10^{7}$	$3.06x10^{6}$	2
**7-*O*-methylrosmanol**	500	$1.29X10^{7}$	$5.25x10^{6}$	52
**7-*O*-methylrosmanol**	1000	$7.03x10^{6}$	$8.62x10^{5}$	74
**DW (PC)[a]**	0	$2.70x10^{7}$	$1.73x10^{6}$	0
**DW (NC)[b]**	0	$2.70x10^{7}$	$2.00x10^{6}$	0

The test was performed in 3 replicates. [a] DW (PC): inoculated distilled water/DMSO working solution 1:1 v/v (Positive Control); [b] DW (NC): not inoculated distilled water/DMSO working solution 1:1 v/v (Negative Control); [c] different letters indicate significant difference among treatments ($p \leq 0.05$, Tukey HSD test), [d] Abbott index % = (average CFU/mL x 100) / average CFU/mL of PC.

The exposure for 5 minutes of a liquid suspension of *P. carotovorum* subsp. *carotovorum* (10^{7} CFU/mL) to a solution of the treatments at the concentration of 50 ppm did not significantly inhibit the growth of the bacteria.
The exposures at the concentration of 500 ppm and 1000 ppm inhibited the growth of this bacteria from 9% to 68% and from 20% to 93%, respectively, compared to the untreated control (PC).
The concentration of 1000 ppm was then selected as working concentration for further assay.

Table S10. Disk diffusion test.

Extracts / pure compounds	Concentration (ppm and mg/mL)						
	**0 ppm 0 mg/mL**	**16 ppm 0.016 mg/mL**	**50 ppm 0.050 mg/mL**	**250 ppm 0.250 mg/mL**	**500 ppm 0.500 mg/mL**	**1000 ppm 1 mg/mL**	**2000 ppm 2 mg/mL**
**1**	-	-	-	7.7±0.6 (-)	9.3±0.6 (+)	14±1.0 (+)	17.3±1.5 (++)
**3**	-	-	-	-	-	7.0±0.0 (-)	9.0±0.0 (+)
**5**	-	-	-	-	-	-	9.0±0.0 (+)
**6**	-	-	-	7.0±0.0 (-)	8.3±0.6 (-)	9.3±0.6 (+)	11.3±2.1 (+)
**7**	-	-	-	7.7±0.6 (-)	9.3±0.6 (+)	11±1.0 (+)	12.0±1.0 (+)
**9**	-	-	-	-	-	-	9.0±0.0 (+)
**16**	-	-	-	-	10.3±0.6 (+)	13.3±0.6 (+)	16.3±1.2 (++)
**20**	-	-	-	8.0±1.0 (-)	10.0±0.0 (+)	12.3±0.6 (+)	12.7±0.6 (+)
**22**	-	-	-	8.7±0.6 (-)	10.7±0.6 (+)	11.3±0.6 (+)	13.3±0.6 (+)
**26**	-	-	-	11.7±0.6 (+)	13±0.0 (+)	13.3±0.6 (+)	14.7±0.6 (+)
**28**	-	-	-	-	10.3±0.6 (+)	11.3±0.6 (+)	13.3±0.6 (+)
**32**	-	-	-	-	-	-	9.0±0.0 (+)

**isorosmanol**	-	-	-	9.3±0.6 (+)	12±0.0 (+)	14±1.0 (+)	16.3±0.6 (++)
**12-*O*-methylcarnosic acid**	-	-	-	7.3±0.6 -)	8.3±0.6 (-)	11±1.0 (+)	14.3±0.6 (+)
**carnosic acid**	-	-	7.3±0.6 (-)	9.0±0.0 (+)	12±0.0 (+)	15±1.0 (+)	16.7±0.6 (++)
**carnosol**	-	-	7.0±0.0 (-)	8.7±0.6 (+)	12±0.0 (+)	13.5±0.7 (+)	15.3±0.6 (++)
**7-*O*-methylrosmanol**	-	-	-	-	-	9±0.0 (+)	11.7±0.6 (+)

Diameter in mm of the growth inhibition zone of *Pectobacterium carotovorum* subsp. *carotovorum* after the exposure to the extracts / pure compounds at 2000 ppm, 1000 ppm, 500 ppm, 250 ppm, 50 ppm, 16 ppm and 0 ppm, respectively, expressed as the average of three independent repetitions with a standard deviation value and the classification of sensitivity (-), (+) or (++).
Although all extracts and pure compounds at concentrations ranging from 250 ppm to 2000 ppm can inhibit *P. carotovorum* subsp. *carotovorum*, extracts 1 and 16 and the pure compounds isorosmanol, carnosic acid and carnosol show higher antimicrobial potency at 2000 ppm against this microorganism.

**Reference compound**	**Concentration (ppm and mg/mL)**			
	**0 ppm**<br>**0 mg/mL**	**1 ppm**<br>**0.001 mg/mL**	**8 ppm**<br>**0.008 mg/mL**	**16 ppm**<br>**0.016 mg/mL**
**ampicillin**	-	14.3±0.6(+)	16±1.0(++)	19±0.6(++)

Diameter in mm of the growth inhibition zone of *Pectobacterium carotovorum* subsp. *carotovorum* after the exposure to ampicillin at 16, 8 and 1 ppm and 0 ppm expressed as the average of three independent repetitions with a standard deviation value and the classification of sensitivity (-), (+) or (++).

è

References

Abd El-Razek, M.H. (2007). NMR assignments of four catechin epimers. *Asian Journal of Chemistry* 19**,** 4867-4872.

Begum, A., Sandhya, S., Shaffath Ali, S., Vinod, K.R., Reddy, S., and Banji, D. (2013). An in-depth review on the medicinal flora *Rosmarinus officinalis* (Lamiaceae). *Acta Scientiarum Polonorum. Technologia Alimentaria* 12 1**,** 61-73.

Cervelli, C., and Masselli, L. (Year). "Characterization of rosemary cultivars for ornamental purposes", in: *VII International Symposium on New Floricultural Crops*, eds. G. Facciuto & M.I. Sánchez: ISHS Acta Horticulturae), 107-114.

Correia, F.C.S., Targanski, S.K., D., B.T.R., da Silva, Y.S.A.D., Violante, I.M.P., de Carvalho, M.G., et al. (2020). Chemical constituents and antimicrobial activity of branches and leaves of *Cordia insignis* (Boraginaceae). *Revista virtual de Quimica* 12(3)**,** 809-816.

CPVO (2022). "CPVO Variety Finder, Commercial registers". (Angers, France: Community Plant Variety Office).

Gomes, R., Ramirez, R., Maciel, J., Agra, M., Souza, M., Falcão-Silva, V., et al. (2011). Phenolic compounds from *Sidastrum micranthum* (A. St.-Hil.) Fryxell and evaluation of acacetin and 7,4'-di-*O*-methylisoscutellarein as motulator of bacterial drug resistence. *Química Nova* 34**,** 1385-1388. doi: 10.1590/S0100-40422011000800016.

Govindaraju, V., Young, K., and Maudsley, A.A. (2000). Proton NMR chemical shifts and coupling constants for brain metabolites. *NMR in Biomedicine* 13(3)**,** 129-153. doi: 10.1002/1099-1492(200005)13:3<129::aid-nbm619>3.0.co;2-v.

Jeanmonod, D. (2015). Notes à La Flore de Corse, XXV. *Candollea* 70(1)**,** 109-140.

Kiehlmann, E., and Tracey, A.S. (1986). Proton magnetic resonance spectra of catechin and bromocatechin derivatives: C6- vs. C8-substitution. *Canadian Journal of Chemistry* 64(10)**,** 1998-2005. doi: 10.1139/v86-330.

King-Díaz, B., Granados-Pineda, J., Bah, M., Rivero-Cruz, J.F., and Lotina-Hennsen, B. (2015). Mexican propolis flavonoids affect photosynthesis and seedling growth. *Journal of Photochemistry and Photobiology B: Biology* 151**,** 213-220. doi: https://doi.org/10.1016/j.jphotobiol.2015.08.019.

Kuo, Y.-H., Lee, S.-M., and Lai, J.-S. (2000). Constituents of the whole herb of *Clinoponium laxiflorum*. *Journal of the Chinese Chemical Society* 47(1)**,** 241-246. doi: https://doi.org/10.1002/jccs.200000028.

Lecomte, J., Giraldo, L.J.L., Laguerre, M., Baréa, B., and Villeneuve, P. (2010). Synthesis, characterization and free radical scavenging properties of rosmarinic acid fatty esters. *Journal of the American Oil Chemists' Society* 87(6)**,** 615-620. doi: https://doi.org/10.1007/s11746-010-1543-8.

Li, N., Huang, J., Weng, W., Su, S., and Yu, Y. (2006). Preparation and identification of scutellarein by HPLC with an enzymolysis reaction. *Journal of Liquid Chromatography & Related Technologies* 29(9)**,** 1297-1305. doi: 10.1080/10826070600598878.

Lin, L.-C., Pai, Y.-F., and Tsai, T.-H. (2015). Isolation of luteolin and luteolin-7-*O*-glucoside from *Dendranthema morifolium* Ramat Tzvel and their pharmacokinetics in rats. *Journal of Agricultural and Food Chemistry* 63(35), 7700-7706. doi: 10.1021/jf505848z.

Liu, J., Chen, L., Cai, S., and Wang, Q. (2012). Semisynthesis of apigenin and acacetin-7-*O*-β-D-glycosides from naringin and their cytotoxic activities. *Carbohydrate Research* 357, 41-46. doi: https://doi.org/10.1016/j.carres.2012.05.013.

López-Martínez, L., Santacruz, H., Navarro, R., Sotelo-Mundo, R., and Aguilar, G. (2015). A 1H NMR Investigation of the interaction between phenolic acids found in mango (*Manguifera indica* cv Ataulfo) and papaya (*Carica papaya* cv Maradol) and 1,1-diphenyl- 2-picrylhydrazyl (DPPH) Free Radicals. *PLoS ONE* 10, e0140242. doi: 10.1371/journal.pone.0140242.

Malcuit, G., and Deleuil, G. (1957). A propos de nouvelles localités de Pin d'Alep en Corse. *Bulletin de la Société Botanique de France* 104(7-8), 527-529. doi: 10.1080/00378941.1957.10835141.

Mohamed, E.M., Hetta, M.H., Rateb, M.E., Selim, M.A., M. AboulMagd, A.A., Badria, A.F., et al. (2020). Bioassay-guided isolation, metabolic profiling, and docking studies of hyaluronidase inhibitors from *Ravenala madagascariensis*. *Molecules* 25(7), 1714. doi: 10.3390/molecules25071714.

Napolitano, J.G., Lankin, D.C., Chen, S.N., and Pauli, G.F. (2012). Complete 1H NMR spectral analysis of ten chemical markers of *Ginkgo biloba*. *Magnetic Resonance in Chemistry* 50(8), 569-575. doi: 10.1002/mrc.3829.

Paradis, G. (2004). 32èmes sessions extraordinaires - 2003- le nord de la Corse *Bulletin de la Société Botanique du Centre-Ouest. Nouvelle Série* 35, 419-562.

Park, Y., Moon, B.H., Yang, H., Lee, Y., Lee, E., and Lim, Y. (2007). Complete assignments of NMR data of 13 hydroxymethoxyflavones. *Magnetic Resonance in Chemistry* 45(12), 1072-1105. doi: 10.1002/mrc.2063.

Pauli, G.F., Kuczkowiak, U., and Nahrstedt, A. (1999). Solvent effects in the structure dereplication of caffeoyl quinic acids. *Magnetic Resonance in Chemistry* 37(11), 827-836. doi: https://doi.org/10.1002/(SICI)1097-458X(199911)37:11<827::AID-MRC568>3.0.CO;2-W.

Peng, H.Y., Zhang, X.H., and Xu, J.Z. (2016). Apigenin-7-*O*-β-D-glycoside isolation from the highly copper-tolerant plant *Elsholtzia splendens*. *Journal of Zhejiang University-SCIENCE B (Biomedicine & Biotechnology)* 17(6), 447-454. doi: 10.1631/jzus.B1500242.

Pukalskas, A., van Beek, T.A., and de Waard, P. (2005). Development of a triple hyphenated HPLC–radical scavenging detection–DAD–SPE–NMR system for the rapid identification of antioxidants in complex plant extracts. *Journal of Chromatography A* 1074(1), 81-88. doi: https://doi.org/10.1016/j.chroma.2005.03.089.

Rastrelli, L., Saturnino, P., Schettino, O., and Dini, A. (1995). Studies on the constituents of *Chenopodium pallidicaule* (Canihua) seeds. Isolation and characterization of two new flavonol glycosides. *Journal of Agricultural and Food Chemistry* 43(8), 2020-2024. doi: 10.1021/jf00056a012.

RHS (2022). "Plants". (London, UK: Royal Horticultural Society).

Richheimer, S.L., Bernart, M.W., King, G.A., Kent, M.C., and Beiley, D.T. (1996). Antioxidant activity of lipid-soluble phenolic diterpenes from rosemary. *Journal of the American Oil Chemists' Society* 73(4), 507-514. doi: https://doi.org/10.1007/BF02523927.

Shen, C.-C., Chang, Y.-S., and Hott, L.-K. (1993). Nuclear magnetic resonance studies of 5,7-dihydroxyflavonoids. *Phytochemistry* 34(3), 843-845. doi: https://doi.org/10.1016/0031-9422(93)85370-7.

Sumner, L.W., Amberg, A., Barrett, D., Beale, M.H., Beger, R., Daykin, C.A., et al. (2007). Proposed minimum reporting standards for chemical analysis Chemical Analysis Working Group (CAWG) Metabolomics Standards Initiative (MSI). *Metabolomics* 3(3), 211-221. doi: 10.1007/s11306-007-0082-2.

UPOV (2018). "Revision of document TGP/14: Section 2: Botanical terms: Subsection 3: Color: Annex: Color names for the rhs colour chart", in: *TC/49/36.* (ed.) I.u.f.t.p.o.n.v.o. plants. (Geneva).

Xiao, C., Dai, H., Liu, H., Wang, Y., and Tang, H. (2008). Revealing the metabonomic variation of rosemary extracts using 1H NMR spectroscopy and multivariate data analysis. *Journal of Agricultural and Food Chemistry* 56(21), 10142-10153. doi: 10.1021/jf8016833.

Yolcubal, I., Brusseau, M.L., Artiola, J.F., Wierenga, P., and Wilson, L.G. (2004). "12 - Environmental physical properties and processes," in *Environmental Monitoring and Characterization,* eds. J.F. Artiola, I.L. Pepper & M.L. Brusseau. (Burlington: Academic Press), 207-239.
